# An Arabidopsis lipid map reveals differences between tissues and dynamic changes throughout development

**DOI:** 10.1111/tpj.15278

**Published:** 2021-05-24

**Authors:** Cheka Kehelpannala, Thusitha Rupasinghe, Asher Pasha, Eddi Esteban, Thomas Hennessy, David Bradley, Berit Ebert, Nicholas J. Provart, Ute Roessner

**Affiliations:** ^1^ School of BioSciences The University of Melbourne Melbourne VIC 3010 Australia; ^2^ Sciex 2 Gilda Ct Mulgrave VIC 3170 Australia; ^3^ Department of Cell and Systems Biology/Centre for the Analysis of Genome Evolution and Function University of Toronto Toronto Ontario M5S 3B2 Canada; ^4^ Agilent Technologies Australia Pty Ltd 679 Springvale Road Mulgrave VIC 3170 Australia

**Keywords:** *Arabidopsis thaliana*, plant growth, lipid identification, lipid fragments, liquid chromatography mass spectrometry, untargeted lipid analysis, lipidomics

## Abstract

Mass spectrometry is the predominant analytical tool used in the field of plant lipidomics. However, there are many challenges associated with the mass spectrometric detection and identification of lipids because of the highly complex nature of plant lipids. Studies into lipid biosynthetic pathways, gene functions in lipid metabolism, lipid changes during plant growth and development, and the holistic examination of the role of plant lipids in environmental stress responses are often hindered. Here, we leveraged a robust pipeline that we previously established to extract and analyze lipid profiles of different tissues and developmental stages from the model plant *Arabidopsis thaliana*. We analyzed seven tissues at several different developmental stages and identified more than 200 lipids from each tissue analyzed. The data were used to create a web‐accessible *in silico* lipid map that has been integrated into an electronic Fluorescent Pictograph (eFP) browser. This *in silico* library of Arabidopsis lipids allows the visualization and exploration of the distribution and changes of lipid levels across selected developmental stages. Furthermore, it provides information on the characteristic fragments of lipids and adducts observed in the mass spectrometer and their retention times, which can be used for lipid identification. The Arabidopsis tissue lipid map can be accessed at http://bar.utoronto.ca/efp_arabidopsis_lipid/cgi‐bin/efpWeb.cgi.

## Significance Statement

With the complexity of plant lipids, a web database collecting information on plant lipids is of great value. Here the most comprehensive and detailed tissue‐specific lipid profile of *Arabidopsis thaliana* across developmental stages is provided in the form of an open‐source, electronic Fluorescent Pictograph (eFP) browser, along with insights into lipid dynamics across plant growth and development. This resource will significantly advance future research into the Arabidopsis lipidome and facilitate research into other plants.

## INTRODUCTION

Lipids are a group of diverse compounds with crucial roles in all living organisms, tissues and cell types (Fahy *et al.*, 2011). They constitute important structural components of membranes, serve as storage sources for energy, signaling molecules in various biological pathways, and modulators of cellular functions and diseases (Fahy *et al.*, 2011; Mamode Cassim *et al.*, 2019; Sud *et al.*, 2007). Lipids are also involved in many developmental processes, including seed germination (Wang *et al.*, 2016b), organ differentiation (Wang *et al.*, 2016b), pollination (Wolters‐Arts *et al.*, 1998) and environmental stress responses (Cheong *et al.*, 2019, 2020; Welti *et al.*, 2007).

Over the past decade, lipidomics has evolved as a powerful tool to investigate the role of lipids in plants (Welti *et al.*, 2002; Welti and Wang, 2004; Yu *et al.*, 2020). The advances in lipid studies and corresponding technologies have opened up opportunities to address many unanswered questions: for example, how many lipids are present in plants and how the plant lipidome changes during growth and development or in response to environmental stress cues (Wang and Chapman, 2013). Similar to the fields of proteomics and metabolomics, two experimental approaches are widely used in the field of lipidomics: one is an untargeted approach, which aims to analyze all detectable lipids, including unknowns, in an unbiased manner; and the other is the targeted approach, which aims to measure a set of specific lipids quantitatively with greater sensitivity and specificity (Cajka and Fiehn, 2016; Yu *et al.*, 2018). In contrast to the targeted approach, the untargeted approach can provide unexpected new insights into biological systems, such as the detection of novel lipids (Yu *et al.*, 2018).

Mass spectrometry is the main analytical tool used in both untargeted and targeted lipid studies (Cajka and Fiehn, 2016). It involves two main approaches: the direct infusion of the sample into a mass spectrometer, also called shotgun lipidomics; and the separation of sample components by liquid chromatography before injecting them into a mass spectrometer (Hummel *et al.*, 2011). Although both methods have advantages and disadvantages, liquid chromatography mass spectrometry (LC‐MS) is the more popular analytical platform used for the analysis of both polar and non‐polar metabolites and lipids (Cajka and Fiehn, 2016). The approach used widely for untargeted lipidomic studies is ultrahigh performance liquid chromatography coupled with high‐resolution tandem mass spectrometry (UHPLC‐HRMS/MS) (Koelmel *et al.*, 2017). This strategy facilitates lipid identification by accurate mass, MS/MS fragmentation patterns and chromatographic retention times. The high resolution and high mass accuracy of the instrumentation allow the detection of isobaric species that have the same nominal mass but different exact masses (Yu *et al.*, 2018), whereas MS/MS spectra provide structural information on head group type and fatty acid constituents, whereupon isomeric species that often co‐elute can be identified (Koelmel *et al.*, 2017).

Despite recent technological advances and improvements, the characterization of plant lipids remains challenging because of their highly complex nature (Yu *et al.*, 2018). Consequently, the distribution of lipids in different tissues and cell types, as well as their functions in plant growth, development and stress responses, are yet to be fully explored. It has been estimated that the cellular lipidome comprises tens to hundreds of thousands of diverse lipids (Wang *et al.*, 2016a). This diversity is likely to arise from various factors such as the type of head group, aliphatic chain and isomerism, among many others (Wang *et al.*, 2016a). A survey of the plant lipidome in *Hordeum vulgare* (barley) roots identified a total of 49 potential building blocks, including 10 polar head groups, 18 different fatty acids, five long‐chain (sphingoid) bases, 11 sphingolipid head groups and five sterols (Yu *et al.*, 2018), which allow a myriad of combinations for different lipid species.

The complexity of the cellular lipidome is further exacerbated as its composition varies significantly between cell types (Welti and Wang, 2004), subcellular organelles (Holzl and Dormann, 2007; Wewer *et al.*, 2011) and microdomains within membranes (Gronnier *et al.*, 2016). For example, glycerophospholipids, which are considered structural lipids, are most abundant in plasma membranes, tonoplasts and the endoplasmic reticulum (Wewer *et al.*, 2011), whereas diacylglycerolipids, such as monogalactosyl diacylglycerols (MGDGs), digalactosyl diacylglycerols (DGDGs) and sulfoquinovosyl diacylglycerols (SQDGs), are involved in photosynthesis and are therefore most prominent in chloroplast membranes (Holzl and Dormann, 2007). Sterols and sphingolipids, which are considered critical for signal transduction and cell recognition processes, are believed to be the major components of membrane rafts (Gronnier *et al.*, 2016).

Furthermore, the composition of membrane lipids is described to change throughout plant growth and development, is seasonal and time‐of‐day dependent and is affected by different environmental conditions (Colin and Jaillais, 2020). For example, recent studies using lipid imaging have shown that phosphatidylserine levels in plasma membranes change during Arabidopsis root development (Platre *et al.*, 2019) and that the unsaturation of phosphatidylcholines decreases during the day and increases at night (Nakamura *et al.*, 2014). The remodeling of membrane lipids caused by variations in hormone levels (Wang, 2002) and environmental stress conditions, such as heat (Higashi and Saito, 2019), cold (Barrero‐Sicilia *et al.*, 2017; Cheong *et al.*, 2019, 2020), salinity (Sarabia *et al.*, 2018, 2020), pathogen and insect attacks (Farmer *et al.*, 2003), and wounding (Vu *et al.*, 2014), have also been extensively investigated and reported.

Previous studies have demonstrated that the lipid composition varies drastically between different tissues of a plant. For example, an analysis of the *Glycine max* (soybean) leaf lipidome has determined 26.1% of the total lipids analyzed to be DGDGs and 2.9% to be phosphatidylethanolamines (PEs), whereas the root lipidome had 5.1% DGDGs and 26.2% PEs (Narasimhan *et al.*, 2013). In another study, marked differences were found in the distribution of 140 apparent polar lipids across leaves, flower stalks, flowers, siliques, roots and seeds from Arabidopsis, suggesting unique metabolic activities in specific organs of a plant (Devaiah *et al.*, 2006). These studies highlight that a broad coverage of lipids is essential to better understand the tissue‐specific responses of plants to developmental and environmental cues.

Recent ‘omics’ studies have hinted at the potential of integrating metabolomics, lipidomics, transcriptomics and genomics to explain the genetic mechanisms underlying the accumulation of metabolites in response to environmental cues, which could assist DNA marker‐based breeding approaches for stress‐tolerant crop varieties (Cheong *et al.*, 2019, 2020). To support such developments, web‐accessible databases collating ‘omics’ data are indispensable. This is particularly important for information on plant lipids, which still lags behind other ‘omics’ areas. Integration of the plant lipidome with other omics data will be very useful and can open new avenues for plant improvement.

*Arabidopsis thaliana* is the model organism in plant biological research and as such is the best studied (Koornneef and Meinke, 2010). It is at the forefront of plant genetic research and several thousand Arabidopsis mutants are available for nearly all genes involved in plant growth, development and metabolism (Meinke *et al.*, 1998). Thus, in this study, we applied an untargeted approach to analyze the lipidomes of selected Arabidopsis Columbia‐0 tissues, and their changes throughout growth and development. For that, we leveraged the lipid extraction and analysis platform that we recently established (Kehelpannala *et al.*, 2020). We used this data set to generate a public Arabidopsis lipid database representing the distribution of lipids across tissues and through development. Previously, Winter *et al.* (2007) developed an electronic Fluorescent Pictograph (eFP) browser (http://www.bar.utoronto.ca/) to assist the scientific community in analyzing and interpreting microarray data and other large data sets (Winter *et al.*, 2007). This tool has been used to visualize Arabidopsis gene expression data, including different developmental stages, specific tissues, cell types and stress conditions (Kilian *et al.*, 2007; Nakabayashi *et al.*, 2005; Schmid *et al.*, 2005). Today it is widely used and has proven to be a pivotal resource for hypothesis generation in plant science (Brady and Provart, 2009). It has also been used for other species: for example, to show transcriptional changes during *Zea mays* (maize) leaf development in order to facilitate studies into photosynthetic differentiation (Li *et al.*, 2010). More recently, the eFP browser has been used to display RNA sequencing data (Sullivan *et al.*, 2019) from Arabidopsis and from *Fragaria vesca* (wild strawberry) during fruit and flower development (Hawkins *et al.*, 2017). Here, we used a similar approach to visualize the developmental dynamics of lipids in Arabidopsis tissues. We also assembled information on the characteristic fragments observed in MS/MS spectra of the detected lipids, which can be used to develop multiple reaction monitoring (MRM) experiments for the targeted analyses and quantification of specific lipids of interest. We sought to develop a community resource that will enable future research into the functions of lipids in plants.

## RESULTS AND DISCUSSION

### Lipidomics approach

**Figure 1:**
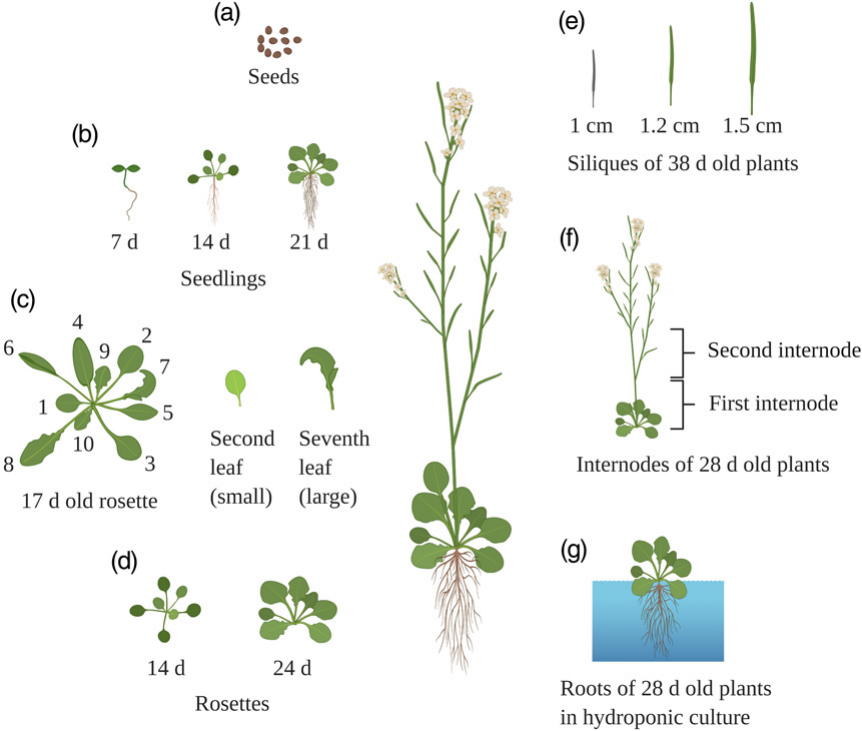
Arabidopsis tissues used for lipid analysis. The tissues analyzed are seeds at 90 days after germination (a), seedlings collected at 7, 14 and 21 days (b), second (small) and seventh (large) rosette leaf of 17‐day‐old plants (c), rosettes collected at 14 and 24 days (d), siliques of 38‐day‐old plants with lengths of 1.0, 1.2 and 1.5 cm (e), first and second internode of 28‐day‐old plants (f) and roots of 28‐day‐old plants (g). The figure was created with BioRender.com.

We created lipid inventories of the model plant Arabidopsis, covering several major tissues and developmental stages. The tissue and developmental stages that we have analyzed include rosette leaves harvested at 14 and 24 days, a small and a large rosette leaf from 17‐day‐old plants, siliques harvested from 38‐day‐old plants (with lengths of 1 cm harvested from the top region of the stem, 1.2 cm from the mid‐region of the stem and 1.5 cm from the lowest region of the stem), the first internode and the second internode from stems of 28‐day‐old plants, whole seedlings at 7, 14 and 21 days, roots from 28‐day‐old plants and seeds collected from plants 90 days after germination (Figure 1).

**Figure 2:**
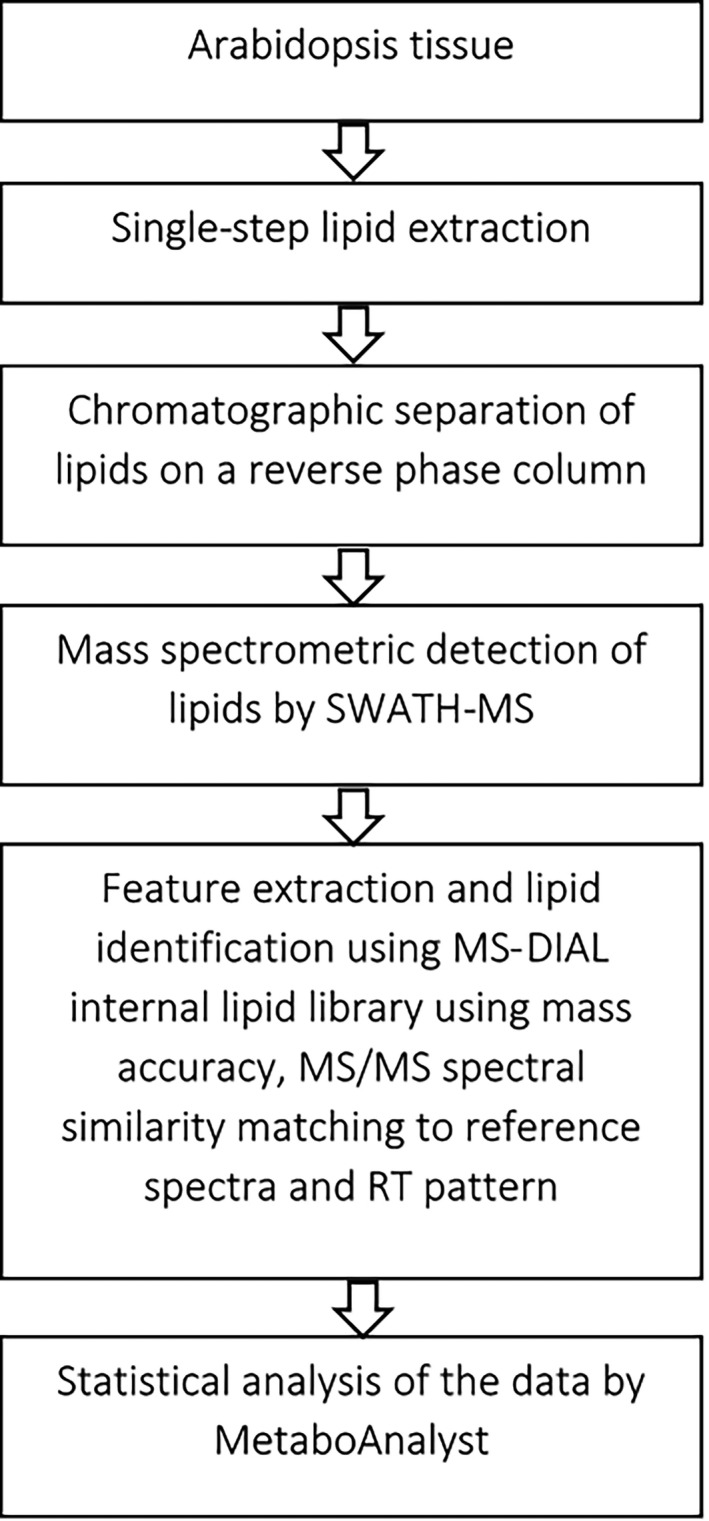
An overview of the untargeted lipid analysis workflow used in this study.

The first crucial step of a lipid analysis is the efficient extraction of lipids from diverse tissues. Recently, we compared four commonly used lipid extraction methods to discern the most efficient and repeatable extraction method for untargeted lipid analysis of Arabidopsis tissues (Kehelpannala *et al.*, 2020). An Agilent 6545‐series mass spectrometer was used to detect lipids after separating them on a reverse‐phase column using an Agilent 1290 high‐performance liquid chromatography (HPLC) system. The lipids were annotated by matching experimental *m*/*z* values with a list of theoretical *m*/*z* values and by retention time alignment with lipid identifications confirmed by MS/MS spectra (Kehelpannala *et al.*, 2020). Our study has shown that the single‐step extraction method published by Shiva *et al.* (2018) is well suited for the untargeted analysis of Arabidopsis lipids and subsequent comparison of tissue‐specific lipid profiles (Kehelpannala *et al.*, 2020). The method described by Shiva *et al.* (2018) is simple, efficient and extracts a broad range of lipids from Arabidopsis leaves, roots, stems, flowers, seedlings, siliques and seeds (Kehelpannala *et al.*, 2020). In the present study we applied a streamlined version of a previously developed pipeline, described by Kehelpannala *et al.* (2020), to extract, detect and identify both known lipids and lipids thus far unreported from Arabidopsis using an untargeted lipidomic approach. We applied the Shiva *et al.* (2018) method with minor modifications to extract lipids from Arabidopsis tissues at different developmental stages, separated the lipids by reverse‐phase column chromatography on an Agilent 1290 HPLC system and used a Sciex TripleTOF^TM^ 6600 QqTOF mass spectrometer to detect lipids. Subsequently all identified lipids were confirmed by MS/MS spectra analysis (Figure 2).

The lipid separation on a reverse‐phase column was achieved as described by Hu *et al.* (2008). This chromatographic separation system is reported to be comparable or better than most others found in the literature, and can profile over 160 lipids belonging to eight different lipid classes from human and mouse plasma in a single run (Hu *et al.*, 2008). To profile lipids in Arabidopsis tissues, we applied a sequential window acquisition of all theoretical mass spectra (SWATH‐MS) method on the Sciex TripleTOF 6600 system and processed the data with the mass spectrometry data‐independent analysis software ms‐dial (Tsugawa *et al.*, 2018). SWATH is a data‐independent acquisition (DIA) method where MS/MS information is systematically obtained for all ionized compounds within a given mass range in an unbiased fashion (Tsugawa *et al.*, 2018). A consecutive series of slightly overlapping precursor isolation windows of 15 Da width within the mass range of 300–1700 *m*/*z* was used in this study. This is advantageous over traditional data‐dependent acquisition (DDA) methods, which do not obtain the product ion spectra of all detectable precursors. In DDA methods, MS/MS spectra of precursor ions are only obtained when a predefined set of criteria is fulfilled, such as falling within a specific number of most abundant ions, present in a precursor ion list or when a defined neutral loss is detected (Zhu *et al.*, 2014).

ms‐dial, developed by Tsugawa *et al.* (2015), has implemented a deconvolution algorithm for data accumulated using DIA methods, which mitigates the risks associated with the SWATH approach, such as convoluted MS/MS spectra, in comparison with DDA (Zhu *et al.*, 2014), and difficulties in the appropriate assignment of the fragment to precursor ions (Tsugawa *et al.*, 2015). It also includes a lipid database using libraries from MassBank (Horai *et al.*, 2010) and LipidBlast (Kind *et al.*, 2013) for the identification of lipids. We used this database to identify lipids in Arabidopsis tissues based on: (i) mass accuracy; (ii) MS/MS spectral similarity matching with the reference spectra in the ms‐dial lipid database; and (iii) retention time pattern of lipids on a C18 reverse‐phase column. ms‐dial contains a database of 61 513 lipids with 135 456 positive and negative ion mode product ion spectra (Tsugawa *et al.*, 2018). To validate the identifications, MS/MS spectra corresponding to each of the lipids identified by ms‐dial were examined individually for characteristic fragmentation patterns and the observed fragments were recorded (Data S1). The retention time pattern of the identified lipids was also considered to confirm lipid identities, as this can diminish false annotations caused by interferents arising from in‐source fragmentation and isobaric/isomeric compounds (Yu *et al.*, 2018). As an example, the ms‐dial library provided 985 lipid identifications out of 21 178 mass spectrometric peaks detected from Arabidopsis rosette tissue. However, a thorough analysis of the identified peaks and fragments confirmed the identification of only 388 lipids (Data S1). We collected detailed information about the lipids identified from each of the seven tissues of Arabidopsis, including lipid class, precursor *m*/*z*, MS/MS fragments and retention time (Data S1). This information can be used to confirm the identities of lipids detected in different Arabidopsis tissues and develop MRM experiments for targeted lipid analysis, which will aid the semi‐ or absolute quantification of these lipids, as described by Yu *et al.* (2018). Depending on the lipids targeted, different strategies and techniques should be applied for the quantification of complex lipids (Khoury *et al.*, 2018; Zullig *et al.*, 2020). The absolute quantification of complex lipids can only be performed if analytical standards are available for each individual lipid molecule (Khoury *et al.*, 2018). Semi‐quantitation (or relative, close or accurate quantification with caution) can be established (Khoury *et al.*, 2018) if at least one internal standard per lipid class is used (https://lipidomics‐standards‐initiative.org/guidelines/lipid‐species‐quantification).

Although many of the major plant lipids were detected in all the Arabidopsis tissues analyzed, several less abundant lipids, which were detected in some Arabidopsis tissues, were not detected in others (Data S1). As a result of the vast structural diversity of lipids, the large concentration range over which lipids are found in biological matrices, and the occurrence of many isomeric and isobaric species, the profiling of lipids using a single analytical method is challenging and quantification is difficult (Khoury *et al.*, 2018). Therefore, to confirm whether these lipids are absent or were simply not detected by our untargeted analysis, targeted experiments must be conducted.

We normalized the peak area of all lipids detected to the equivalent of 100 mg of fresh weight for each sample. The weight‐normalized peak areas of the lipids detected in any Arabidopsis tissue were compared across the developmental stages of the tissue. The accurate and precise measurement of lipids in different biological matrices requires the matrix effects to be characterized and controlled (Panuwet *et al.*, 2016), as co‐eluting matrix components can change the ionization of analytes, resulting in reduced or increased mass spectrometric responses (Panuwet *et al.*, 2016). Therefore, we only compared the changes in levels of individual lipids and lipid classes during the development of each tissue, where the matrix effects will be similar, whereas changes to lipid levels between different tissues were not considered.

We used fold‐change analysis to compare the levels of lipid species and classes during the development of each tissue and the Student’s *t*‐test to identify significant changes. Given the challenges in quantifying a large number of lipids using a single analytical method (Khoury *et al.*, 2018), many untargeted LC‐MS‐based lipidomic studies rely on a comparison of mass spectrometric responses and fold‐change analyses (Acera *et al.*, 2019; Breitkopf *et al.*, 2017; Gil *et al.*, 2018; Liu *et al.*, 2020; Millner *et al.*, 2020; Zhang *et al.*, 2020). Where substantial statistical analyses have been applied, such comparisons of peak intensities (or areas) of detected lipids can provide comprehensive comparisons between biological samples (Wang *et al.*, 2017). For the statistical analysis of the data reported in our study, we used the freely available online software metaboanalyst (www.metaboanalyst.ca/MetaboAnalyst) (Chong and Xia, 2020).

### Lipid nomenclature and abbreviations

A comprehensive classification and nomenclature system is necessary to annotate the detected lipids and communicate them effectively. Here, we followed the updated LIPID MAPS nomenclature, classification and structural representation system (Liebisch *et al.*, 2020) to denote lipids. The lipid classes were abbreviated as follows: ADGGA, acyl diacylglycerol glucuronide; Cer‐AP, ceramide α‐hydroxy fatty acid phytosphingosine; DGs, diacylglycerols; DGDGs, digalactosyldiacylglycerols; Hex‐Cer‐AP, hexosylceramide α‐hydroxy fatty acid phytosphingosine; LPCs, lysophosphatidylcholines; LPEs, lysophosphatidylethanolamines; MGDG, monogalactosyldiacylglycerols; PAs, phosphatidic acids; PCs, phosphatidylcholines; PEs, phosphatidylethanolamines; PGs, phosphatidylglycerols; PIs, phosphatidylinositols; PSs, phosphatidylserines; SQDG, sulfoquinovosyl diacylglycerols; and TGs, triacylglycerols.

The lipid species have been assigned based on the shorthand notation by Liebisch *et al.* (2020), which takes the different levels of structural resolution detected by the mass spectrometer into account (Liebisch *et al.*, 2020). The fatty acid chains in all lipid species reported here are represented by the number of carbon (C) atoms:number of double bonds, e.g. 34:2. The ‘species level’ represents the total number of C atoms and double bonds (Liebisch *et al.*, 2020). For example, the PC species were identified to the species level, e.g. PC 34:2, based on the exact mass measurement in the mass spectrometer and the MS/MS detection of the characteristic fragment ion of *m/z* 184. PE, PG, PI and PS species were also identified to species level, e.g. PE 34:2, PG 34:2, PI 34:2 and PS 34:2, respectively, based on exact mass measurements and the observation of neutral losses of *m/z* 141 in PE species, 189 in PG species, 277 in PI species and 185 in PS species, respectively.

The CL, DG, LPC, LPE and TG species were identified to the ‘molecular species level’, with annotations based on exact mass measurements in the mass spectrometer and the detection of fatty acyl chain‐specific fragments in the MS/MS spectra (Data S1). Molecular species level identification is reached when the constituent fatty acyl/alky chain residues are identified (Liebisch *et al.*, 2020). The separator ‘_’ is used when the *sn*‐position of the acyl/alkyl chains in glycerophospholipids and glycerolipids are unknown (Liebisch *et al.*, 2020). For example, in CL 70:6; 34:2_36:4, the species level is represented by CL 70:6, whereas 34:2_36:4 denotes the sum of acyl residues observed in MS/MS spectra, which correspond to the DG moieties in CL molecules.

Cer‐AP and Hex‐Cer‐AP were identified and presented to the molecular species level: for example, Cer‐AP t34:1 + O; t18:1/16:0 + O and HexCer‐AP t34:1 + O; t18:1/16:0 + O, respectively, where the annotations were based upon the exact mass measurements in the mass spectrometer and the detection of sphingoid base and/or nitrogen (N)‐linked fatty acyl chains in MS/MS spectra (Liebisch *et al.*, 2020). The number of hydroxyl groups in the sphingoid backbone is used to annotate the sphingoid base with ‘d’ denoting ‘di’ and ‘t’ denoting ‘tri’ (Li‐Beisson *et al.*, 2013). The sphingoid backbone is separated by a ‘/’ from the N‐linked fatty acid chain, whereas ‘O’ refers to the keto group in the N‐linked fatty acid chain (Liebisch *et al.*, 2013, 2020).

### Visualizing Arabidopsis tissue‐specific lipid maps

We used an eFP browser to visualize and allow the exploration of the lipids detected in Arabidopsis tissues across development. Visual tools permit a broader audience to access and comprehend large data sets quickly and easily, even without specialized knowledge. Thereby, such tools can encourage research across different disciplines and promote collaborations between scientists in all fields, especially when publicly available (Hawkins *et al.*, 2017). The eFP browser allows the extensive exploration of lipids and their levels in Arabidopsis tissues, whereby lipids of interest for targeted profiling are easily selected.

**Figure 3:**
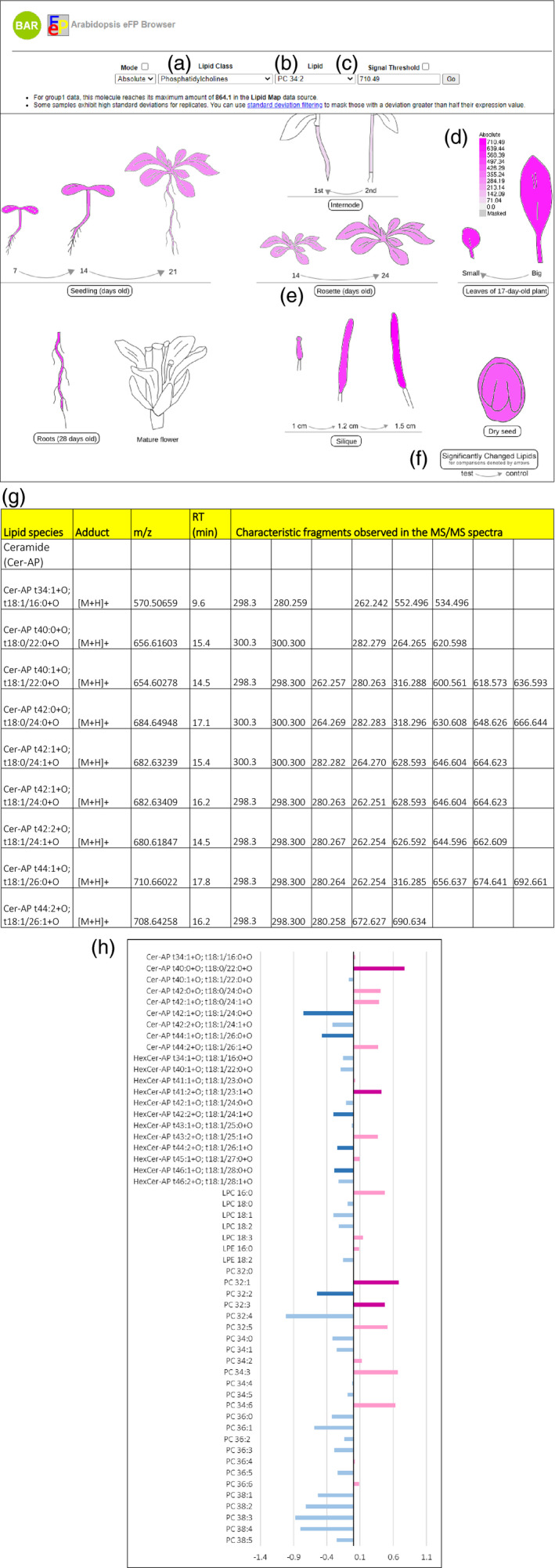
User interface for the electronic Fluorescent Pictograph (eFP) browser, showing an example output for PC 34:2 in Arabidopsis tissues and at different developmental stages. Panels along the top allow the lipid class of interest to be selected from the drop‐down menu (a), the lipid species of interest to be selected from the drop‐down menu (b) and the maximum signal value to be chosen for scaling the colors of the image (c). The scale bar is displayed on the right (d). The total lipid profile of an individual tissue can be accessed by clicking on the button symbolizing a specific tissue, e.g. using the ‘Rosette (days old)’ icon (e). An example eFP browser output showing the first page of the total lipid profile of Arabidopsis rosettes retrieved by clicking on the ‘Rosette (days old)’ icon is given in panel (g). Fold‐change graphs indicating significantly changed levels of lipids can be accessed by clicking on the ‘Significantly Changed Lipids’ icon (f). The growth stages where comparisons were made between lipid levels are given by the arrows. An example eFP browser output showing fold‐change graphs of the lipids identified in rosettes at 14 days compared with 7 days is shown in panel (h).

The Arabidopsis lipid data are displayed for the research community in an eFP browser and can be accessed at http://bar.utoronto.ca/efp_arabidopsis_lipid/cgi‐bin/efpWeb.cgi. This open‐access tool will allow researchers to view lipid levels in different Arabidopsis tissues and developmental stages. It provides comprehensive tissue‐specific lipid profiles from Arabidopsis along with information that can be used for the identification and detection of lipids in subsequent experiments. Users can navigate in the eFP browser by lipid class (Figure 3a) and lipid species (Figure 3b) to locate the lipid species of interest in Arabidopsis tissues and to visualize and compare differences in their levels at different developmental stages of the tissue (Figure 3). Examining the output for PC 34:2 reveals its abundance to different degrees in all tissues analyzed, as indicated by the depth of magenta coloration of the illustrations (Figure 3).

Furthermore, inventories of ADGGA, Cer‐AP, CL, DG, DGDG, HexCer‐AP, MGDG, PC, PE, PG, PI, PS, LPC, LPE, SQDG and TG species in different tissues of Arabidopsis were created and can be retrieved by clicking on the icon describing each tissue (Figure 3). For example, by clicking on the ‘rosettes (days old)’ button (Figure 3e), the total lipid profile of rosettes can be retrieved (Figure 3g). The total lipid profile of each tissue contains information on experimental *m*/*z* values of lipids, experimental *m*/*z* values of mass spectrometric fragments, adducts formed in the mass spectrometer and the experimental retention times, as shown in the example output for Arabidopsis rosettes (Figure 3g). This information is highly valuable for the targeted lipid analysis of specific Arabidopsis tissues. It allows users to easily determine the lipids that they need to analyze by examining the lipid profile of the tissue of interest.

We have further included fold‐change comparisons of lipid levels during the development of seedlings, internodes, rosettes, leaves and siliques, where the users can observe lipids that change significantly during development. Exploring the fold‐change comparison of Arabidopsis rosettes at 24 days compared with 14 days reveals the fluctuating levels of some lipids across development (Figure 3h). Lipids showing an increase in abundance with rosette growth, such as PC 34:2 and PC 34:3, are shown as light‐pink bars, whereas lipids that have decreased in their levels with rosette growth, such as PC 36:1 and PC 36:2, are highlighted by light‐blue bars. The significant increases in lipid levels are represented by magenta bars and significant decreases are represented by dark‐blue bars (Figure 3h). For example, the PC 32:2 level is significantly lower in 24‐day‐old rosettes compared with 14‐day‐old rosettes, as shown by the dark‐blue bar, whereas the PC 32:1 level is significantly higher in 24‐day‐old rosettes compared with 14 day‐old rosettes, as denoted by the magenta bar.

### The Arabidopsis lipid map shows compositional changes throughout development

Many studies have focused on plant lipids and their functions in plant growth and development. Here, we present a study that adds further evidence to the published literature, while providing a visualization tool for lipid levels in different Arabidopsis tissues across development and a community resource to facilitate further studies into the role of plant lipids. We provide a documentation of a large data set of lipids in Arabidopsis, which confirms previously reported trends and discoveries.

### Levels of storage lipids increase during rosette development, whereas the levels of lipids involved in photosynthesis decrease

**Figure 4:**
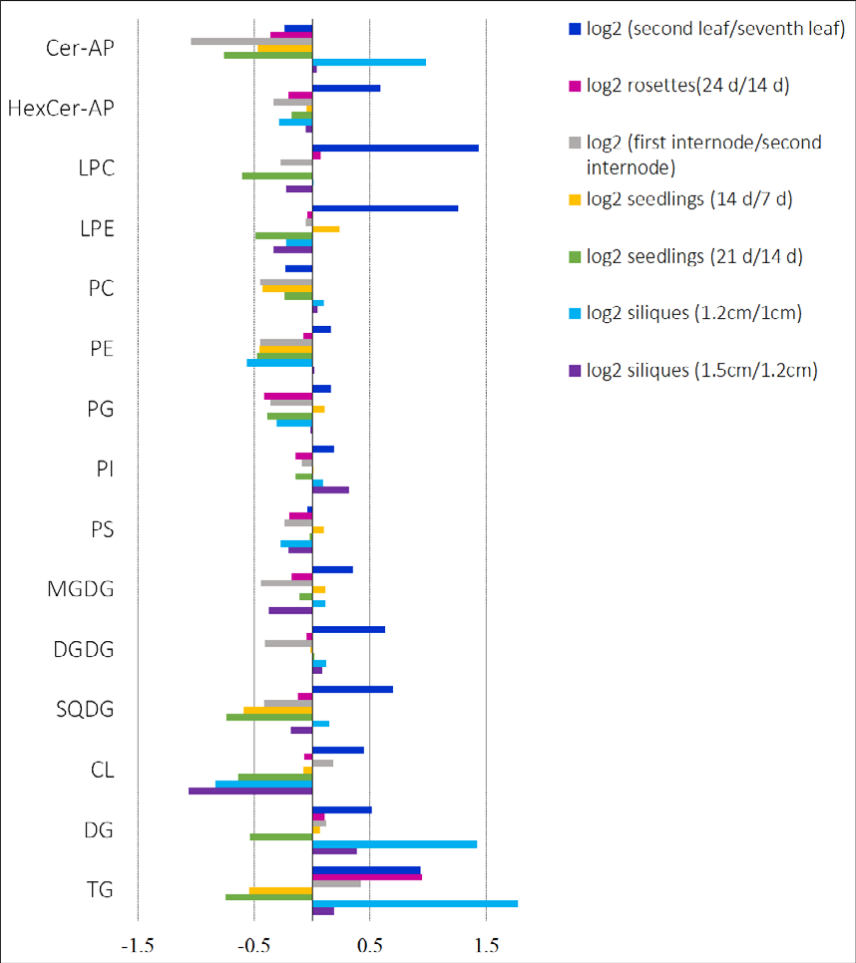
Log2 fold‐change comparison of the levels of lipid classes in Arabidopsis tissues: ceramides (Cer‐AP), hexosylceramides (HexCer‐AP), lysophosphatidylcholines (LPC), lysophosphatidylethanolamines (LPE), phosphatidylcholines (PC), phosphatidylethanolamines (PE), phosphatidylglycerols (PG), phosphatidylinositols (PI), phosphatidylserines (PS), monogalactosyldiacylglycerols (MGDG), digalactosyldiacylglycerols (DGDG), sulfoquinovosyl diacylglycerols (SQDG), cardiolipins (CL), diacylglycerols (DG) and triacylglycerols (TG).

Of the 365 lipids detected in Arabidopsis rosettes, 142 lipid species (39%) showed significant differences in their abundance when 14‐ and 24‐day‐old rosettes were compared (Figure S1). Notably, the levels of CLs, MGDGs and DGDGs were lower in 24‐ compared with 14‐day‐old rosettes, whereas TGs were present at higher levels in 24‐day‐old rosettes (Figure 4). When considering the number of lipids detected, 46% of the MGDGs, 37% of the DGDGs and 50% of the CLs showed significantly lower levels in 24‐day‐old rosettes compared with 14‐day‐old rosettes, whereas 58% of the TGs were present at significantly higher levels in 24‐day‐old rosettes (Figure S1). Our observations agree with previous studies reporting that DGs and TGs, which are the storage lipids in plants (Mumtaz *et al.*, 2020), and ceramide levels increase during leaf development, whereas DGDG, MGDG, PG and SQDG levels decrease (Watanabe *et al.*, 2013). DGDGs, MGDGs, PGs and SQDGs are components of thylakoid membranes and form the photosynthetic complexes (Hölzl and Dörmann, 2019). Interestingly, many of the phospholipids detected in Arabidopsis rosettes, including predominant plant lipids such as PC 34:2, PC 36:4, PE 34:2, PI 34:2, PS 42:2 and PG 34:3, did not show significant differences in their levels in 14‐ and 24‐day‐old rosettes (Figure S1).

### The lipid composition of Arabidopsis leaves is highly specific to their developmental stage

The levels of 347 lipids belonging to 15 lipid classes (Figure S2) were investigated in the second true rosette leaf and the seventh rosette leaf of 17‐day‐old Arabidopsis plants before bolting. The leaves were numbered in the order of their emergence using their morphological characteristics (Figure 1c) (Farmer *et al.*, 2013). The second true leaf is small and spatulate, and is older than the seventh leaf, which is larger in size and narrower (Farmer *et al.*, 2013) (Figure 1c).

Many of the detected ceramides and hexosylceramides were present at significantly higher levels in the smaller and older second leaf compared with the seventh leaf (Figure S2). In contrast, the majority of the detected PCs, PIs, PGs and PSs with acyl chain combinations totalling 36 Cs, and PCs and PEs with 38 Cs, were significantly lower in the second leaf compared with the seventh leaf (Figure S2a–c). Many of the PCs and PEs with 34 Cs did not show significant changes between the two stages (Figure S2a,b), and showed a mixed pattern where the levels of some lipids increased while others decreased. For example, PC 34:6, PE 34:6 and PI 34:6 levels were significantly higher in the second leaf than in the seventh leaf, whereas the opposite was observed for PC 34:1, PE 34:1 and PI 34:1, with levels lower in the second leaf (Figure S2a,b). A clear pattern could not be detected for other saturated and unsaturated phospholipids, with the levels of some increasing and others decreasing (Figure S2a–c). Almost all plant, animal and microbial membranes comprise a blend of saturated and unsaturated fatty acids (Bonaventure *et al.*, 2003). It is believed that this mixture is vital to maintain the physical properties of the plasma membrane, to help plants adapt to environmental perturbations and to prevent phase changes. However, to a certain extent, the exact fatty acid composition of plasma membrane lipids does not seem to be critical (Bonaventure *et al.*, 2003). For example, many Arabidopsis fatty acid mutants are indistinguishable from wild‐type plants when grown under standard conditions (Wallis and Browse, 2002). Nonetheless, major changes in fatty acid composition resulting from environmental stresses such as extreme temperatures can substantially impair plant growth (Bonaventure *et al.*, 2003). Furthermore, Arabidopsis mutant plants containing reduced levels of saturated fatty acids showed growth retardation, highlighting their importance for normal growth. However, the specific function of saturated fatty acids for normal growth remains uncertain (Bonaventure *et al.*, 2003).

The levels of most of the DG, DGDG, MGDG, SQDG and TG species detected were higher in the older second leaf compared with the younger seventh leaf (Figure S2), with all of the total detected SQDGs, 71% of the detected MGDGs, 88% of the detected DGDGs, 80% of the detected DGs and 99% of the detected TGs being more abundant in the second leaf than in the seventh leaf (Figure S2). Of the 347 individual lipid species detected in leaves, 59% were significantly different in their levels when we compared the seventh leaf and second leaf of 17‐day‐old Arabidopsis plants (Figure S2). This demonstrates that the lipid composition of leaves significantly differs depending on the developmental stage and age of a plant. It further highlights the crucial importance of considering the developmental stage when harvesting samples for lipidomic experiments.

### Triacylglycerols (TGs) are the energy reserves in seedlings and are consumed during seedling growth and development

Of the 331 lipids detected in seedlings, 177 lipids showed significant differences in their levels in 7‐ and 14‐day‐old seedlings (Figure S3), whereas 192 lipids showed significant changes in their levels in 14‐ and 21‐day‐old seedlings (Figure S3). The levels of ceramides, PCs, PEs and TGs decreased with seedling growth (Figure 4), and were significantly lower in 21‐day‐old seedlings compared with 14‐day‐old seedlings (Figure 4) and in 14 day‐old seedlings compared with 7‐day‐old seedlings (Figure 4). The decrease in lipid levels, notably TGs, with seedling growth corroborates the observations made in previous studies where a rapid reduction in TG levels as well as the total amount of lipids during seedling growth has been reported (Eastmond *et al.*, 2000; Germain *et al.*, 2001; Kornberg and Beevers, 1957). This rapid decrease in TG levels has been attributed to the peroxisomal β‐oxidation of fatty acids from 45 TGs (Germain *et al.*, 2001). The acetyl‐CoA generated through the β‐oxidation of fatty acids is converted to sucrose in the glyoxylate cycle and gluconeogenesis. Sucrose is then transported throughout the seedlings, which facilitates seedling establishment, and allows growth and development (Eastmond *et al.*, 2000; Kornberg and Beevers, 1957). Our results add further evidence that TGs are likely to act as energy reserves, which are consumed during seedling establishment and growth.

### Lipid levels in Arabidopsis internodes may increase with plant growth but the changes are not significant

The levels of most of the lipids detected in the first internode were either significantly lower compared with those in the second internode or were not significantly different at all (Figure S4). Highly unsaturated TGs with five to eight double bonds were significantly more abundant in the first internode compared with the second internode, whereas TGs with low unsaturation, possessing one to four double bonds, were significantly less abundant (Figure S4e,f). Of the 264 lipid species detected in stems, only 99 displayed significant differences in their levels in the two internodal regions (Figure S4), whereas 186 lipids were present in lower levels in the first internode compared with the second internode (Figure S4). This could argue that the levels of most of the lipids increase with plant growth, but the changes are not significant.

### Levels of storage lipids increase with silique maturation

**Figure 5:**
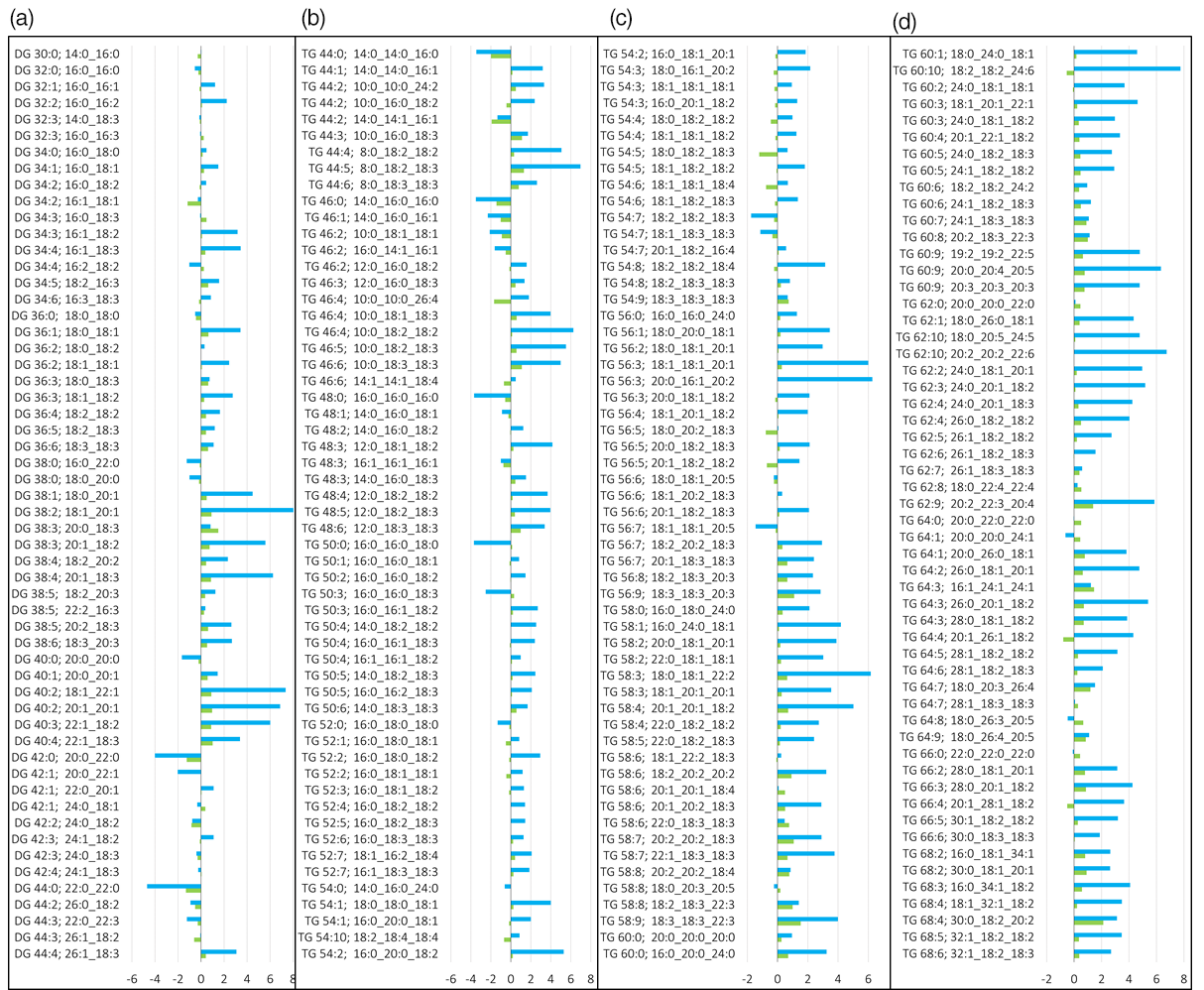
Log2 fold‐change comparison of diacylglycerol (DG) and triacylglycerol (TG) levels in Arabidopsis siliques. Blue bars represent log2 fold‐change comparison of DG and TG in siliques harvested from the mid‐region of the stem (length 1.2 cm) compared with those harvested from the tip of the stem (length 1.0 cm): Log2 1.2/1.0 cm siliques. Green bars represent log2 fold changes of DG and TG in siliques harvested from the base of the stem (length 1.5 cm) compared with those harvested from the mid‐region of the stem (length 1.2 cm): Log2 1.5/1.2 cm siliques.

Siliques harvested from the top, middle and lowest regions of the upper part of stems of 38‐day‐old plants were analyzed. The position of the silique along the stem indicates the developmental stage of an immature silique, with the tip of the stem containing younger siliques and the base containing older siliques (Meinke, 1994, 2020; Meinke *et al.*, 2008). The silique length increases with the number of seeds that it contains: for example, siliques with 60 seeds are known to reach a length of 1.5 cm, whereas siliques with fewer seeds get progressively shorter (Meinke, 1994, 2020; Meinke *et al.*, 2008). Of the 472 lipids detected in siliques, 192 showed significant differences between the siliques harvested from the mid and lower regions of the upper part of the stem (Figure S5), whereas 347 lipids showed significant changes between the siliques harvested from the mid and top regions (Figure S5). No clear pattern of changes was observed for ceramides, hexosylceramides, phospholipids, DGDGs, MGDGs and SQDGs, where the levels of some species increased whereas others decreased in the siliques harvested from different regions of the stem (Figure S5). However, the levels of most ceramides, DGDGs and MGDGs were lower in siliques harvested from the top compared with those harvested from the mid‐region (Figure S5). The low levels of chloroplast lipids such as DGDG and MGDG can be linked to lower photosynthetic activity (Wagstaff *et al.*, 2009) in the siliques from the top region of the stem, which were brownish green in color. A clear increase in many of the DG and TG levels was observed in siliques harvested from different regions of the stem, from the tip to the base (Figure 5), thus indicating that the DG and TG levels increase with silique development. Even though there are no reports documenting changes of storage lipid levels across the development of whole siliques from Arabidopsis, it is expected as silique development is concurrent with the development of the seeds within siliques. It is well known that developing seeds of Arabidopsis accumulate TGs, which are subsequently used up during seed germination and seedling establishment (Baud *et al.*, 2002; Focks and Benning, 1998; Ruuska *et al.*, 2002), whereas DGs are the immediate precursors to TGs (Lung and Weselake, 2006).

### Some plastidically synthesized lipids are absent in root tissue

As observed for all tissues in our study, in roots, PCs, PEs and PIs predominantly contained species with fatty acyl chain combinations totalling 32, 34 and 36 Cs, whereas PSs mostly contained fatty acyl combinations with a total of 34–44 Cs. A similar observation has been made earlier in a study by Devaiah *et al.* (2006), where the authors attributed the differences in acyl chain composition in different tissues to the specificity of acyltransferases and desaturases, suggesting that acyl chains are not randomly distributed among lipid species. Many studies have confirmed that PSs in plants contain more very‐long‐chain species than any other class of plant lipids. For example, Murata *et al.* (1984) found that 18 different species of higher plants contain PSs with very‐long‐chain saturated fatty acids (from C20 to C26). Devaiah *et al.* (2006) uncovered that PS species can contain up to 44 Cs in Arabidopsis tissues, whereas PGs contained fatty acyl chain combinations totalling 32, 34 and 36 C species. In our study, PG 32 and PG 34:4 species were not detected in root tissue. The expected absence of these chloroplast lipids in Arabidopsis roots, a tissue that is generally not photosynthetically active, has been previously noted by Devaiah *et al.* (2006). In agreement with these observations, the number of DGDGs and MGDGs that are components of photosynthetic systems (Hölzl and Dörmann, 2019) was also detected in lower numbers in roots than in leaves and whole rosettes (Data S1).

### Lipid composition of Arabidopsis seeds

The TGs are the major storage lipids in plants and are important for seed germination (Kelly *et al.*, 2011), pollen development and sexual reproduction (Yang and Benning, 2018). The immediate precursor of TGs are DGs (Bates and Browse, 2012), which can also act as signaling molecules in plants (Colón‐González and Kazanietz, 2006; Dong *et al.*, 2012; Eichmann and Lass, 2015; Hong *et al.*, 2010; Lee and Assmann, 1991). However, other studies suggest that the signaling role of DGs may have been lost during evolution and that phosphatidic acid (PA) has emerged as the plant’s second messenger (Munnik and Testerink, 2009; Testerink and Munnik, 2005; Wang *et al.*, 2006). Nonetheless, DGs are crucial molecules in many plant signaling pathways, including lipid signaling pathways (Eichmann and Lass, 2015; Wang, 2004), which are involved in plant growth and stress tolerance (Kue Foka *et al.*, 2020).

Previously, 93 TG species have been identified in Arabidopsis seeds using targeted, direct infusion mass spectrometry, and the importance of elongated acyl chains for seed oil content was demonstrated (Li *et al.*, 2014). A spatial analysis of Arabidopsis seed lipids revealed that the embryonic axis tissues of all serial sections through the embryo of the seed are enriched with PC 34:2 and PI 34:2, whereas cotyledons are rich in PC 38: 3 and PI 36:3 (Sturtevant *et al.*, 2017). The influence of seed anatomy, light and plant‐to‐plant variation on Arabidopsis seed oil content has also been well described, including a data set of the fatty acid composition and distribution in Arabidopsis seeds (Li *et al.*, 2006). Comparison of the polar glycerolipid profiles of the seeds of wild‐type and *pldα1* mutant plants of Arabidopsis using a targeted direct‐infusion mass spectrometric approach showed that the levels of PA, LPC and LPE were significantly lower in wild‐type seeds, suggesting that PLDα1 is involved in membrane lipid degradation in seeds (Devaiah *et al.*, 2006). However, DGs and TGs were not analyzed in that study (Devaiah *et al.*, 2006).

In this study, we identified 180 TG and 52 DG species, which we characterized at the level of their acyl components, in addition to phospholipids and galactolipids from Arabidopsis seeds using an untargeted approach (Data S1). Out of the 180 TGs detected, 58 were highly variable in their measurements, with quality control samples showing a coefficient of variation (CV) greater than 50%. Nonetheless, we included them, as their presence in seed samples is confirmed and this information can be used for targeted studies. With the large number of TGs detected in seeds eluting within 10 min, further targeted studies are necessary to confirm the acyl chain compositions of the TG species detected. Glycerolipids in plant cell membranes primarily consist of 16‐C and 18‐C fatty acids (Millar *et al.*, 2000). However, consistent with our observations, seeds are reported to contain structurally diverse fatty acids with chain lengths varying from 8 to 24 Cs (Millar *et al.*, 2000).

### Possible detection of acyl diacylglyceryl glucuronides in Arabidopsis stems, seedlings and roots

An interesting observation that we made while surveying Arabidopsis lipids across different tissues was that we might have detected several ADDGA species in internodes, seedlings and roots, whereas their presence in other tissues was inconclusive. ADDGA species were annotated based on exact mass measurements, the observation of fatty acyl chain‐specific fragments in the MS/MS spectra, e.g. ADGGA 50:2; 16:0_16:0_18:2, and retention time alignment. The MS/MS spectra of the annotated ADDGA species matched with the reference spectra given in the internal lipid library of ms‐dial. However, with the scarcity of information on ADDGA species and their role in plants and other organisms, targeted analyses are necessary to confirm their presence in Arabidopsis tissues. We have recorded the putative ADDGA species observed in Arabidopsis tissues (Data S1). Once their structures are established and the presence of ADDGA in Arabidopsis tissues undoubtedly confirmed by nuclear magnetic resonance spectroscopy (NMR), their roles in plants can be studied.

### The in‐depth analysis and cataloging of lipid profiles across development provides a resource for the functional analysis of plant lipids

The structural diversity of lipid molecules suggests that these differences are important for the specific functions that they perform (Miquel *et al.*, 1993). However, information on the structure–function relationships of lipids is extremely scarce (Wallis and Browse, 2002). Although the particular functions of some lipids, such as PAs, involved in lipid signaling (Wang, 2005), have been identified, the functions of the vast majority remain unknown or speculative (Miquel *et al.*, 1993).

Many studies show that the distribution and abundance of lipids in Arabidopsis tissues and during development is highly diverse, and that lipids have important roles in plant growth. It is difficult to establish the exact reasons for changes across development, and further studies will be required to help assign precise functions to lipids in developmental processes. The Arabidopsis lipid database presented here, providing information on lipids and corresponding changes across development, will accelerate such future studies of the developmental dynamics and functions of plant lipids and provide a valuable resource to the scientific community.

### Future perspectives

The eFP browser is a user‐friendly, convenient tool that allows us to collate and visualize lipids in different tissues of Arabidopsis. In its current version, the eFP browser presents information on the lipid profiles of the major Arabidopsis tissues and the changes in lipid levels across several developmental stages. The eFP browser is expected to be constantly expanded and improved as new knowledge and information emerges from different laboratories around the world. It is designed to provide an initial framework where information on other important plant lipids, such as glycosylinositol phosphorylceramides (GIPCs), oxylipins, PAs, sterols and sterol esters, can be included once data become available. The eFP browser can also be modified to include the lipid profiles of other Arabidopsis tissues and developmental stages, as well as comparative lipid profiles of the wild type and mutants. The lipid changes in Arabidopsis plants subjected to various environmental stress conditions can also be included. The information from our untargeted analysis here will provide a foundation for future targeted studies aiming at the semi‐quantitation of lipids. Once available, the eFP browser can be modified to include those data.

The integration of data sets from different laboratories will require the metadata associated with growth conditions, experimental design and data collection to be standardized (Hannemann *et al.*, 2009). For example, for microarray‐based gene expression studies, the standards for recording and reporting those data are defined in Minimum Information about a Microarray Experiment (MIAME) developed by the Microarray Gene Expression Database Group (Brazma *et al.*, 2001), and the Human Proteome Organization’s Proteomics Standards Initiative has developed guidelines for the minimum information about a proteomics experiment (MIAPE) (Taylor *et al.*, 2007), which provides guidance on reporting proteomic data. A similar standardization of recording and presenting lipid data should be followed to ensure that the data and results can be easily interpreted and independently verified (Fiehn *et al.*, 2007; Liebisch *et al.*, 2019; O’Donnell Valerie *et al.*, 2020). Whereas reproducibility and overall data quality are comprehensively addressed by publications and guidelines for research in the areas of genomics, proteomics and clinical trials, there are very few reports on guidelines for lipidomic data (O’Donnell Valerie *et al.*, 2020). A short set of guidelines for minimal reporting standards for lipidomics and mass spectrometry‐based biomedical research publications has recently been proposed; however, it is not considered to be the final definitive set of guidelines (O’Donnell Valerie *et al.*, 2020). To create guidelines for lipidomic workflows the Lipidomics Standards Initiative has been established (Liebisch *et al.*, 2019), which is working towards developing standards to enhance the comparability of data and the understanding of the functional roles of lipid species (Liebisch *et al.*, 2019). We have followed the current guidelines set by the Lipidomic Standards Initiative (https://lipidomics‐standards‐initiative.org/guidelines), as can be applied in an untargeted plant profiling study. Following these guidelines to obtain and validate data will facilitate the comparison of data across different laboratories and experimental conditions.

## CONCLUSION

Here, we provide the most comprehensive analysis of Arabidopsis lipids from different tissues to date, including both polar and non‐polar lipid species. We have also collected detailed information on lipid fragments and the retention times of all lipid species in a user‐friendly web‐accessible database that can be used for the identification of lipids from Arabidopsis and to design multiple reaction‐monitoring experiments for targeted analyses. The information given in the web browser can be used to advance future research into the Arabidopsis lipidome, which will aid in understanding lipid changes in response to environmental perturbations or genetic mutations.

This study shows that an untargeted LC‐MS analysis can provide in‐depth insight into lipid abundance, simultaneously identifying more than 300 lipids belonging to several different lipid classes in a single run. Even though significant changes throughout development were not observed for some of the predominant lipid species, such changes were detected in many less common species, suggesting that these lipids play important roles in plant growth and development. Further investigations of these minor lipid species can lead to novel insights into the plant lipidome and its response to developmental cues.

## EXPERIMENTAL PROCEDURES

### Plant growth and sample collection

*Arabidopsis thaliana* (L.) Heynh. Columbia‐0 seeds were placed on 42‐mm Jiffy‐7 pellets (Garden City Plastics, https://www.gardencityplastics.com) and vernalized at 4°C for 3 days. Following the cold treatment, trays were placed in a growth chamber under a 16‐h light/8‐h dark regime at 22°C and 50% relative humidity, with a daytime light intensity of 100–120 µE at the plant level. Rosettes at 14 and 24 days, the second and seventh leaf of 17‐day‐old rosettes, three developmental stages of siliques harvested from 38‐day‐old plants (1.0, 1.2 and 1.5 cm in length), the first internode and second internode from stems of 28‐day‐old plants, whole seedlings at 7, 14 and 21 days, and roots from 28‐day‐old plants were harvested 10–11 h after dawn, snap‐frozen in liquid nitrogen and stored at −80°C until lipid extraction. Seedlings were grown on agar plates (Method S1) and a hydroponic system (Tables S1 and S2) described by Conn *et al.* (2013) was adapted to grow Arabidopsis roots Method S2. To obtain seed samples, at 60 days post‐germination the upper parts of plants, including siliques, were covered with paper bags. These plants were kept in the growth chamber under the above‐mentioned conditions and watered for an additional 14 days. At 74 days post‐germination, the bagged plants were moved to a drying room where the plants were allowed to dry for another 14 days at 20°C and 40% relative humidity under continuous darkness. Finally, seeds were obtained from the dry plants at 90 days post‐germination and subsequently subjected to lipid extraction.

### Lipid extraction

Between eight and 12 biological replicates were prepared from each Arabidopsis tissue and growth stage. Lipids were extracted following the protocol published by Shiva *et al.* (2018), with minor modifications. The plant material was homogenized by cryo‐milling (Precellys 24; Bertin Technologies, https://bertin‐technologies.com) with 400 µl of 2‐propanol containing 0.01% butylated hydroxytoluene (BHT) for two consecutive 45‐s intervals, with a 30‐s pause in between, at 6100 rev/min ​and a temperature of −10°C. Next, the samples were incubated at 75°C for 15 min while being gently shaken at 1400 rev/min. Then, they were cooled to room temperature (25°C) and 1.2 ml of a mixture of chloroform (CHCl_3_)/methanol (MeOH)/water (30/41.5/3.5, v/v/v) was added to each sample. The samples were incubated at 25°C for 24 h with constant gentle shaking. Finally, the solvent was separated and the sample was dried in a vacuum concentrator. A quality control sample was prepared by combining 10 µl of each sample extract.

### Lipid analysis by liquid chromatography mass spectrometry (LC‐MS)

The dried lipid extracts were re‐suspended in 200 µl of butanol (BuOH)/MeOH (1:1) with 10 mm ammonium formate and subjected to LC‐MS analysis, as reported by Hu *et al.* (2008). In brief, the lipid extracts were placed in the autosampler set to 12°C and separated by injecting 15‐µl aliquots into an InfinityLab Poroshell 120 EC‐C_18_ 2.1 × 100 mm (2.7‐µm particle size) column (Agilent, https://www.agilent.com), operated at 55°C using an Agilent 1290 HPLC system with a flow rate of 0.26 ml min^−1^. Elution was performed over a 30‐min binary gradient consisting of acetonitrile (ACN)/water (60/40, v/v) and isopropanol (IPP)/ACN (90/10, v/v) both containing 10 mm ammonium formate as eluents A and B, respectively. The gradient used was: 0–1.5 min isocratic elution with 32% B, which was then increased to 45% B from 1.5 to 4.0 min, then to 52% B from 4.0 to 5.0 min, followed by an increase to 58% B from 5.0 to 8.0 min. Next, it was increased to 66% B from 8.0 to 11.0 min, followed by an increase to 70% B from 11.0 to 14.0 min, and an increase to 75% B from 14.0 to 18.0 min. Then, from 18.0 to 21.0 min, B was increased to 97% and B was maintained at 97% from 21.0 to 25.0 min. Finally, solvent B was decreased to 32% from 25.0 to 25.1 min and B was maintained at 32% for another 4.9 min for column re‐equilibration (Hu *et al.*, 2008).

Lipids were analyzed using a Sciex TripleTOF^™^ 6600 QqTOF mass spectrometer equipped with a Turbo V^™^ dual‐ion source [electro‐spray ionization (ESI) and atmospheric pressure chemical ionization (APCI)] and an automated calibrant delivery system (CDS) using Sequential Window Acquisition of All Theoretical Mass Spectra (SWATH‐MS) in positive ion mode (Tsugawa *et al.*, 2018). The parameters were set as follows: MS1 mass range, 100–1700 *m*/*z*; SWATH scan range, 300–1700 *m*/*z*; MS/MS mass range, 100–1700 *m*/*z*; time‐of‐flight (TOF) MS accumulation time, 50.0 ms; TOF MS/MS accumulation time, 10 ms; collision energy, +45 V; collision energy spread, 15 V; precursor window, 15 Da; and cycle time, 1042 ms. The following ESI parameters were used: source temperature, 250°C; curtain gas, 35 psi; gas 1, 25 psi; gas 2, 25 psi; declustering potential, +80 V; and ion spray voltage floating, 5500 V. The instrument was calibrated automatically with the CDS delivering the APCI calibration solution every five samples.

### Data processing

The raw LC‐MS data were converted into an analysis base file (ABF) format using the reifycs file converter (Tsugawa *et al.*, 2015) and processed through the open‐source software ms‐dial (Tsugawa *et al.*, 2015). The parameters were: MS1 tolerance, 0.01 Da; MS2 tolerance, 0.05 Da; retention time, 0–30 min; MS1 mass range, 300–1700 Da; and minimum peak height, 1000 amplitude. The peaks were aligned with a quality control sample with a retention time tolerance of 0.05 min and MS1 tolerance of 0.015 Da. For all other parameters default values for SWATH‐MS or conventional all‐ions method data processing were retained. Lipids were identified using the ms‐dial internal lipid database with an MS1 accurate mass tolerance of 0.01 Da and an MS2 accurate mass tolerance of 0.05 Da (Tsugawa *et al.*, 2015). Only lipids with a CV below 50% in the quality control samples, showing MS/MS spectral similarity to the reference spectra in the ms‐dial internal database and matching the retention‐time pattern of the lipids belonging to a class, were used for fold‐change analysis (Data S1). Some of the lipids identified by matching the MS/MS spectra to the ms‐dial lipid library and eluting at the expected retention time, showed a CV above 50% in quality control samples. These lipids were included in the data set but were not analyzed further (Data S1).

### Statistical analysis

ms‐dial outputs consisting of peak areas of the identified lipids were analyzed using excel (Microsoft, https://www.microsoft.com ). The peak areas of identified lipids were normalized to the fresh weight of each sample, log transformed, auto scaled and statistically analyzed using the freely available online software metaboanalyst (www.metaboanalyst.ca/MetaboAnalyst). Student’s *t*‐tests were conducted on the identified lipids between different developmental stages of the same tissue to test for significant changes using metaboanalyst and adjusted *P* values were obtained with Benjamini–Hochberg false discovery rate (FDR) correction (Chong and Xia, 2020).

### Development of the electronic Fluorescent Pictograph (eFP) browser

We used the code developed for the Arabidopsis eFP browser (Winter *et al.*, 2007; https://doi.org/10.1371/journal.pone.0000718) as the framework for an ‘Arabidopsis Lipid Map eFP Browser’. Briefly, normalized peak area values were data‐based on the BAR server, and appropriate image and XML control files were created. The eFP browser code was modified to accept lipid names, and the color scheme was also modified to help differentiate the lipid browser from other eFP browsers running on the BAR. Further modifications included linking to downloadable tables of lipids exhibiting significantly different levels in the comparisons noted on the eFP image and of total lipid profiles.

## Author Contributions

TR, BE and UR designed and planned the work. CK conducted the experiments and acquired data. CK, TR, BE and UR interpreted data. AP, EE and NP developed the eFP browser. CK, TR, BE, UR, AP and NP wrote the article. TH and DB reviewed the article. All authors read and approved the final version for publication.

## Conflict of Interest

The authors declare that they have no conflicts of interest associated with this work.

## Supporting information

**Figure S1**. Log2 fold‐change comparison of the levels of lipid species in the 24‐day‐old rosettes compared with 14‐day‐old rosettes (Log_2_ 24 days/14 days).
**Figure S2**. Log2 fold‐change comparison of the levels of lipid species in the second leaf of 17‐day‐old plants compared with the seventh leaf (Log_2_ 2nd leaf/7th leaf).
**Figure S3**. Log2 fold‐change comparison of the levels of lipid species in 14‐day‐old seedlings compared with 7‐day‐old seedlings (Log_2_ 14 days/7 days) (purple) and in 21‐day‐old seedlings compared with 14‐day‐old seedlings (Log_2_ 21/14 days) (green).
**Figure S4**. Log2 fold‐change comparison of the levels of lipid species in the first internode compared with the second internode of 28‐day‐old plants (Log_2_ first internode/second internode).
**Figure S5**. Log2 fold‐change comparison of the levels of lipid species in 1.2‐cm‐long siliques compared with 1‐cm‐long siliques (Log_2_ 1.2/1 cm) (blue bars) and in 1.5‐cm‐long siliques compared with 1.2‐cm‐long siliques (Log_2_ 1.5/1.2 cm) (green bars).

**Method S1**. Growth protocol for Arabidopsis seedlings.
**Method S2**. Growth protocol for Arabidopsis roots.
**Table S1**. Recipe for germination medium by Conn *et al.* (2013).
**Table S2**. Recipe for the standard growth solution by Conn *et al.* (2013).

**Data S1**. Lipids identified from each of the Arabidopsis tissues analyzed and the experimental data used in the study.

## Data Availability

All data sets generated for this study are included in the article or supporting information. The Arabidopsis lipid map eFP browser is available at: http://bar.utoronto.ca/efp_arabidopsis_lipid/cgi‐bin/efpWeb.cgi.
