# Supplementary material for: An Arabidopsis lipid map reveals differences between tissues and dynamic changes throughout development
Source: Plant J. 2021 May 24;107(1):287–302. doi: 10.1111/tpj.15278 (PMC8361726; doi:10.1111/tpj.15278)
Supplement: Supplementary file 1 — Figure S1. Log2 fold‐change comparison of the levels of lipid species in the 24‐day‐old rosettes compared with 14‐day‐old rosettes (Log2 24 days/14 days). Figure S2. Log2 fold‐change comparison of the levels of lipid species in the second leaf of 17‐day‐old plants compared with the seventh leaf (Log2 2nd leaf/7th leaf). Figure S3. Log2 fold‐change comparison of the levels of lipid species in 14‐day‐old seedlings compared with 7‐day‐old seedlings (Log2 14 days/7 days) (purple) and in 21‐day‐old seedlings compared with 14‐day‐old seedlings (Log2 21/14 days) (green). Figure S4. Log2 fold‐change comparison of the levels of lipid species in the first internode compared with the second internode of 28‐day‐old plants (Log2 first internode/second internode). Figure S5. Log2 fold‐change comparison of the levels of lipid species in 1.2‐cm‐long siliques compared with 1‐cm‐long siliques (Log2 1.2/1 cm) (blue bars) and in 1.5‐cm‐long siliques compared with 1.2‐cm‐long siliques (Log2 1.5/1.2 cm) (green bars). [file TPJ-107-287-s002.pdf]

Lipids are abbreviated as follows: phosphatidylcholines (PC), phosphatidylethanolamines (PE), phosphatidylglycerols (PG), phosphatidylinositols (PI), phosphatidylserines (PS), phosphatidic acids (PA), lysophosphatidylcholines (LPC), lysophosphatidylethanolamines (LPE), Ceramide alpha-hydroxy fatty acid-phytosphingosine (Cer-AP), Hexosylceramide alpha-hydroxy fatty acid-phytosphingosine (Hex-Cer-AP), digalactosyldiacylglycerols (DGDG), monogalactosyldiacylglycerols (MGDG), sulfoquinovosyldiacylglycerols (SQDG), acyl diacylglycerol glucuronide (ADGGA), diacylglycerols (DG) and triacylglycerols (TG).

Figures S1 (a-f), S2 (a-f) and S4 (a-f): The magenta-coloured bars represent lipids, which show significantly higher lipid levels and while blue colour bars represent lipids, which show significantly lower lipid levels at different growth stages of a tissue. Non-significant differences are shown in light pink or blue. The light pink-coloured bars represent lipids, which are present at higher levels while the light blue bars represent lipids, which are present in lower levels at different growth stages of a tissue.

Figure S3 (a-g): The purple-coloured bars represent lipids, which have changed significantly in 14 d old seedlings compared to 7 d old seedlings. The green-coloured bars represent lipids, which have changed significantly in 21 d old seedlings compared to 14 d old seedlings. Non-significant changes are shown by bars with a dotted pattern.

Figure S5 (a-h): The blue-coloured bars represent lipids, which have changed significantly in 1.2 cm long siliques compared to 1 cm long siliques. The green-coloured bars represent lipids, which have changed significantly in 1.5 cm long siliques compared to 1.2 cm long siliques. Non-significant changes are shown by bars with a dotted pattern.

Figure S1(a-f) Log2 fold change comparison of the levels of lipid species in the 24 d old rosettes compared to 14 d old rosettes (Log_2 24d/14d)

(a)

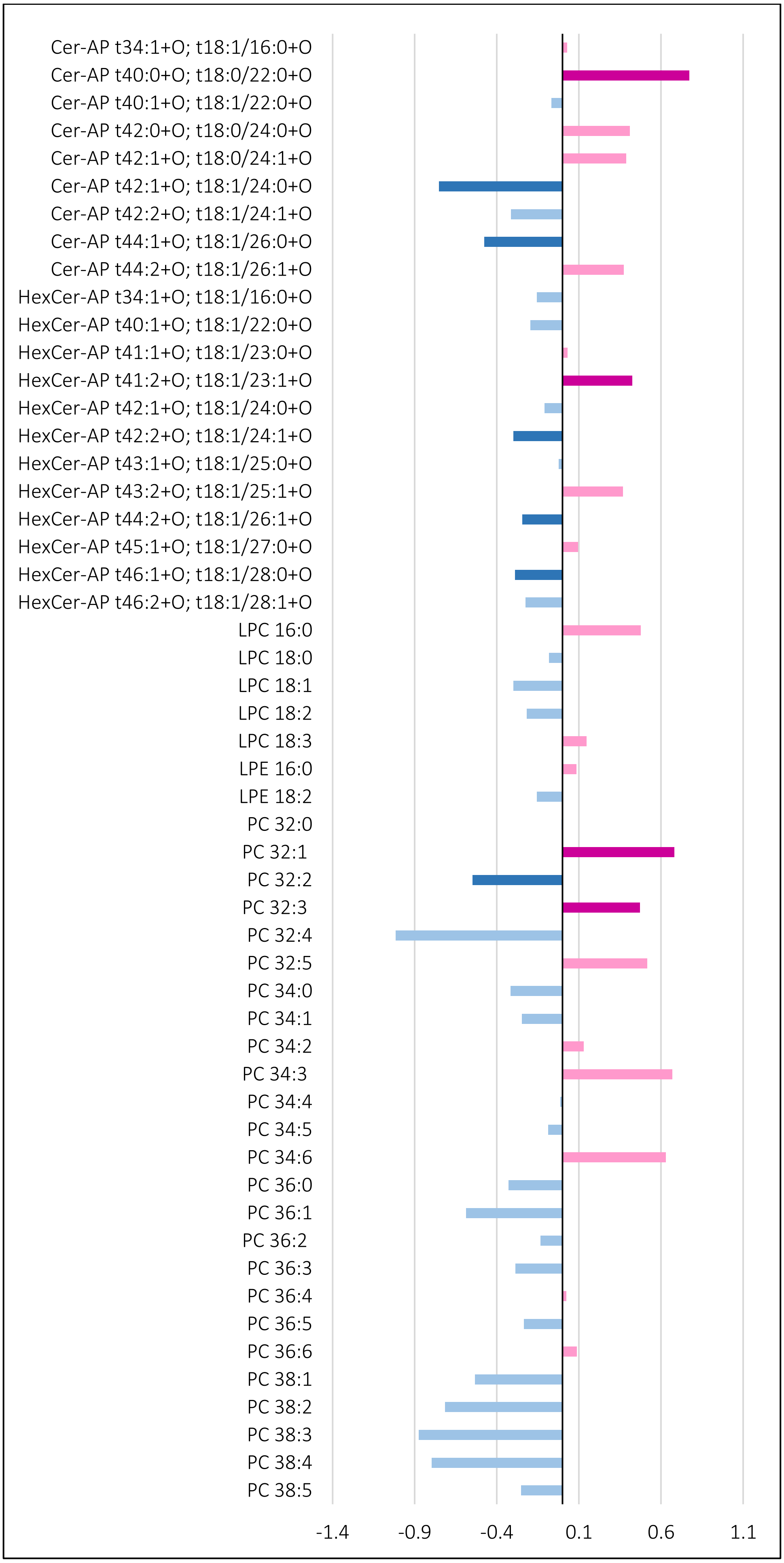

(b)

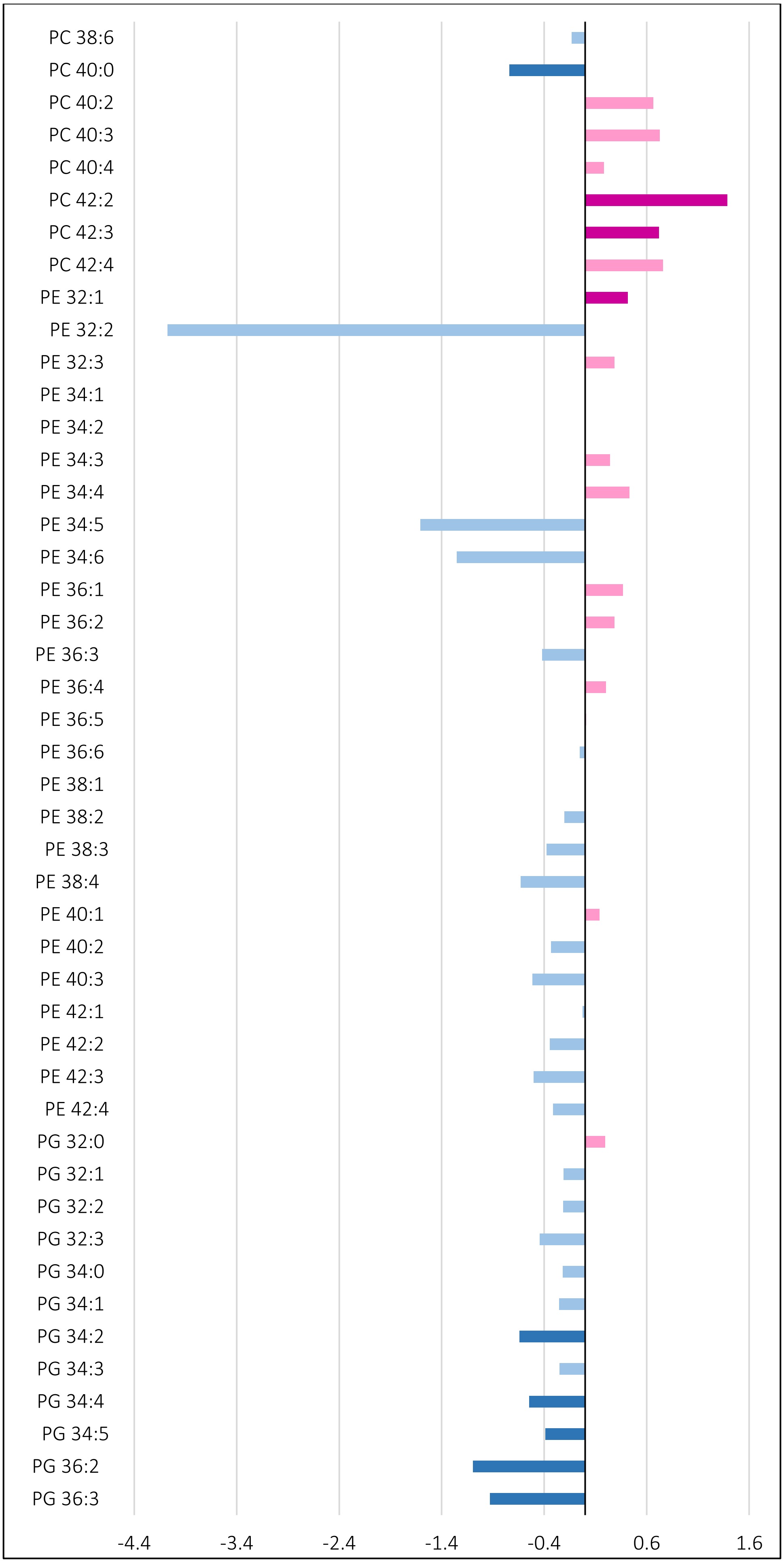
PC 38:6
PC 40:0
PC 40:2
PC 40:3
PC 40:4
PC 42:2
PC 42:3
PC 42:4
PE 32:1
PE 32:2
PE 32:3
PE 34:1
PE 34:2
PE 34:3
PE 34:4
PE 34:5
PE 34:6
PE 36:1
PE 36:2
PE 36:3
PE 36:4
PE 36:5
PE 36:6
PE 38:1
PE 38:2
PE 38:3
PE 38:4
PE 40:1
PE 40:2
PE 40:3
PE 42:1
PE 42:2
PE 42:3
PE 42:4
PG 32:0
PG 32:1
PG 32:2
PG 32:3
PG 34:0
PG 34:1
PG 34:2
PG 34:3
PG 34:4
PG 34:5
PG 36:2
PG 36:3
-4.4
-3.4
-2.4
-1.4
-0.4
0.6
1.6

(c)

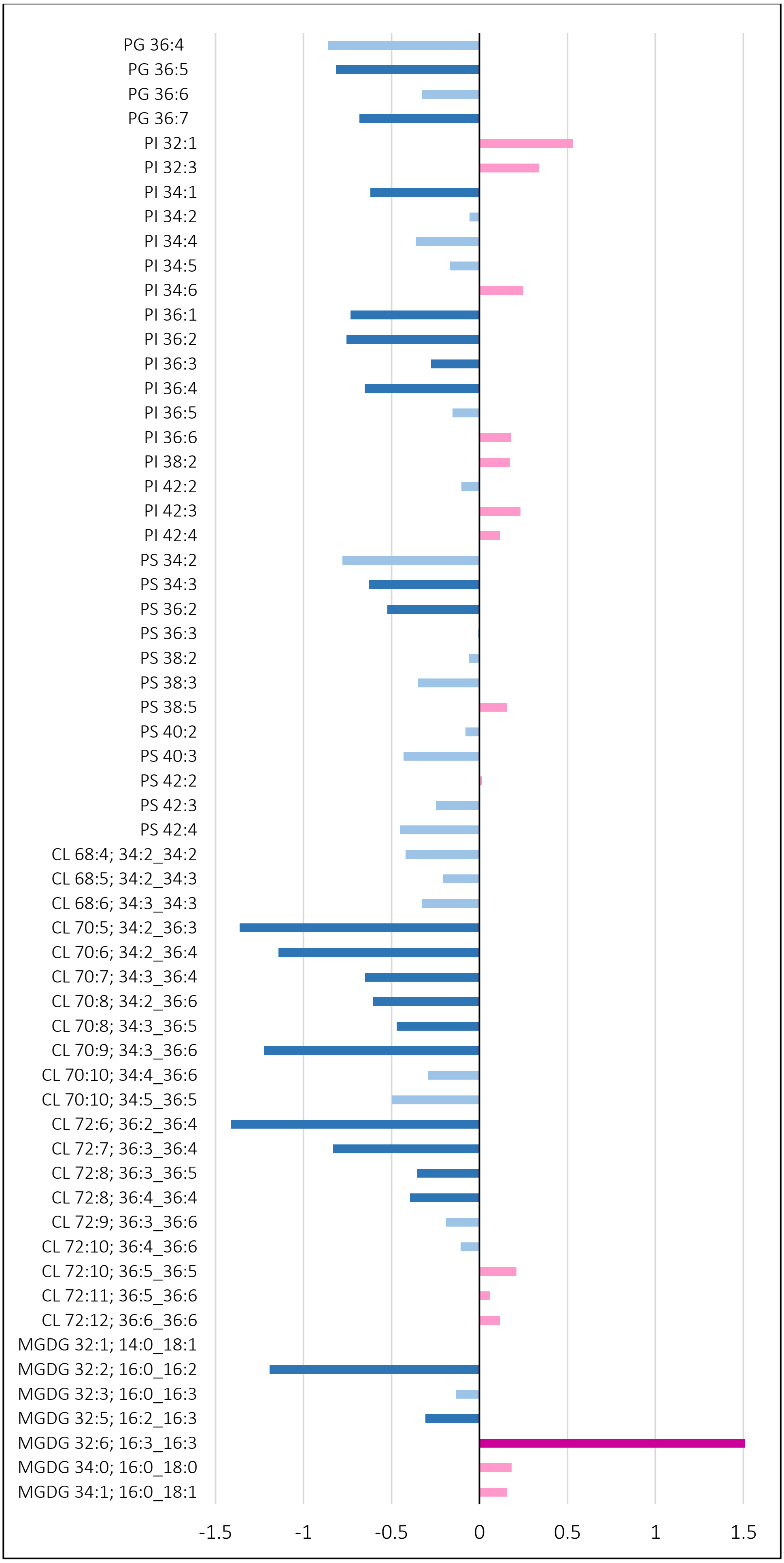

PG 36:4
PG 36:5
PG 36:6
PG 36:7
PI 32:1
PI 32:3
PI 34:1
PI 34:2
PI 34:4
PI 34:5
PI 34:6
PI 36:1
PI 36:2
PI 36:3
PI 36:4
PI 36:5
PI 36:6
PI 38:2
PI 42:2
PI 42:3
PI 42:4
PS 34:2
PS 34:3
PS 36:2
PS 36:3
PS 38:2
PS 38:3
PS 38:5
PS 40:2
PS 40:3
PS 42:2
PS 42:3
PS 42:4
CL 68:4; 34:2_34:2
CL 68:5; 34:2_34:3
CL 68:6; 34:3_34:3
CL 70:5; 34:2_36:3
CL 70:6; 34:2_36:4
CL 70:7; 34:3_36:4
CL 70:8; 34:2_36:6
CL 70:8; 34:3_36:5
CL 70:9; 34:3_36:6
CL 70:10; 34:4_36:6
CL 70:10; 34:5_36:5
CL 72:6; 36:2_36:4
CL 72:7; 36:3_36:4
CL 72:8; 36:3_36:5
CL 72:8; 36:4_36:4
CL 72:9; 36:3_36:6
CL 72:10; 36:4_36:6
CL 72:10; 36:5_36:5
CL 72:11; 36:5_36:6
CL 72:12; 36:6_36:6
MGDG 32:1; 14:0_18:1
MGDG 32:2; 16:0_16:2
MGDG 32:3; 16:0_16:3
MGDG 32:5; 16:2_16:3
MGDG 32:6; 16:3_16:3
MGDG 34:0; 16:0_18:0
MGDG 34:1; 16:0_18:1
-1.5
-1
-0.5
0
0.5
1
1.5

(d)

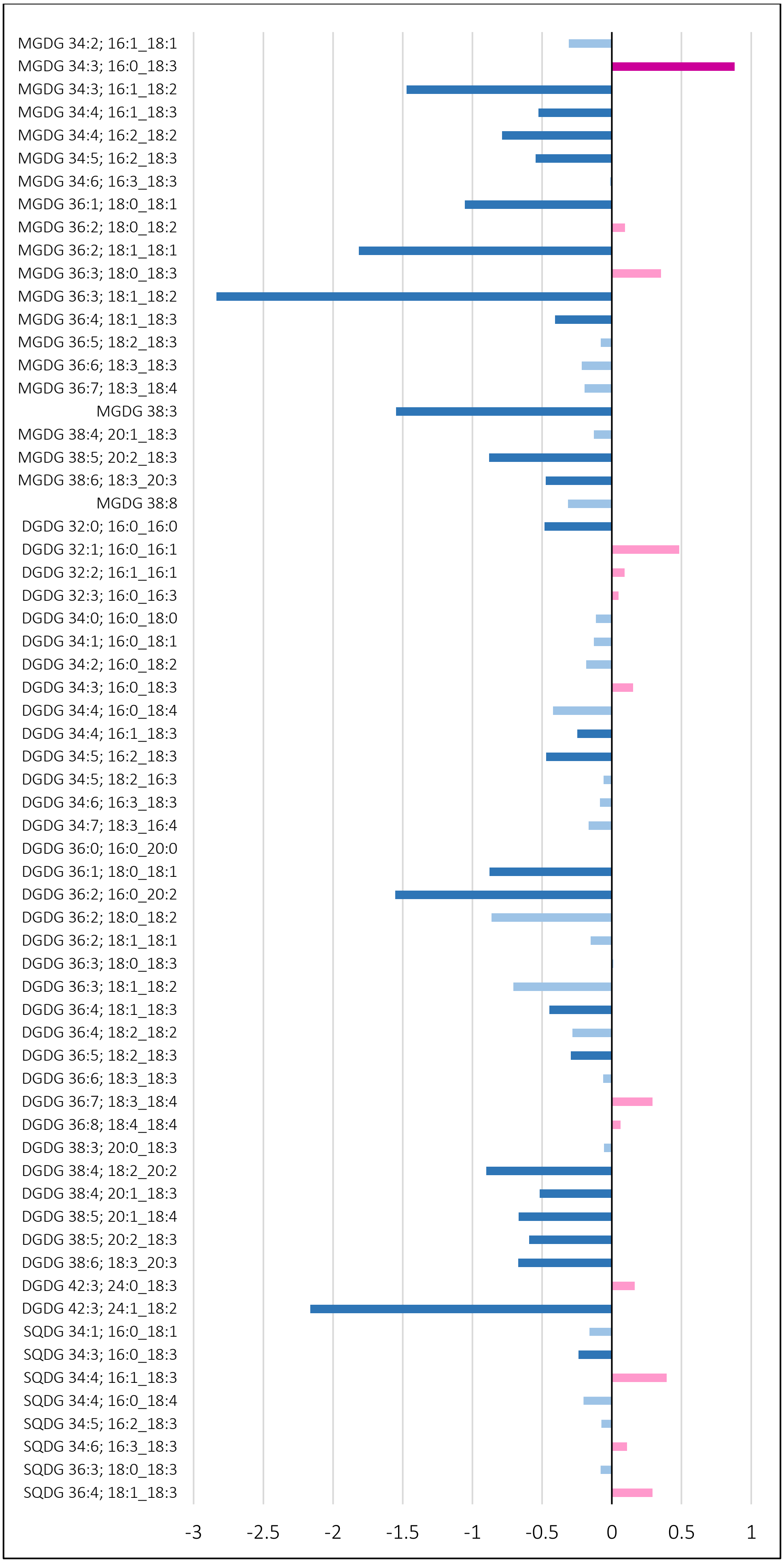

MGDG 34:2; 16:1_18:1
MGDG 34:3; 16:0_18:3
MGDG 34:3; 16:1_18:2
MGDG 34:4; 16:1_18:3
MGDG 34:4; 16:2_18:2
MGDG 34:5; 16:2_18:3
MGDG 34:6; 16:3_18:3
MGDG 36:1; 18:0_18:1
MGDG 36:2; 18:0_18:2
MGDG 36:2; 18:1_18:1
MGDG 36:3; 18:0_18:3
MGDG 36:3; 18:1_18:2
MGDG 36:4; 18:1_18:3
MGDG 36:5; 18:2_18:3
MGDG 36:6; 18:3_18:3
MGDG 36:7; 18:3_18:4
MGDG 38:3
MGDG 38:4; 20:1_18:3
MGDG 38:5; 20:2_18:3
MGDG 38:6; 18:3_20:3
MGDG 38:8
DGDG 32:0; 16:0_16:0
DGDG 32:1; 16:0_16:1
DGDG 32:2; 16:1_16:1
DGDG 32:3; 16:0_16:3
DGDG 34:0; 16:0_18:0
DGDG 34:1; 16:0_18:1
DGDG 34:2; 16:0_18:2
DGDG 34:3; 16:0_18:3
DGDG 34:4; 16:0_18:4
DGDG 34:4; 16:1_18:3
DGDG 34:5; 16:2_18:3
DGDG 34:5; 18:2_16:3
DGDG 34:6; 16:3_18:3
DGDG 34:7; 18:3_16:4
DGDG 36:0; 16:0_20:0
DGDG 36:1; 18:0_18:1
DGDG 36:2; 16:0_20:2
DGDG 36:2; 18:0_18:2
DGDG 36:2; 18:1_18:1
DGDG 36:3; 18:0_18:3
DGDG 36:3; 18:1_18:2
DGDG 36:4; 18:1_18:3
DGDG 36:4; 18:2_18:2
DGDG 36:5; 18:2_18:3
DGDG 36:6; 18:3_18:3
DGDG 36:7; 18:3_18:4
DGDG 36:8; 18:4_18:4
DGDG 38:3; 20:0_18:3
DGDG 38:4; 18:2_20:2
DGDG 38:4; 20:1_18:3
DGDG 38:5; 20:1_18:4
DGDG 38:5; 20:2_18:3
DGDG 38:6; 18:3_20:3
DGDG 42:3; 24:0_18:3
DGDG 42:3; 24:1_18:2
SQDG 34:1; 16:0_18:1
SQDG 34:3; 16:0_18:3
SQDG 34:4; 16:1_18:3
SQDG 34:4; 16:0_18:4
SQDG 34:5; 16:2_18:3
SQDG 34:6; 16:3_18:3
SQDG 36:3; 18:0_18:3
SQDG 36:4; 18:1_18:3
-3
-2.5
-2
-1.5
-1
-0.5
0
0.5
1

(e)

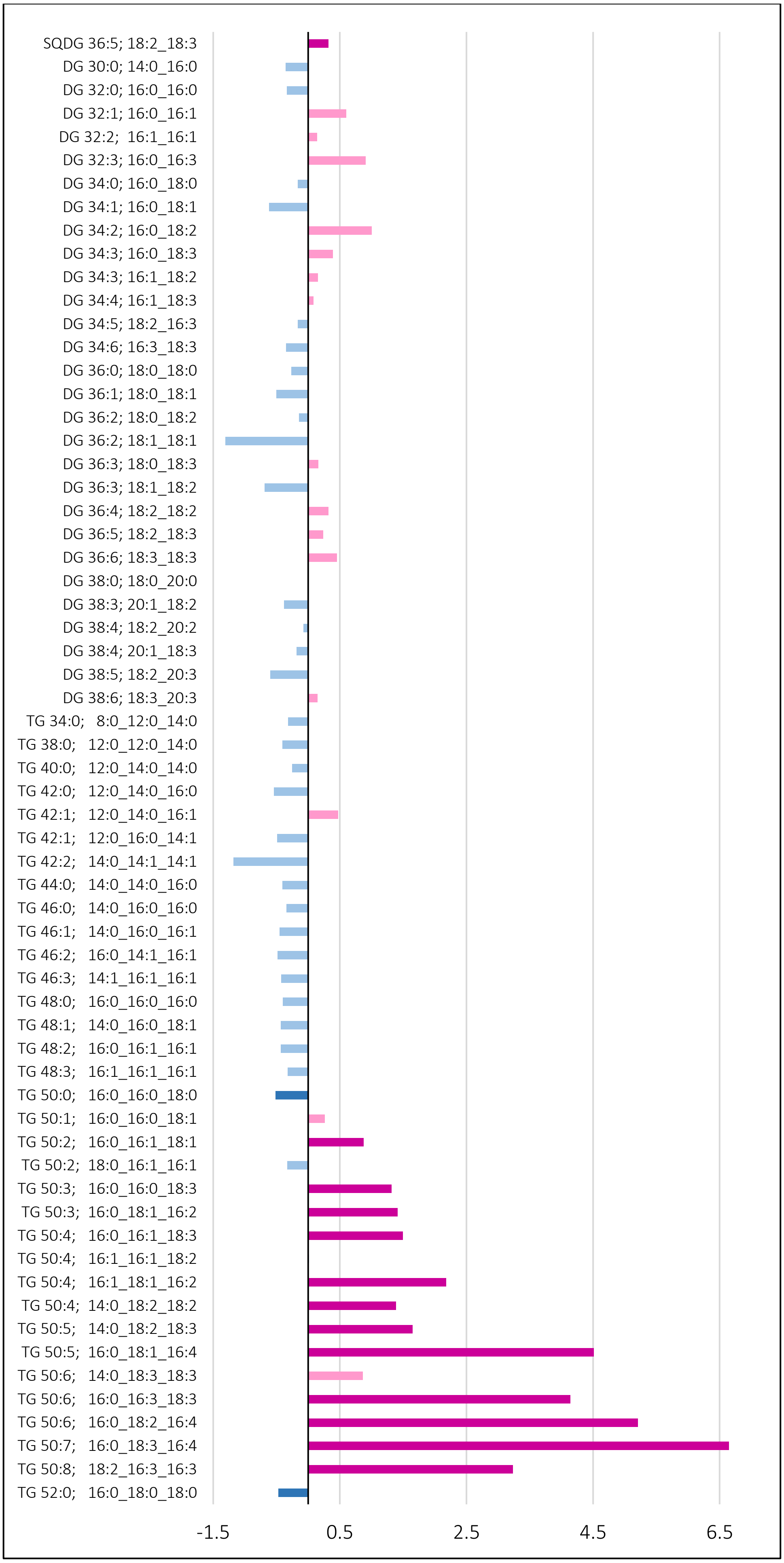

SQDG 36:5; 18:2_18:3
DG 30:0; 14:0_16:0
DG 32:0; 16:0_16:0
DG 32:1; 16:0_16:1
DG 32:2; 16:1_16:1
DG 32:3; 16:0_16:3
DG 34:0; 16:0_18:0
DG 34:1; 16:0_18:1
DG 34:2; 16:0_18:2
DG 34:3; 16:0_18:3
DG 34:3; 16:1_18:2
DG 34:4; 16:1_18:3
DG 34:5; 18:2_16:3
DG 34:6; 16:3_18:3
DG 36:0; 18:0_18:0
DG 36:1; 18:0_18:1
DG 36:2; 18:0_18:2
DG 36:2; 18:1_18:1
DG 36:3; 18:0_18:3
DG 36:3; 18:1_18:2
DG 36:4; 18:2_18:2
DG 36:5; 18:2_18:3
DG 36:6; 18:3_18:3
DG 38:0; 18:0_20:0
DG 38:3; 20:1_18:2
DG 38:4; 18:2_20:2
DG 38:4; 20:1_18:3
DG 38:5; 18:2_20:3
DG 38:6; 18:3_20:3
TG 34:0; 8:0_12:0_14:0
TG 38:0; 12:0_12:0_14:0
TG 40:0; 12:0_14:0_14:0
TG 42:0; 12:0_14:0_16:0
TG 42:1; 12:0_14:0_16:1
TG 42:1; 12:0_16:0_14:1
TG 42:2; 14:0_14:1_14:1
TG 44:0; 14:0_14:0_16:0
TG 46:0; 14:0_16:0_16:0
TG 46:1; 14:0_16:0_16:1
TG 46:2; 16:0_14:1_16:1
TG 46:3; 14:1_16:1_16:1
TG 48:0; 16:0_16:0_16:0
TG 48:1; 14:0_16:0_18:1
TG 48:2; 16:0_16:1_16:1
TG 48:3; 16:1_16:1_16:1
TG 50:0; 16:0_16:0_18:0
TG 50:1; 16:0_16:0_18:1
TG 50:2; 16:0_16:1_18:1
TG 50:2; 18:0_16:1_16:1
TG 50:3; 16:0_16:0_18:3
TG 50:3; 16:0_18:1_16:2
TG 50:4; 16:0_16:1_18:3
TG 50:4; 16:1_16:1_18:2
TG 50:4; 16:1_18:1_16:2
TG 50:4; 14:0_18:2_18:2
TG 50:5; 14:0_18:2_18:3
TG 50:5; 16:0_18:1_16:4
TG 50:6; 14:0_18:3_18:3
TG 50:6; 16:0_16:3_18:3
TG 50:6; 16:0_18:2_16:4
TG 50:7; 16:0_18:3_16:4
TG 50:8; 18:2_16:3_16:3
TG 52:0; 16:0_18:0_18:0
-1.5
0.5
2.5
4.5
6.5

(f)

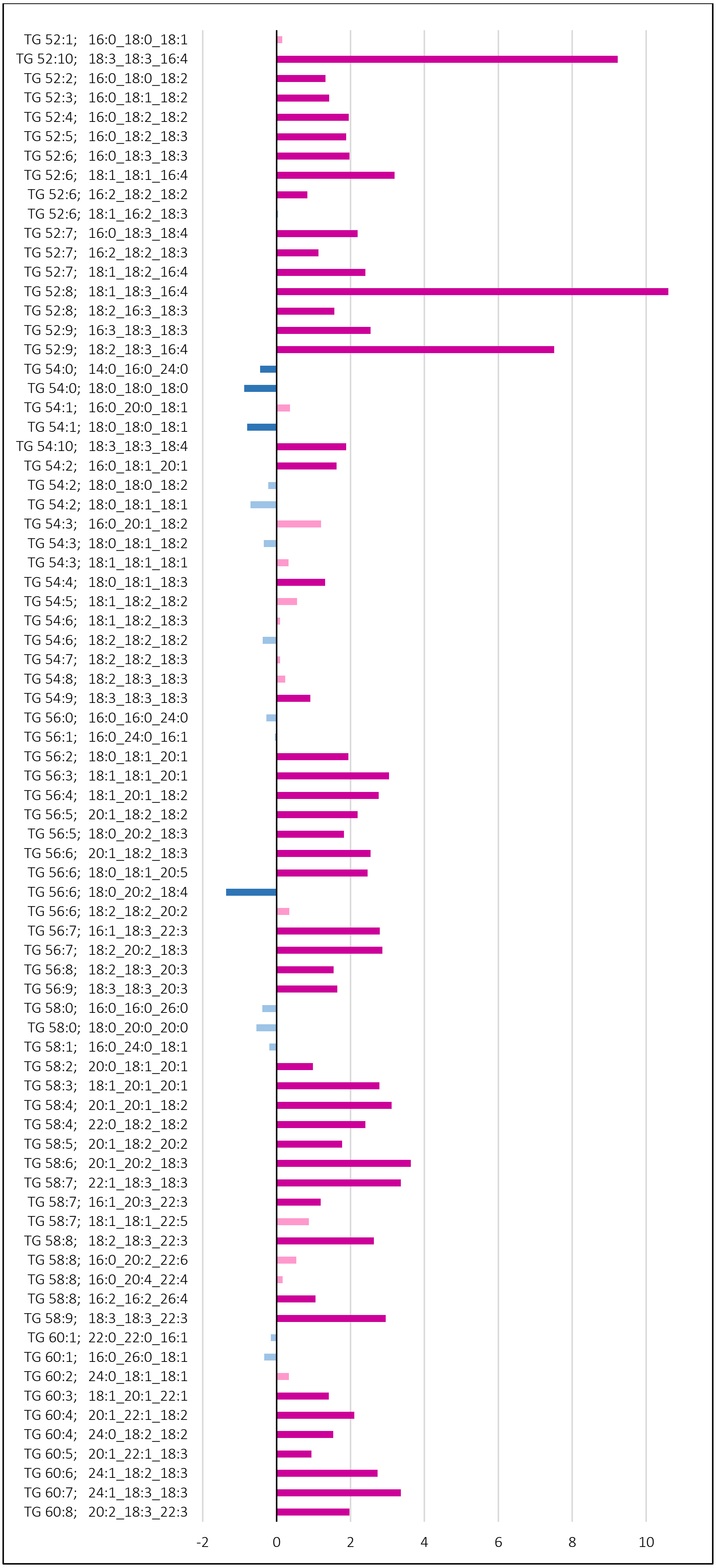

TG 52:1; 16:0_18:0_18:1
TG 52:10; 18:3_18:3_16:4
TG 52:2; 16:0_18:0_18:2
TG 52:3; 16:0_18:1_18:2
TG 52:4; 16:0_18:2_18:2
TG 52:5; 16:0_18:2_18:3
TG 52:6; 16:0_18:3_18:3
TG 52:6; 18:1_18:1_16:4
TG 52:6; 16:2_18:2_18:2
TG 52:6; 18:1_16:2_18:3
TG 52:7; 16:0_18:3_18:4
TG 52:7; 16:2_18:2_18:3
TG 52:7; 18:1_18:2_16:4
TG 52:8; 18:1_18:3_16:4
TG 52:8; 18:2_16:3_18:3
TG 52:9; 16:3_18:3_18:3
TG 52:9; 18:2_18:3_16:4
TG 54:0; 14:0_16:0_24:0
TG 54:0; 18:0_18:0_18:0
TG 54:1; 16:0_20:0_18:1
TG 54:1; 18:0_18:0_18:1
TG 54:10; 18:3_18:3_18:4
TG 54:2; 16:0_18:1_20:1
TG 54:2; 18:0_18:0_18:2
TG 54:2; 18:0_18:1_18:1
TG 54:3; 16:0_20:1_18:2
TG 54:3; 18:0_18:1_18:2
TG 54:3; 18:1_18:1_18:1
TG 54:4; 18:0_18:1_18:3
TG 54:5; 18:1_18:2_18:2
TG 54:6; 18:1_18:2_18:3
TG 54:6; 18:2_18:2_18:2
TG 54:7; 18:2_18:2_18:3
TG 54:8; 18:2_18:3_18:3
TG 54:9; 18:3_18:3_18:3
TG 56:0; 16:0_16:0_24:0
TG 56:1; 16:0_24:0_16:1
TG 56:2; 18:0_18:1_20:1
TG 56:3; 18:1_18:1_20:1
TG 56:4; 18:1_20:1_18:2
TG 56:5; 20:1_18:2_18:2
TG 56:5; 18:0_20:2_18:3
TG 56:6; 20:1_18:2_18:3
TG 56:6; 18:0_18:1_20:5
TG 56:6; 18:0_20:2_18:4
TG 56:6; 18:2_18:2_20:2
TG 56:7; 16:1_18:3_22:3
TG 56:7; 18:2_20:2_18:3
TG 56:8; 18:2_18:3_20:3
TG 56:9; 18:3_18:3_20:3
TG 58:0; 16:0_16:0_26:0
TG 58:0; 18:0_20:0_20:0
TG 58:1; 16:0_24:0_18:1
TG 58:2; 20:0_18:1_20:1
TG 58:3; 18:1_20:1_20:1
TG 58:4; 20:1_20:1_18:2
TG 58:4; 22:0_18:2_18:2
TG 58:5; 20:1_18:2_20:2
TG 58:6; 20:1_20:2_18:3
TG 58:7; 22:1_18:3_18:3
TG 58:7; 16:1_20:3_22:3
TG 58:7; 18:1_18:1_22:5
TG 58:8; 18:2_18:3_22:3
TG 58:8; 16:0_20:2_22:6
TG 58:8; 16:0_20:4_22:4
TG 58:8; 16:2_16:2_26:4
TG 58:9; 18:3_18:3_22:3
TG 60:1; 22:0_22:0_16:1
TG 60:1; 16:0_26:0_18:1
TG 60:2; 24:0_18:1_18:1
TG 60:3; 18:1_20:1_22:1
TG 60:4; 20:1_22:1_18:2
TG 60:4; 24:0_18:2_18:2
TG 60:5; 20:1_22:1_18:3
TG 60:6; 24:1_18:2_18:3
TG 60:7; 24:1_18:3_18:3
TG 60:8; 20:2_18:3_22:3
-2
0
2
4
6
8
10

Figure S2 (a-f) Log2 fold change comparison of the levels of lipid species in the second leaf of 17d old plants compared to the seventh leaf (Log_2 2nd leaf/7th leaf)

(a)

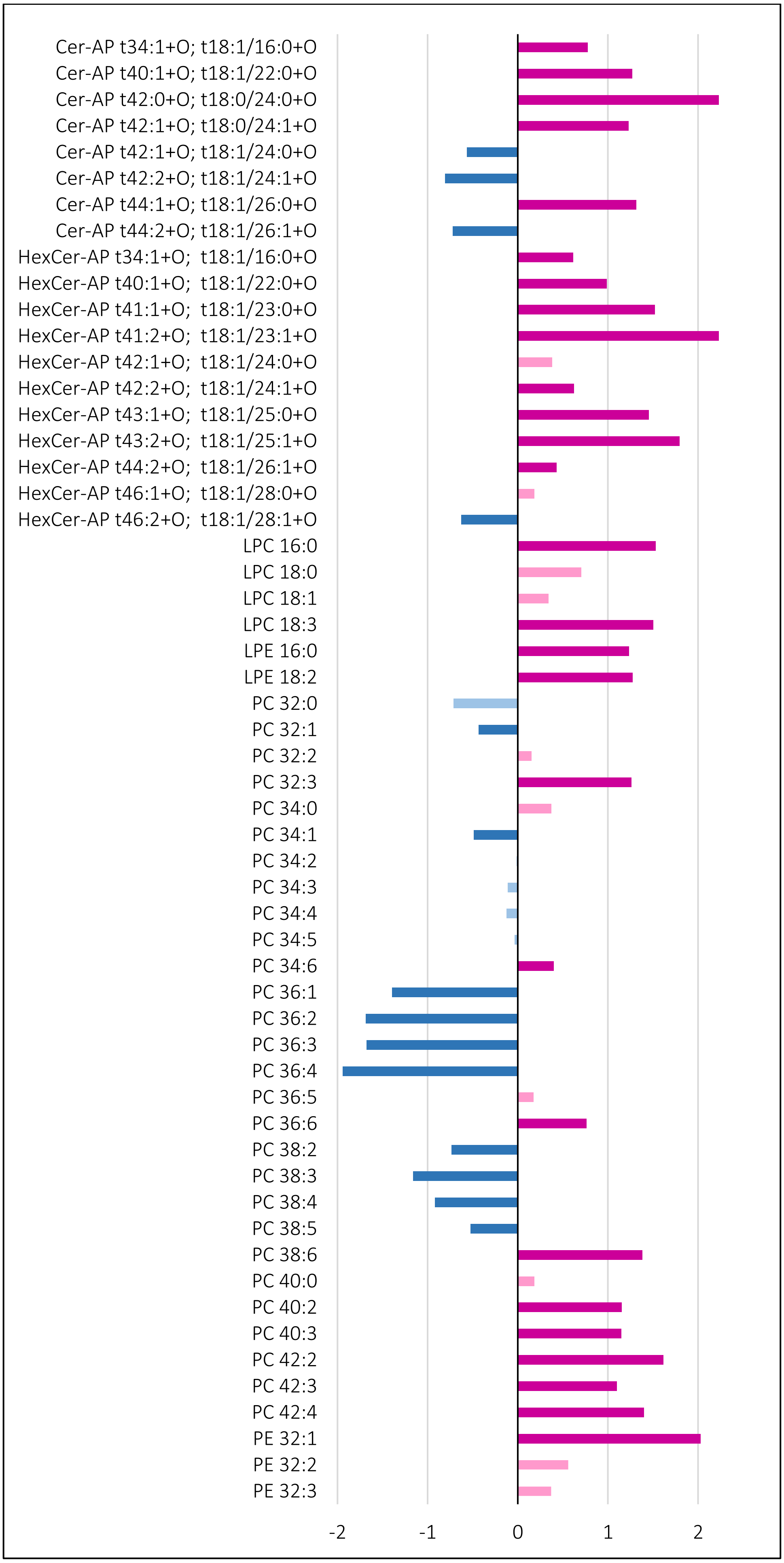

(b)

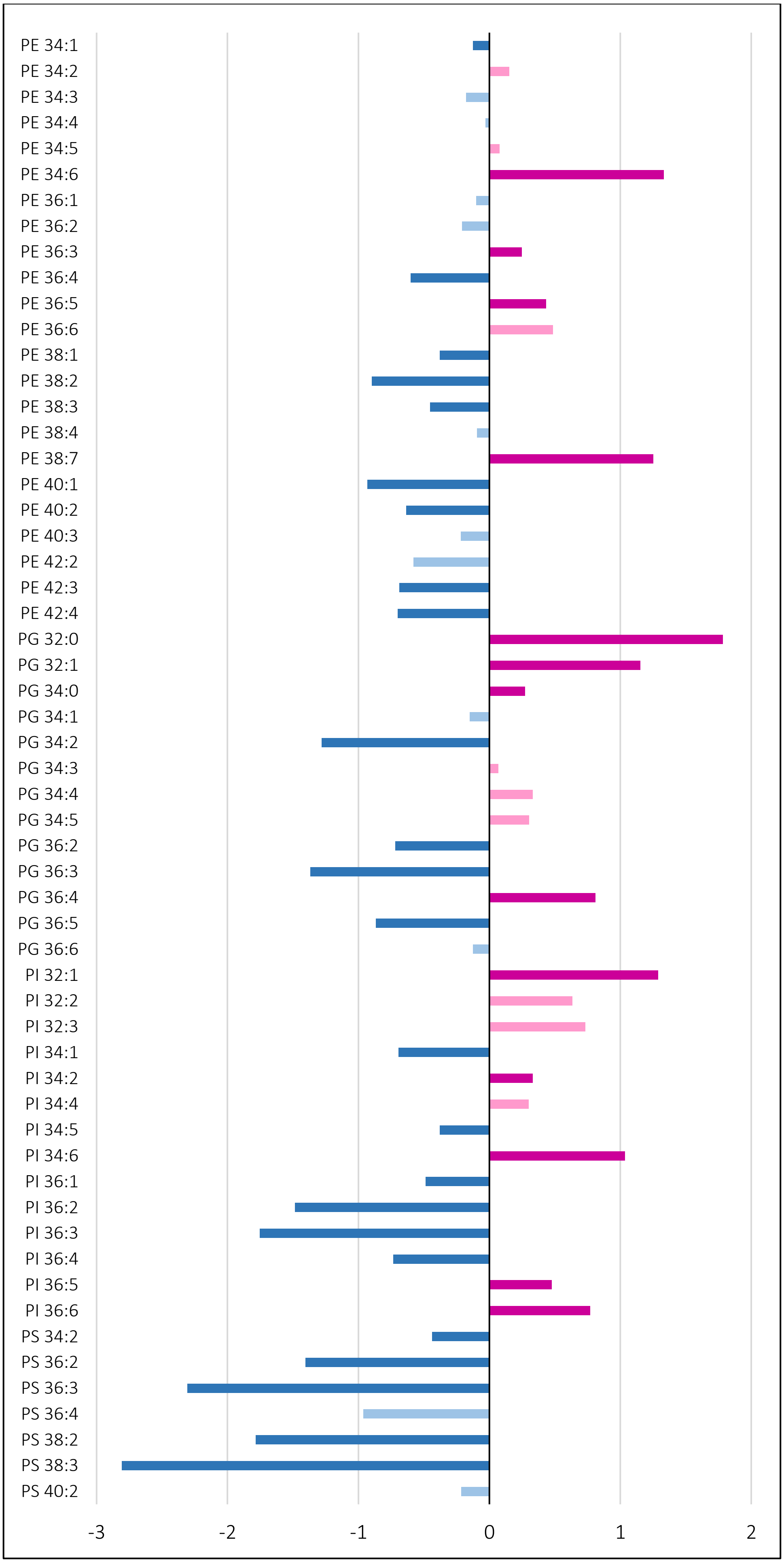

PE 34:1
PE 34:2
PE 34:3
PE 34:4
PE 34:5
PE 34:6
PE 36:1
PE 36:2
PE 36:3
PE 36:4
PE 36:5
PE 36:6
PE 38:1
PE 38:2
PE 38:3
PE 38:4
PE 38:7
PE 40:1
PE 40:2
PE 40:3
PE 42:2
PE 42:3
PE 42:4
PG 32:0
PG 32:1
PG 34:0
PG 34:1
PG 34:2
PG 34:3
PG 34:4
PG 34:5
PG 36:2
PG 36:3
PG 36:4
PG 36:5
PG 36:6
PI 32:1
PI 32:2
PI 32:3
PI 34:1
PI 34:2
PI 34:4
PI 34:5
PI 34:6
PI 36:1
PI 36:2
PI 36:3
PI 36:4
PI 36:5
PI 36:6
PS 34:2
PS 36:2
PS 36:3
PS 36:4
PS 38:2
PS 38:3
PS 40:2
-3
-2
-1
0
1
2

(c)

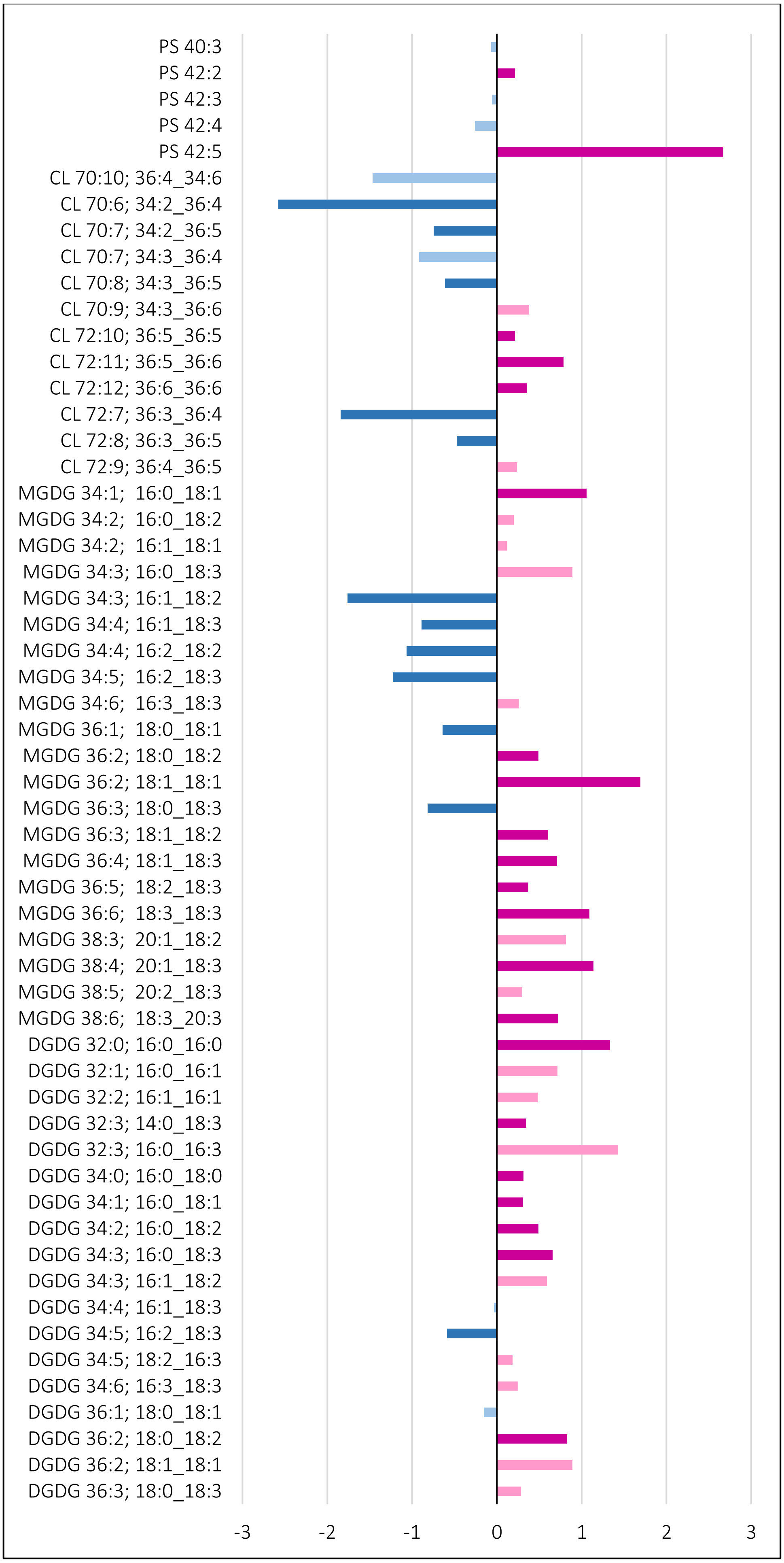
PS 40:3
PS 42:2
PS 42:3
PS 42:4
PS 42:5
CL 70:10; 36:4_34:6
CL 70:6; 34:2_36:4
CL 70:7; 34:2_36:5
CL 70:7; 34:3_36:4
CL 70:8; 34:3_36:5
CL 70:9; 34:3_36:6
CL 72:10; 36:5_36:5
CL 72:11; 36:5_36:6
CL 72:12; 36:6_36:6
CL 72:7; 36:3_36:4
CL 72:8; 36:3_36:5
CL 72:9; 36:4_36:5
MGDG 34:1; 16:0_18:1
MGDG 34:2; 16:0_18:2
MGDG 34:2; 16:1_18:1
MGDG 34:3; 16:0_18:3
MGDG 34:3; 16:1_18:2
MGDG 34:4; 16:1_18:3
MGDG 34:4; 16:2_18:2
MGDG 34:5; 16:2_18:3
MGDG 34:6; 16:3_18:3
MGDG 36:1; 18:0_18:1
MGDG 36:2; 18:0_18:2
MGDG 36:2; 18:1_18:1
MGDG 36:3; 18:0_18:3
MGDG 36:3; 18:1_18:2
MGDG 36:4; 18:1_18:3
MGDG 36:5; 18:2_18:3
MGDG 36:6; 18:3_18:3
MGDG 38:3; 20:1_18:2
MGDG 38:4; 20:1_18:3
MGDG 38:5; 20:2_18:3
MGDG 38:6; 18:3_20:3
DGDG 32:0; 16:0_16:0
DGDG 32:1; 16:0_16:1
DGDG 32:2; 16:1_16:1
DGDG 32:3; 14:0_18:3
DGDG 32:3; 16:0_16:3
DGDG 34:0; 16:0_18:0
DGDG 34:1; 16:0_18:1
DGDG 34:2; 16:0_18:2
DGDG 34:3; 16:0_18:3
DGDG 34:3; 16:1_18:2
DGDG 34:4; 16:1_18:3
DGDG 34:5; 16:2_18:3
DGDG 34:5; 18:2_16:3
DGDG 34:6; 16:3_18:3
DGDG 36:1; 18:0_18:1
DGDG 36:2; 18:0_18:2
DGDG 36:2; 18:1_18:1
DGDG 36:3; 18:0_18:3
-3
-2
-1
0
1
2
3

(d)

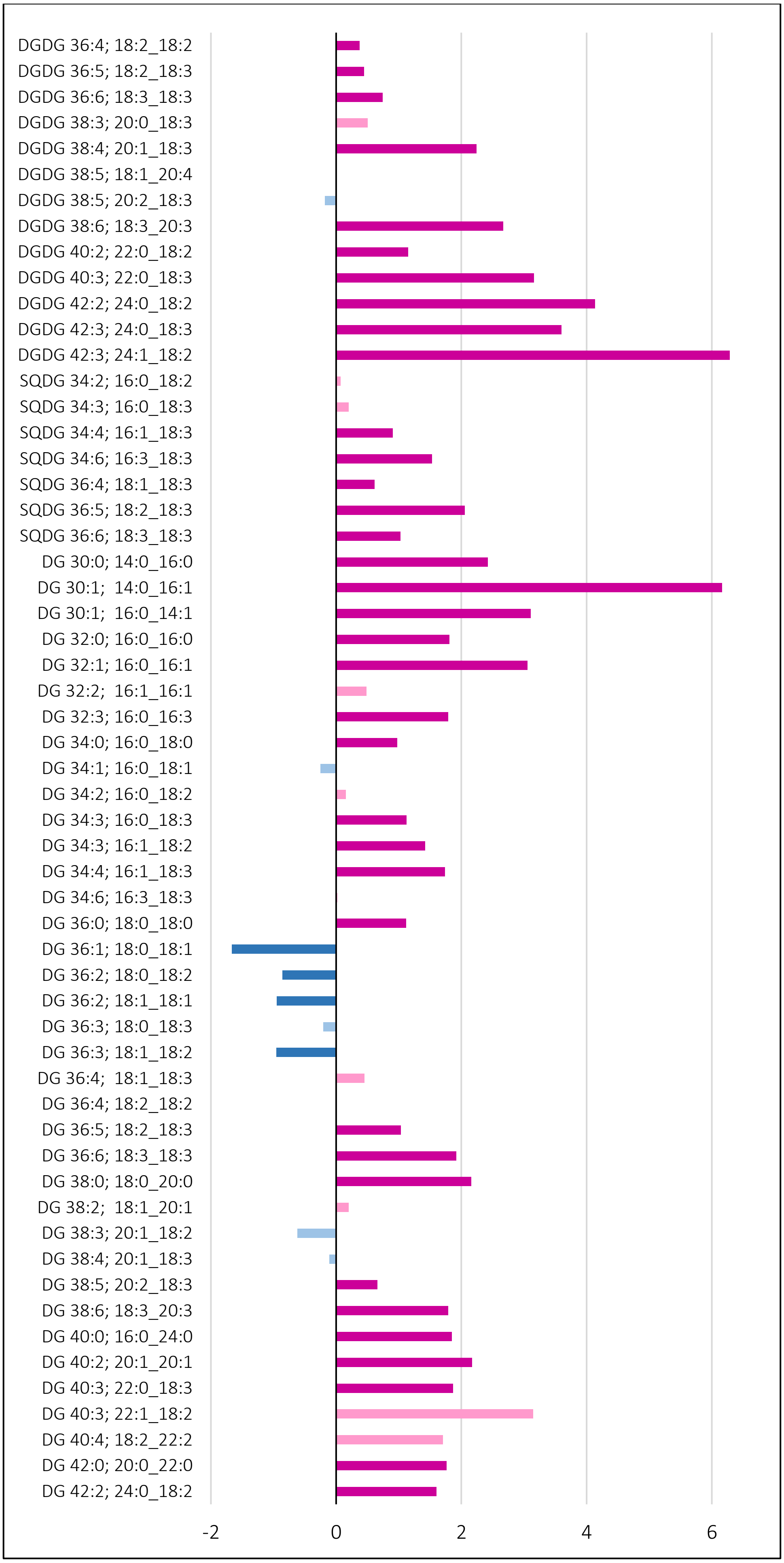

DGDG 36:4; 18:2_18:2
DGDG 36:5; 18:2_18:3
DGDG 36:6; 18:3_18:3
DGDG 38:3; 20:0_18:3
DGDG 38:4; 20:1_18:3
DGDG 38:5; 18:1_20:4
DGDG 38:5; 20:2_18:3
DGDG 38:6; 18:3_20:3
DGDG 40:2; 22:0_18:2
DGDG 40:3; 22:0_18:3
DGDG 42:2; 24:0_18:2
DGDG 42:3; 24:0_18:3
DGDG 42:3; 24:1_18:2
SQDG 34:2; 16:0_18:2
SQDG 34:3; 16:0_18:3
SQDG 34:4; 16:1_18:3
SQDG 34:6; 16:3_18:3
SQDG 36:4; 18:1_18:3
SQDG 36:5; 18:2_18:3
SQDG 36:6; 18:3_18:3
DG 30:0; 14:0_16:0
DG 30:1; 14:0_16:1
DG 30:1; 16:0_14:1
DG 32:0; 16:0_16:0
DG 32:1; 16:0_16:1
DG 32:2; 16:1_16:1
DG 32:3; 16:0_16:3
DG 34:0; 16:0_18:0
DG 34:1; 16:0_18:1
DG 34:2; 16:0_18:2
DG 34:3; 16:0_18:3
DG 34:3; 16:1_18:2
DG 34:4; 16:1_18:3
DG 34:6; 16:3_18:3
DG 36:0; 18:0_18:0
DG 36:1; 18:0_18:1
DG 36:2; 18:0_18:2
DG 36:2; 18:1_18:1
DG 36:3; 18:0_18:3
DG 36:3; 18:1_18:2
DG 36:4; 18:1_18:3
DG 36:4; 18:2_18:2
DG 36:5; 18:2_18:3
DG 36:6; 18:3_18:3
DG 38:0; 18:0_20:0
DG 38:2; 18:1_20:1
DG 38:3; 20:1_18:2
DG 38:4; 20:1_18:3
DG 38:5; 20:2_18:3
DG 38:6; 18:3_20:3
DG 40:0; 16:0_24:0
DG 40:2; 20:1_20:1
DG 40:3; 22:0_18:3
DG 40:3; 22:1_18:2
DG 40:4; 18:2_22:2
DG 42:0; 20:0_22:0
DG 42:2; 24:0_18:2
-2
0
2
4
6

(e)

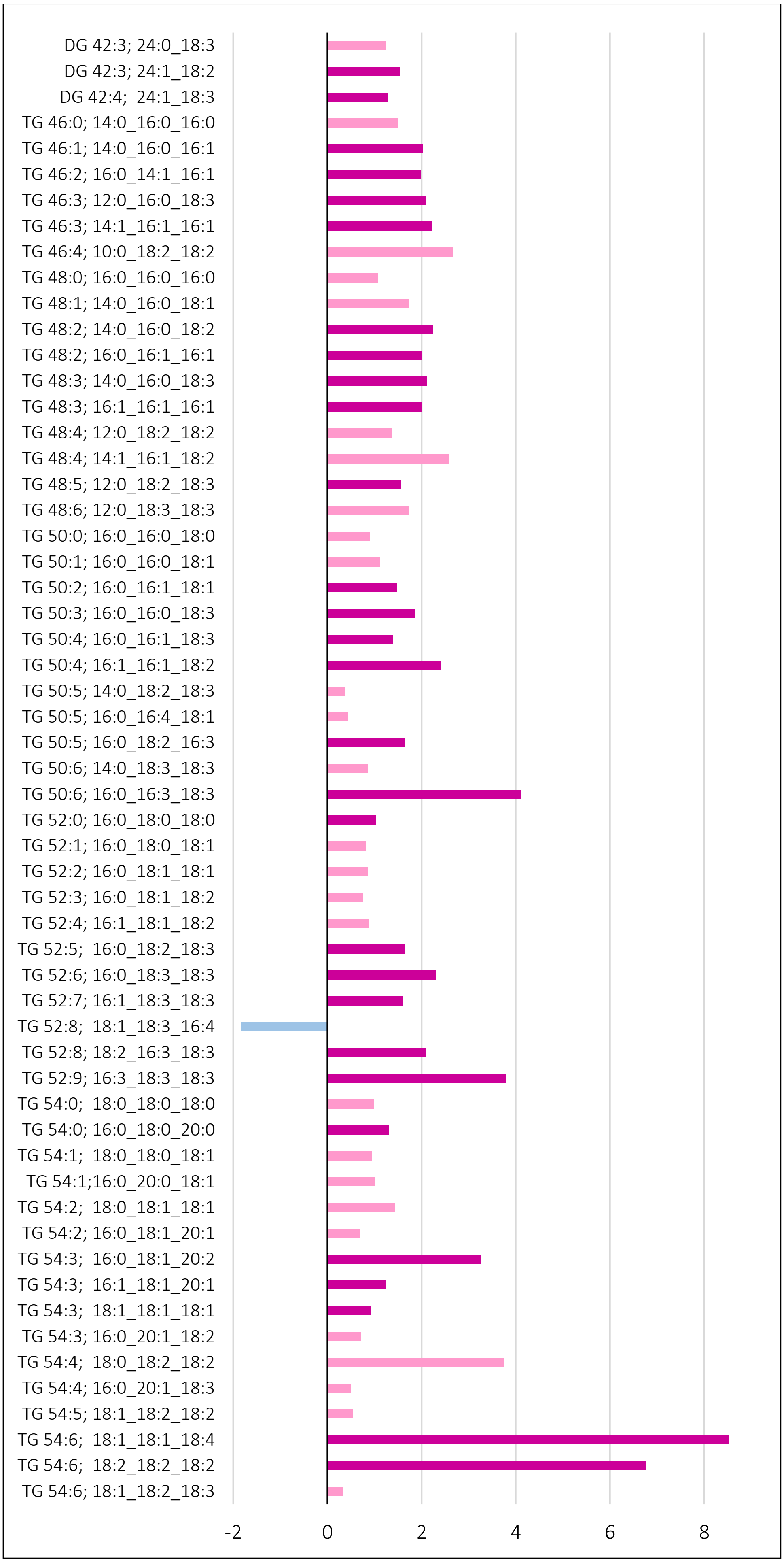
DG 42:3; 24:0_18:3
DG 42:3; 24:1_18:2
DG 42:4; 24:1_18:3
TG 46:0; 14:0_16:0_16:0
TG 46:1; 14:0_16:0_16:1
TG 46:2; 16:0_14:1_16:1
TG 46:3; 12:0_16:0_18:3
TG 46:3; 14:1_16:1_16:1
TG 46:4; 10:0_18:2_18:2
TG 48:0; 16:0_16:0_16:0
TG 48:1; 14:0_16:0_18:1
TG 48:2; 14:0_16:0_18:2
TG 48:2; 16:0_16:1_16:1
TG 48:3; 14:0_16:0_18:3
TG 48:3; 16:1_16:1_16:1
TG 48:4; 12:0_18:2_18:2
TG 48:4; 14:1_16:1_18:2
TG 48:5; 12:0_18:2_18:3
TG 48:6; 12:0_18:3_18:3
TG 50:0; 16:0_16:0_18:0
TG 50:1; 16:0_16:0_18:1
TG 50:2; 16:0_16:1_18:1
TG 50:3; 16:0_16:0_18:3
TG 50:4; 16:0_16:1_18:3
TG 50:4; 16:1_16:1_18:2
TG 50:5; 14:0_18:2_18:3
TG 50:5; 16:0_16:4_18:1
TG 50:5; 16:0_18:2_16:3
TG 50:6; 14:0_18:3_18:3
TG 50:6; 16:0_16:3_18:3
TG 52:0; 16:0_18:0_18:0
TG 52:1; 16:0_18:0_18:1
TG 52:2; 16:0_18:1_18:1
TG 52:3; 16:0_18:1_18:2
TG 52:4; 16:1_18:1_18:2
TG 52:5; 16:0_18:2_18:3
TG 52:6; 16:0_18:3_18:3
TG 52:7; 16:1_18:3_18:3
TG 52:8; 18:1_18:3_16:4
TG 52:8; 18:2_16:3_18:3
TG 52:9; 16:3_18:3_18:3
TG 54:0; 18:0_18:0_18:0
TG 54:0; 16:0_18:0_20:0
TG 54:1; 18:0_18:0_18:1
TG 54:1;16:0_20:0_18:1
TG 54:2; 18:0_18:1_18:1
TG 54:2; 16:0_18:1_20:1
TG 54:3; 16:0_18:1_20:2
TG 54:3; 16:1_18:1_20:1
TG 54:3; 18:1_18:1_18:1
TG 54:3; 16:0_20:1_18:2
TG 54:4; 18:0_18:2_18:2
TG 54:4; 16:0_20:1_18:3
TG 54:5; 18:1_18:2_18:2
TG 54:6; 18:1_18:1_18:4
TG 54:6; 18:2_18:2_18:2
TG 54:6; 18:1_18:2_18:3
-2
0
2
4
6
8

(f)

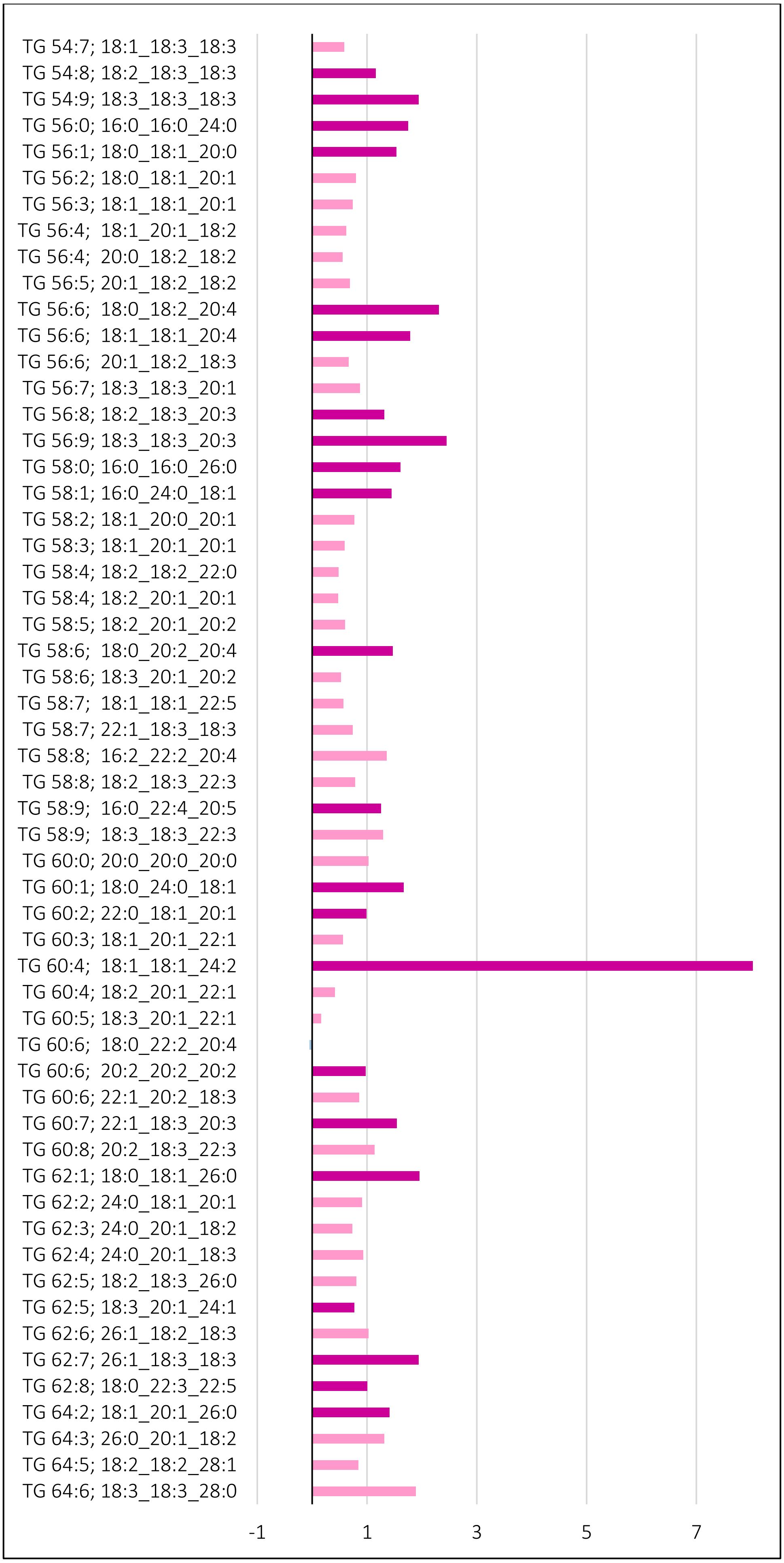

TG 54:7; 18:1_18:3_18:3
TG 54:8; 18:2_18:3_18:3
TG 54:9; 18:3_18:3_18:3
TG 56:0; 16:0_16:0_24:0
TG 56:1; 18:0_18:1_20:0
TG 56:2; 18:0_18:1_20:1
TG 56:3; 18:1_18:1_20:1
TG 56:4; 18:1_20:1_18:2
TG 56:4; 20:0_18:2_18:2
TG 56:5; 20:1_18:2_18:2
TG 56:6; 18:0_18:2_20:4
TG 56:6; 18:1_18:1_20:4
TG 56:6; 20:1_18:2_18:3
TG 56:7; 18:3_18:3_20:1
TG 56:8; 18:2_18:3_20:3
TG 56:9; 18:3_18:3_20:3
TG 58:0; 16:0_16:0_26:0
TG 58:1; 16:0_24:0_18:1
TG 58:2; 18:1_20:0_20:1
TG 58:3; 18:1_20:1_20:1
TG 58:4; 18:2_18:2_22:0
TG 58:4; 18:2_20:1_20:1
TG 58:5; 18:2_20:1_20:2
TG 58:6; 18:0_20:2_20:4
TG 58:6; 18:3_20:1_20:2
TG 58:7; 18:1_18:1_22:5
TG 58:7; 22:1_18:3_18:3
TG 58:8; 16:2_22:2_20:4
TG 58:8; 18:2_18:3_22:3
TG 58:9; 16:0_22:4_20:5
TG 58:9; 18:3_18:3_22:3
TG 60:0; 20:0_20:0_20:0
TG 60:1; 18:0_24:0_18:1
TG 60:2; 22:0_18:1_20:1
TG 60:3; 18:1_20:1_22:1
TG 60:4; 18:1_18:1_24:2
TG 60:4; 18:2_20:1_22:1
TG 60:5; 18:3_20:1_22:1
TG 60:6; 18:0_22:2_20:4
TG 60:6; 20:2_20:2_20:2
TG 60:6; 22:1_20:2_18:3
TG 60:7; 22:1_18:3_20:3
TG 60:8; 20:2_18:3_22:3
TG 62:1; 18:0_18:1_26:0
TG 62:2; 24:0_18:1_20:1
TG 62:3; 24:0_20:1_18:2
TG 62:4; 24:0_20:1_18:3
TG 62:5; 18:2_18:3_26:0
TG 62:5; 18:3_20:1_24:1
TG 62:6; 26:1_18:2_18:3
TG 62:7; 26:1_18:3_18:3
TG 62:8; 18:0_22:3_22:5
TG 64:2; 18:1_20:1_26:0
TG 64:3; 26:0_20:1_18:2
TG 64:5; 18:2_18:2_28:1
TG 64:6; 18:3_18:3_28:0
-1
1
3
5
7

Figure S3 (a-g) Log2 fold change comparison of the levels of lipid species in 14 d old seedlings compared to 7 d old seedlings ($Log_2$14d/7d) (purple) and in 21 d old seedlings compared to 14 d old seedlings ($Log_2$21/14d) (green)

(a)

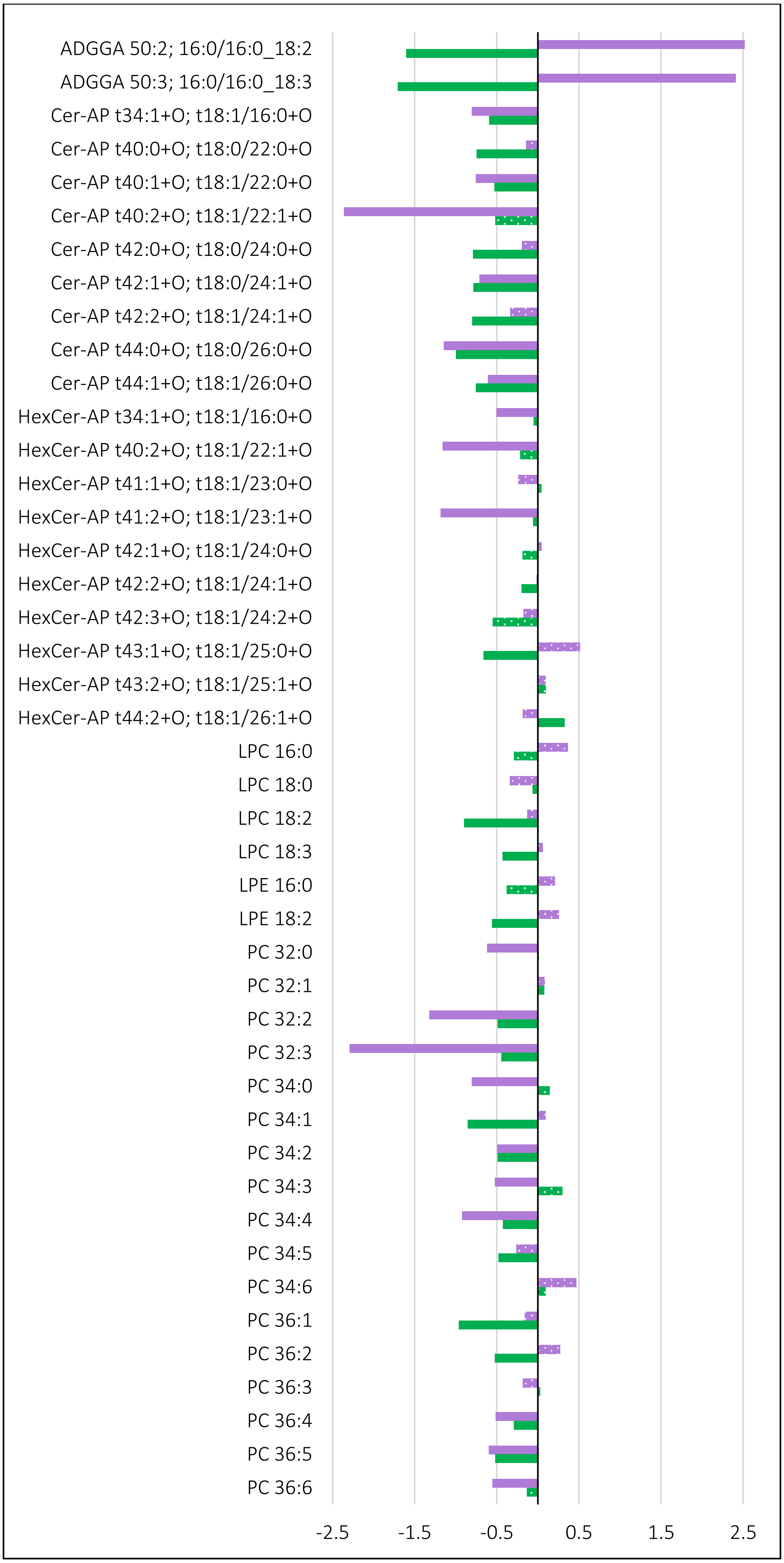

(b)

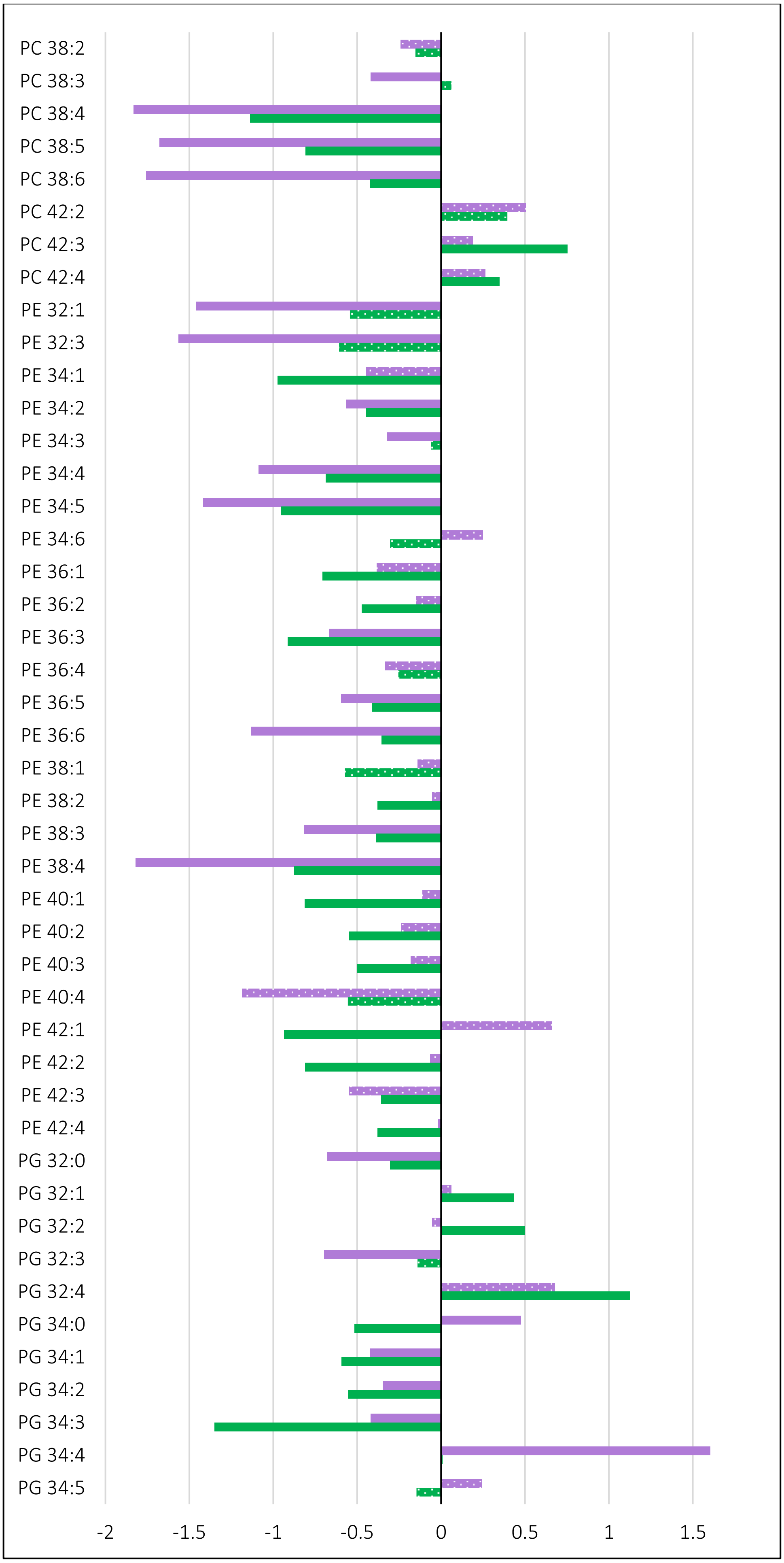

PC 38:2
PC 38:3
PC 38:4
PC 38:5
PC 38:6
PC 42:2
PC 42:3
PC 42:4
PE 32:1
PE 32:3
PE 34:1
PE 34:2
PE 34:3
PE 34:4
PE 34:5
PE 34:6
PE 36:1
PE 36:2
PE 36:3
PE 36:4
PE 36:5
PE 36:6
PE 38:1
PE 38:2
PE 38:3
PE 38:4
PE 40:1
PE 40:2
PE 40:3
PE 40:4
PE 42:1
PE 42:2
PE 42:3
PE 42:4
PG 32:0
PG 32:1
PG 32:2
PG 32:3
PG 32:4
PG 34:0
PG 34:1
PG 34:2
PG 34:3
PG 34:4
PG 34:5
-2
-1.5
-1
-0.5
0
0.5
1
1.5

(c)

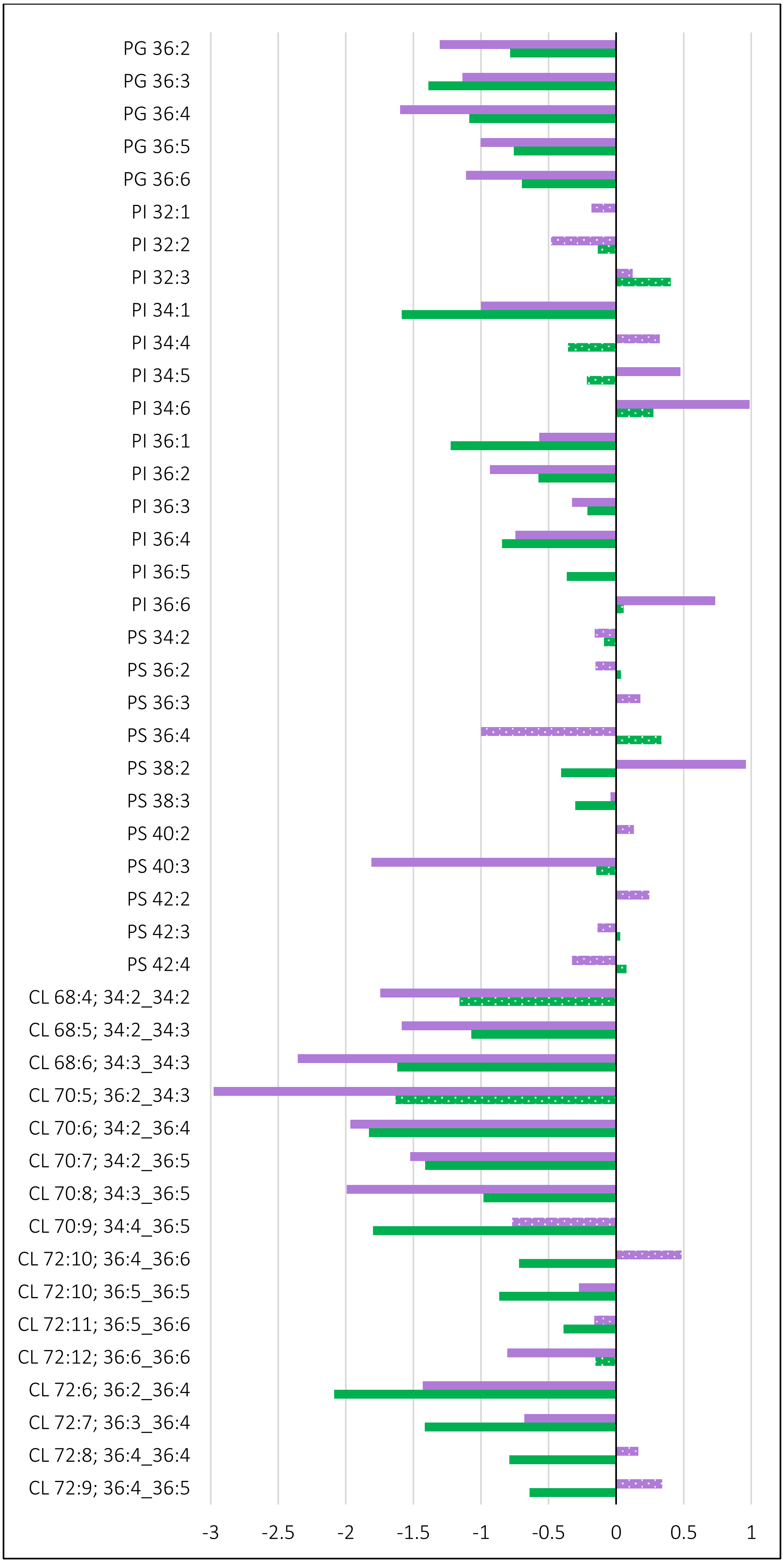
PG 36:2
PG 36:3
PG 36:4
PG 36:5
PG 36:6
PI 32:1
PI 32:2
PI 32:3
PI 34:1
PI 34:4
PI 34:5
PI 34:6
PI 36:1
PI 36:2
PI 36:3
PI 36:4
PI 36:5
PI 36:6
PS 34:2
PS 36:2
PS 36:3
PS 36:4
PS 38:2
PS 38:3
PS 40:2
PS 40:3
PS 42:2
PS 42:3
PS 42:4
CL 68:4; 34:2_34:2
CL 68:5; 34:2_34:3
CL 68:6; 34:3_34:3
CL 70:5; 36:2_34:3
CL 70:6; 34:2_36:4
CL 70:7; 34:2_36:5
CL 70:8; 34:3_36:5
CL 70:9; 34:4_36:5
CL 72:10; 36:4_36:6
CL 72:10; 36:5_36:5
CL 72:11; 36:5_36:6
CL 72:12; 36:6_36:6
CL 72:6; 36:2_36:4
CL 72:7; 36:3_36:4
CL 72:8; 36:4_36:4
CL 72:9; 36:4_36:5
-3
-2.5
-2
-1.5
-1
-0.5
0
0.5
1

(d)

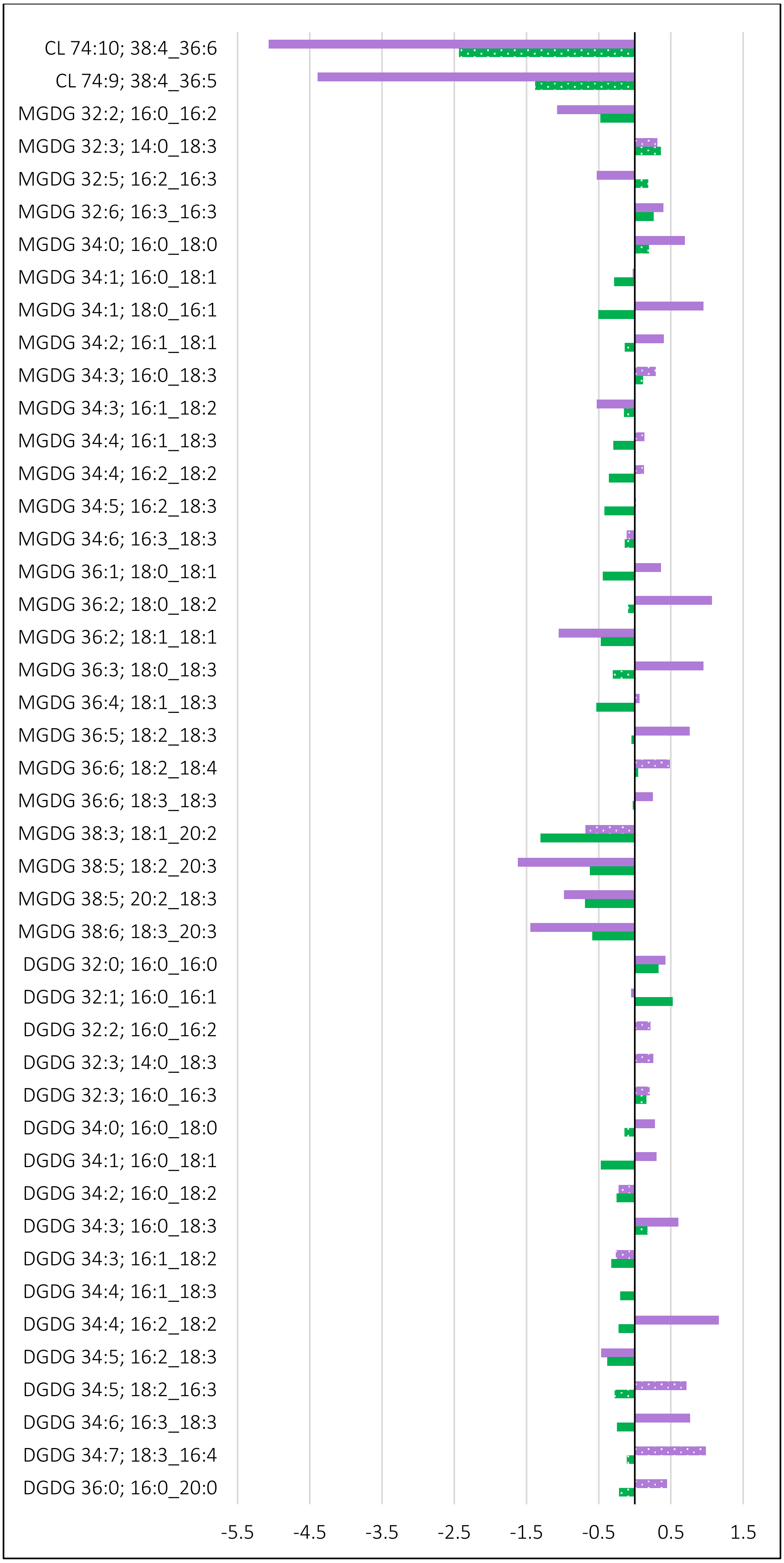

CL 74:10; 38:4_36:6
CL 74:9; 38:4_36:5
MGDG 32:2; 16:0_16:2
MGDG 32:3; 14:0_18:3
MGDG 32:5; 16:2_16:3
MGDG 32:6; 16:3_16:3
MGDG 34:0; 16:0_18:0
MGDG 34:1; 16:0_18:1
MGDG 34:1; 18:0_16:1
MGDG 34:2; 16:1_18:1
MGDG 34:3; 16:0_18:3
MGDG 34:3; 16:1_18:2
MGDG 34:4; 16:1_18:3
MGDG 34:4; 16:2_18:2
MGDG 34:5; 16:2_18:3
MGDG 34:6; 16:3_18:3
MGDG 36:1; 18:0_18:1
MGDG 36:2; 18:0_18:2
MGDG 36:2; 18:1_18:1
MGDG 36:3; 18:0_18:3
MGDG 36:4; 18:1_18:3
MGDG 36:5; 18:2_18:3
MGDG 36:6; 18:2_18:4
MGDG 36:6; 18:3_18:3
MGDG 38:3; 18:1_20:2
MGDG 38:5; 18:2_20:3
MGDG 38:5; 20:2_18:3
MGDG 38:6; 18:3_20:3
DGDG 32:0; 16:0_16:0
DGDG 32:1; 16:0_16:1
DGDG 32:2; 16:0_16:2
DGDG 32:3; 14:0_18:3
DGDG 32:3; 16:0_16:3
DGDG 34:0; 16:0_18:0
DGDG 34:1; 16:0_18:1
DGDG 34:2; 16:0_18:2
DGDG 34:3; 16:0_18:3
DGDG 34:3; 16:1_18:2
DGDG 34:4; 16:1_18:3
DGDG 34:4; 16:2_18:2
DGDG 34:5; 16:2_18:3
DGDG 34:5; 18:2_16:3
DGDG 34:6; 16:3_18:3
DGDG 34:7; 18:3_16:4
DGDG 36:0; 16:0_20:0
-5.5
-4.5
-3.5
-2.5
-1.5
-0.5
0.5
1.5

(e)

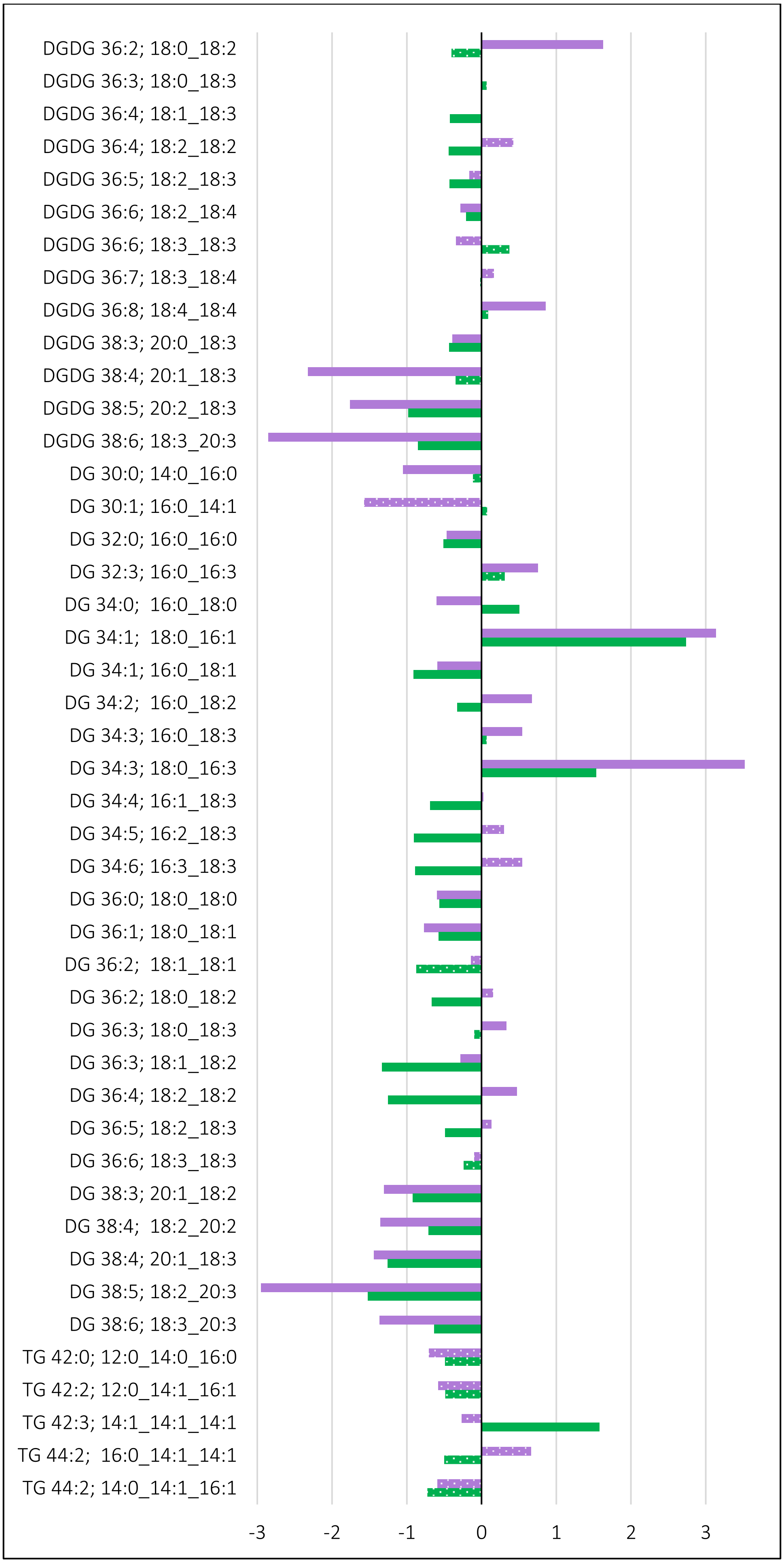
DGDG 36:2; 18:0_18:2
DGDG 36:3; 18:0_18:3
DGDG 36:4; 18:1_18:3
DGDG 36:4; 18:2_18:2
DGDG 36:5; 18:2_18:3
DGDG 36:6; 18:2_18:4
DGDG 36:6; 18:3_18:3
DGDG 36:7; 18:3_18:4
DGDG 36:8; 18:4_18:4
DGDG 38:3; 20:0_18:3
DGDG 38:4; 20:1_18:3
DGDG 38:5; 20:2_18:3
DGDG 38:6; 18:3_20:3
DG 30:0; 14:0_16:0
DG 30:1; 16:0_14:1
DG 32:0; 16:0_16:0
DG 32:3; 16:0_16:3
DG 34:0; 16:0_18:0
DG 34:1; 18:0_16:1
DG 34:1; 16:0_18:1
DG 34:2; 16:0_18:2
DG 34:3; 16:0_18:3
DG 34:3; 18:0_16:3
DG 34:4; 16:1_18:3
DG 34:5; 16:2_18:3
DG 34:6; 16:3_18:3
DG 36:0; 18:0_18:0
DG 36:1; 18:0_18:1
DG 36:2; 18:1_18:1
DG 36:2; 18:0_18:2
DG 36:3; 18:0_18:3
DG 36:3; 18:1_18:2
DG 36:4; 18:2_18:2
DG 36:5; 18:2_18:3
DG 36:6; 18:3_18:3
DG 38:3; 20:1_18:2
DG 38:4; 18:2_20:2
DG 38:4; 20:1_18:3
DG 38:5; 18:2_20:3
DG 38:6; 18:3_20:3
TG 42:0; 12:0_14:0_16:0
TG 42:2; 12:0_14:1_16:1
TG 42:3; 14:1_14:1_14:1
TG 44:2; 16:0_14:1_14:1
TG 44:2; 14:0_14:1_16:1
-3
-2
-1
0
1
2
3

(f)

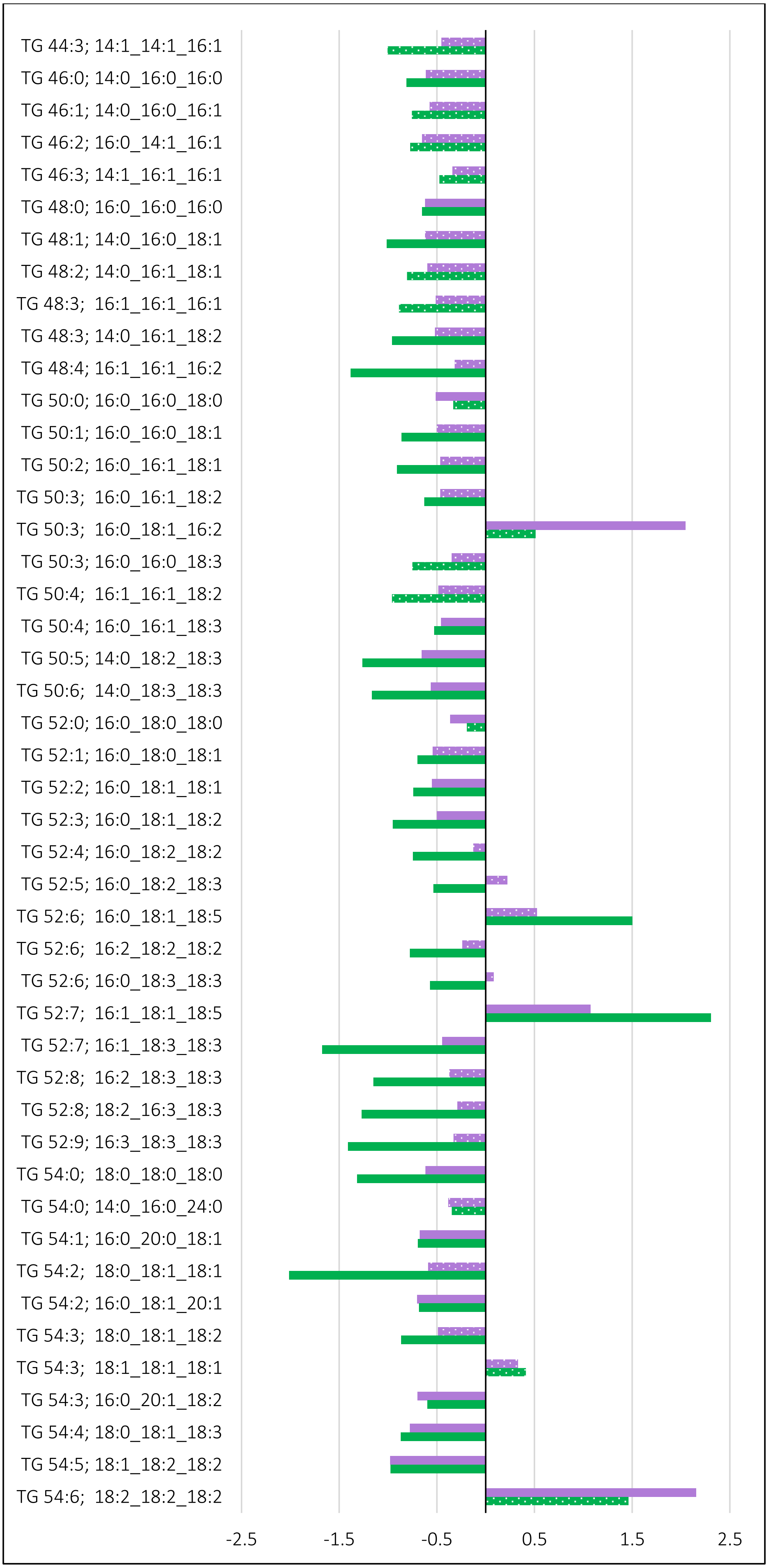

TG 44:3; 14:1_14:1_16:1
TG 46:0; 14:0_16:0_16:0
TG 46:1; 14:0_16:0_16:1
TG 46:2; 16:0_14:1_16:1
TG 46:3; 14:1_16:1_16:1
TG 48:0; 16:0_16:0_16:0
TG 48:1; 14:0_16:0_18:1
TG 48:2; 14:0_16:1_18:1
TG 48:3; 16:1_16:1_16:1
TG 48:3; 14:0_16:1_18:2
TG 48:4; 16:1_16:1_16:2
TG 50:0; 16:0_16:0_18:0
TG 50:1; 16:0_16:0_18:1
TG 50:2; 16:0_16:1_18:1
TG 50:3; 16:0_16:1_18:2
TG 50:3; 16:0_18:1_16:2
TG 50:3; 16:0_16:0_18:3
TG 50:4; 16:1_16:1_18:2
TG 50:4; 16:0_16:1_18:3
TG 50:5; 14:0_18:2_18:3
TG 50:6; 14:0_18:3_18:3
TG 52:0; 16:0_18:0_18:0
TG 52:1; 16:0_18:0_18:1
TG 52:2; 16:0_18:1_18:1
TG 52:3; 16:0_18:1_18:2
TG 52:4; 16:0_18:2_18:2
TG 52:5; 16:0_18:2_18:3
TG 52:6; 16:0_18:1_18:5
TG 52:6; 16:2_18:2_18:2
TG 52:6; 16:0_18:3_18:3
TG 52:7; 16:1_18:1_18:5
TG 52:7; 16:1_18:3_18:3
TG 52:8; 16:2_18:3_18:3
TG 52:8; 18:2_16:3_18:3
TG 52:9; 16:3_18:3_18:3
TG 54:0; 18:0_18:0_18:0
TG 54:0; 14:0_16:0_24:0
TG 54:1; 16:0_20:0_18:1
TG 54:2; 18:0_18:1_18:1
TG 54:2; 16:0_18:1_20:1
TG 54:3; 18:0_18:1_18:2
TG 54:3; 18:1_18:1_18:1
TG 54:3; 16:0_20:1_18:2
TG 54:4; 18:0_18:1_18:3
TG 54:5; 18:1_18:2_18:2
TG 54:6; 18:2_18:2_18:2
-2.5
-1.5
-0.5
0.5
1.5
2.5

(g)

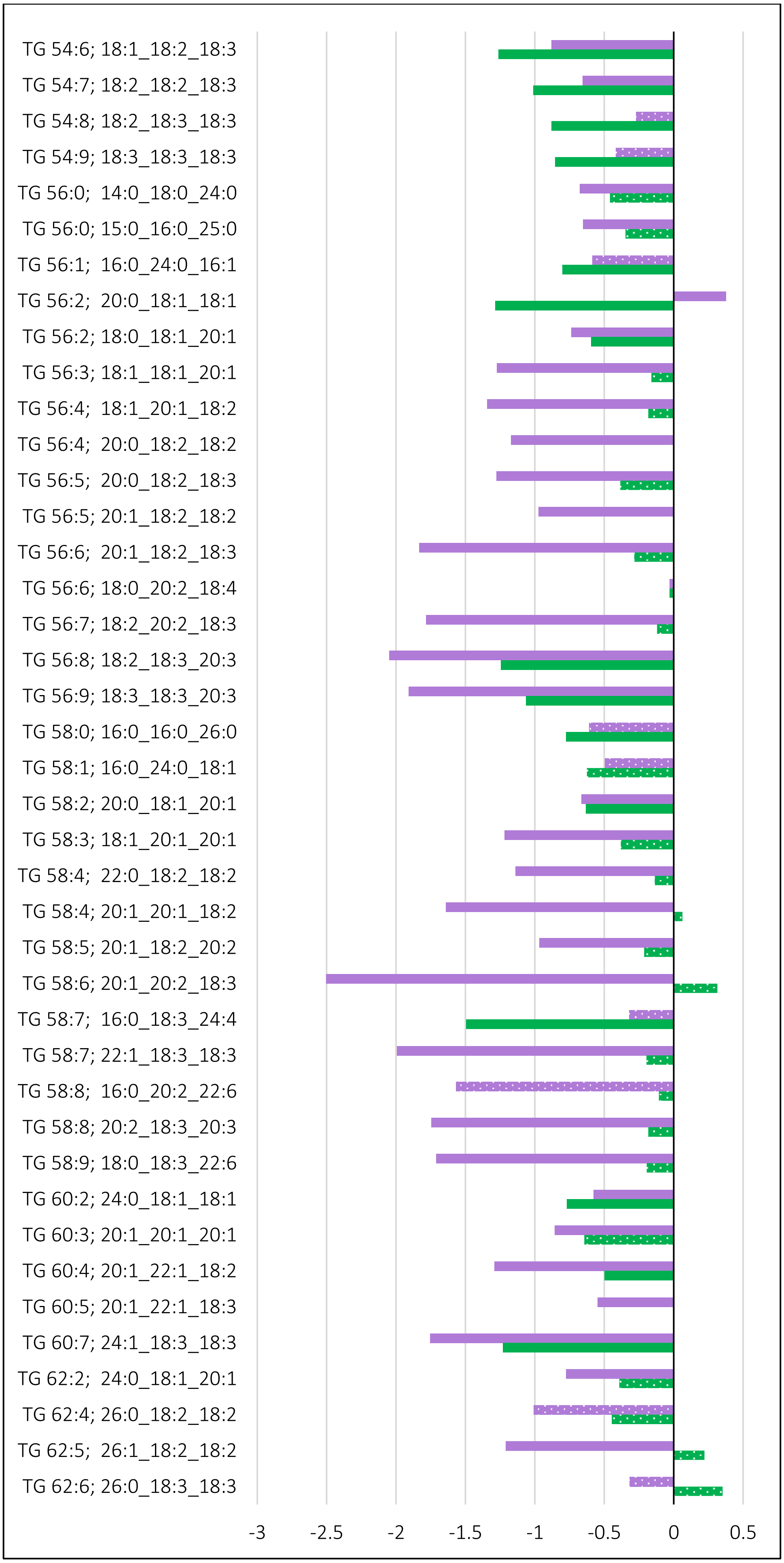
TG 54:6; 18:1_18:2_18:3
TG 54:7; 18:2_18:2_18:3
TG 54:8; 18:2_18:3_18:3
TG 54:9; 18:3_18:3_18:3
TG 56:0; 14:0_18:0_24:0
TG 56:0; 15:0_16:0_25:0
TG 56:1; 16:0_24:0_16:1
TG 56:2; 20:0_18:1_18:1
TG 56:2; 18:0_18:1_20:1
TG 56:3; 18:1_18:1_20:1
TG 56:4; 18:1_20:1_18:2
TG 56:4; 20:0_18:2_18:2
TG 56:5; 20:0_18:2_18:3
TG 56:5; 20:1_18:2_18:2
TG 56:6; 20:1_18:2_18:3
TG 56:6; 18:0_20:2_18:4
TG 56:7; 18:2_20:2_18:3
TG 56:8; 18:2_18:3_20:3
TG 56:9; 18:3_18:3_20:3
TG 58:0; 16:0_16:0_26:0
TG 58:1; 16:0_24:0_18:1
TG 58:2; 20:0_18:1_20:1
TG 58:3; 18:1_20:1_20:1
TG 58:4; 22:0_18:2_18:2
TG 58:4; 20:1_20:1_18:2
TG 58:5; 20:1_18:2_20:2
TG 58:6; 20:1_20:2_18:3
TG 58:7; 16:0_18:3_24:4
TG 58:7; 22:1_18:3_18:3
TG 58:8; 16:0_20:2_22:6
TG 58:8; 20:2_18:3_20:3
TG 58:9; 18:0_18:3_22:6
TG 60:2; 24:0_18:1_18:1
TG 60:3; 20:1_20:1_20:1
TG 60:4; 20:1_22:1_18:2
TG 60:5; 20:1_22:1_18:3
TG 60:7; 24:1_18:3_18:3
TG 62:2; 24:0_18:1_20:1
TG 62:4; 26:0_18:2_18:2
TG 62:5; 26:1_18:2_18:2
TG 62:6; 26:0_18:3_18:3
-3
-2.5
-2
-1.5
-1
-0.5
0
0.5

Figure S4 (a-f) Log2 fold change comparison of the levels of lipid species in the first internode compared to the second internode of 28 d old plants (Log_2 first internode/ second internode)

(a)

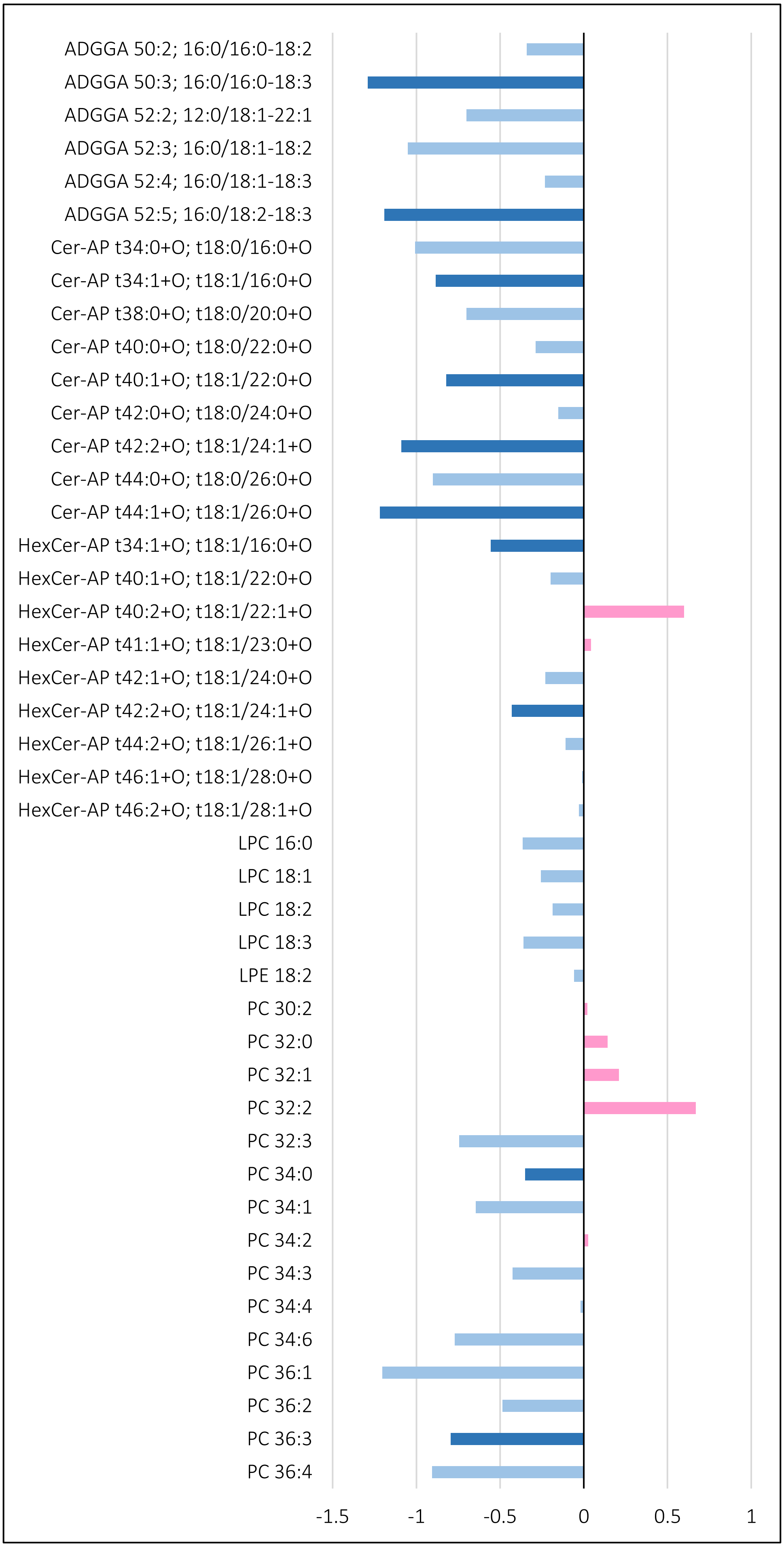

(b)

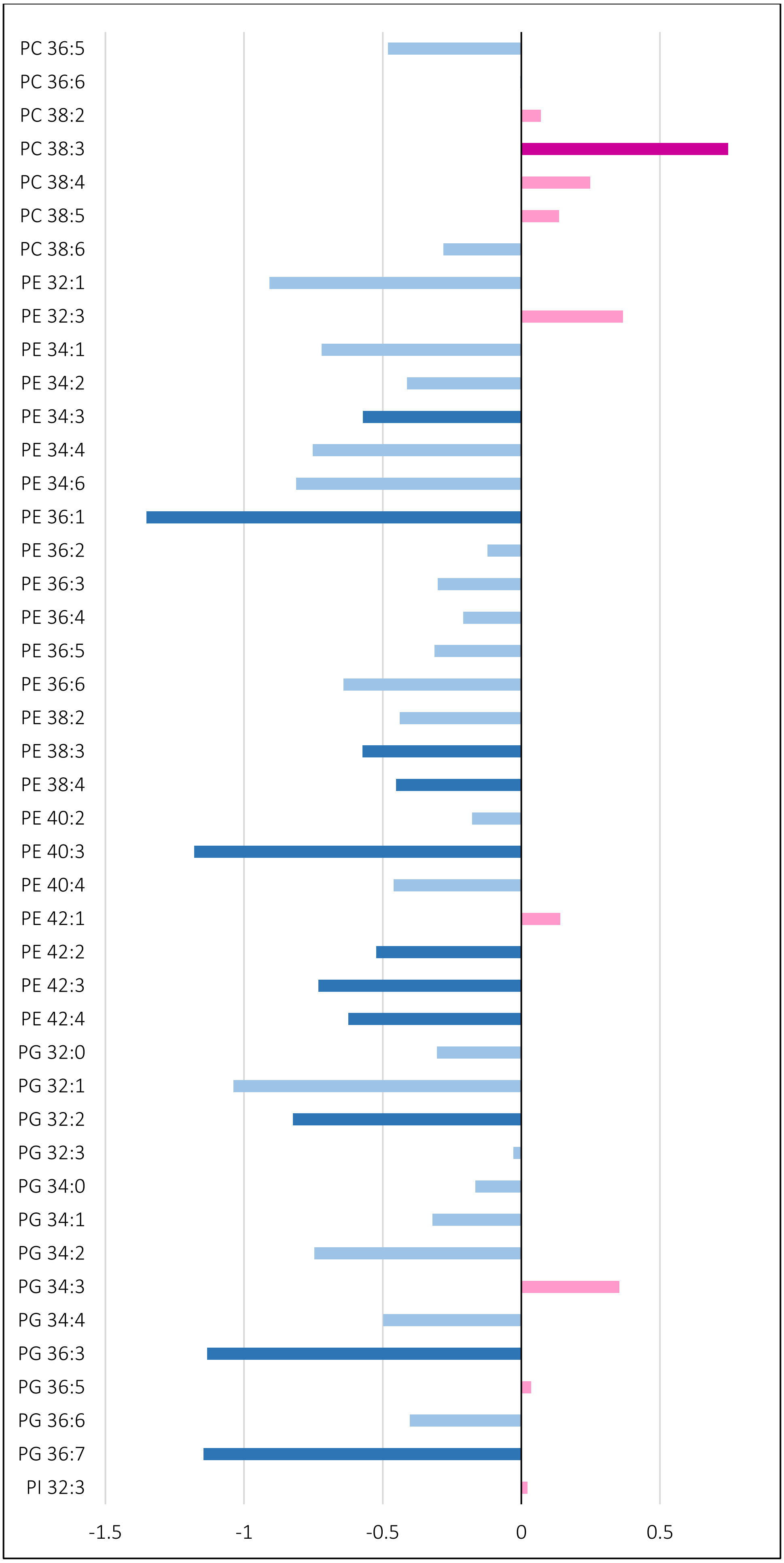

PC 36:5
PC 36:6
PC 38:2
PC 38:3
PC 38:4
PC 38:5
PC 38:6
PE 32:1
PE 32:3
PE 34:1
PE 34:2
PE 34:3
PE 34:4
PE 34:6
PE 36:1
PE 36:2
PE 36:3
PE 36:4
PE 36:5
PE 36:6
PE 38:2
PE 38:3
PE 38:4
PE 40:2
PE 40:3
PE 40:4
PE 42:1
PE 42:2
PE 42:3
PE 42:4
PG 32:0
PG 32:1
PG 32:2
PG 32:3
PG 34:0
PG 34:1
PG 34:2
PG 34:3
PG 34:4
PG 36:3
PG 36:5
PG 36:6
PG 36:7
PI 32:3
-1.5
-1
-0.5
0
0.5

(c)

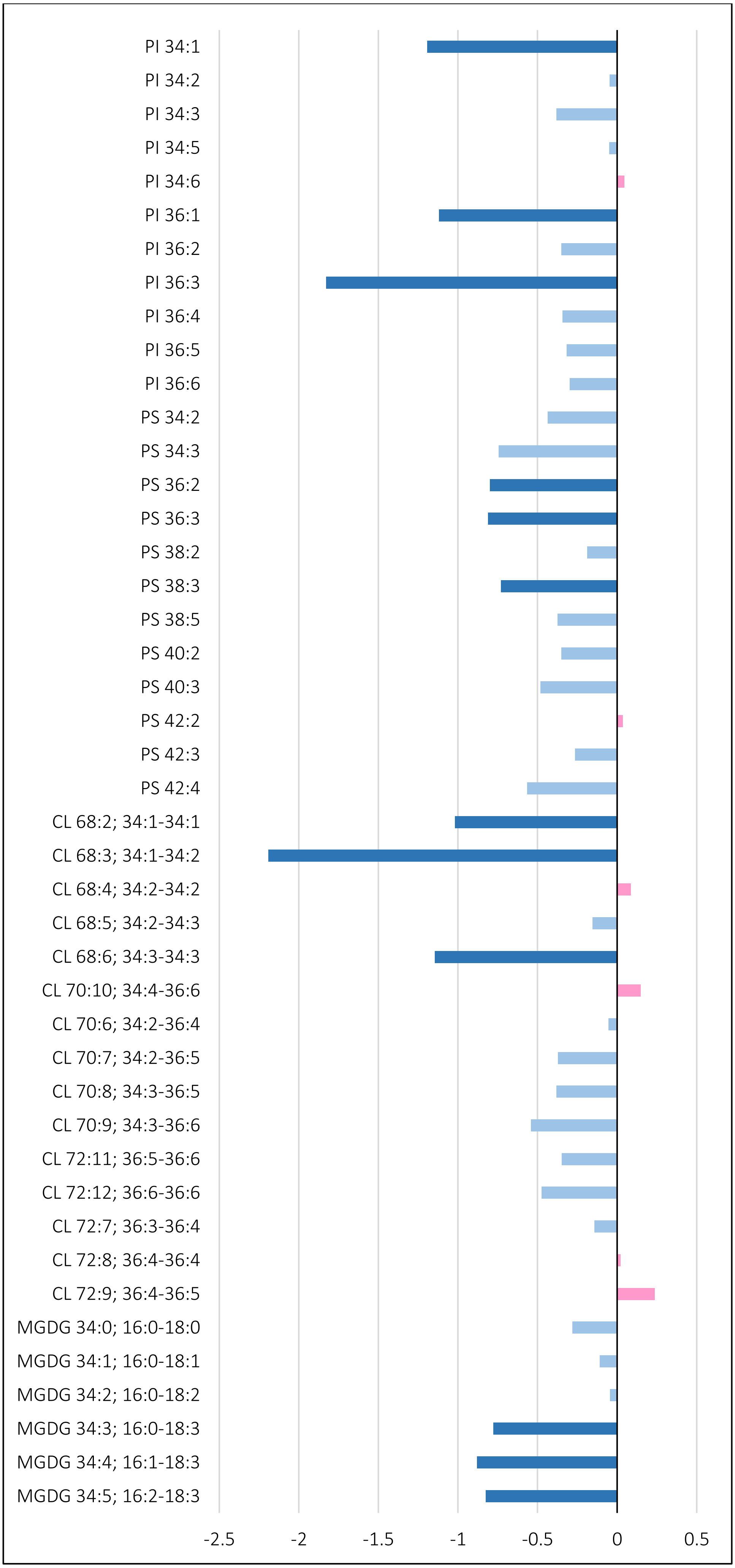

PI 34:1
PI 34:2
PI 34:3
PI 34:5
PI 34:6
PI 36:1
PI 36:2
PI 36:3
PI 36:4
PI 36:5
PI 36:6
PS 34:2
PS 34:3
PS 36:2
PS 36:3
PS 38:2
PS 38:3
PS 38:5
PS 40:2
PS 40:3
PS 42:2
PS 42:3
PS 42:4
CL 68:2; 34:1-34:1
CL 68:3; 34:1-34:2
CL 68:4; 34:2-34:2
CL 68:5; 34:2-34:3
CL 68:6; 34:3-34:3
CL 70:10; 34:4-36:6
CL 70:6; 34:2-36:4
CL 70:7; 34:2-36:5
CL 70:8; 34:3-36:5
CL 70:9; 34:3-36:6
CL 72:11; 36:5-36:6
CL 72:12; 36:6-36:6
CL 72:7; 36:3-36:4
CL 72:8; 36:4-36:4
CL 72:9; 36:4-36:5
MGDG 34:0; 16:0-18:0
MGDG 34:1; 16:0-18:1
MGDG 34:2; 16:0-18:2
MGDG 34:3; 16:0-18:3
MGDG 34:4; 16:1-18:3
MGDG 34:5; 16:2-18:3
-2.5
-2
-1.5
-1
-0.5
0
0.5

(d)

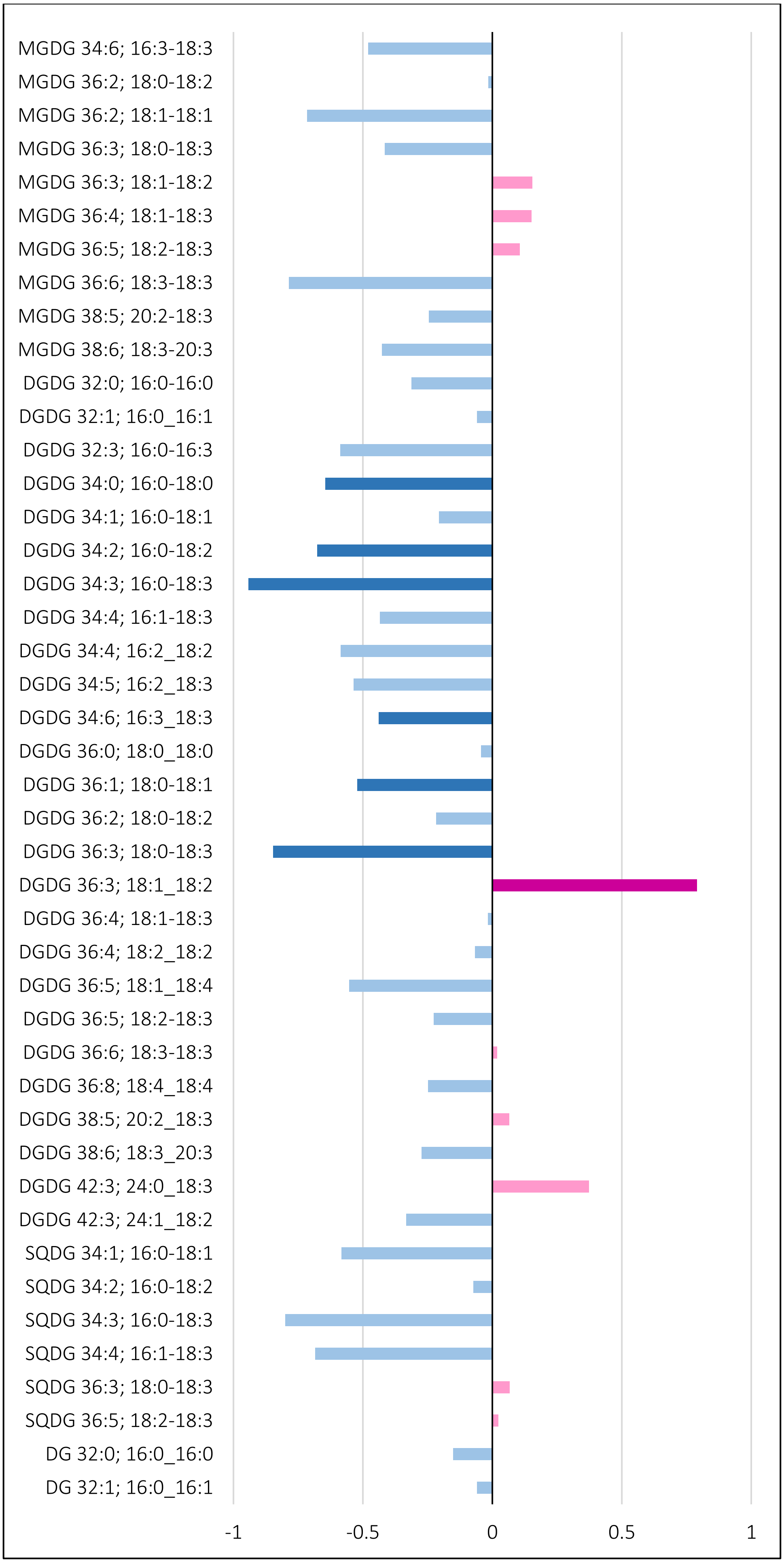

MGDG 34:6; 16:3-18:3
MGDG 36:2; 18:0-18:2
MGDG 36:2; 18:1-18:1
MGDG 36:3; 18:0-18:3
MGDG 36:3; 18:1-18:2
MGDG 36:4; 18:1-18:3
MGDG 36:5; 18:2-18:3
MGDG 36:6; 18:3-18:3
MGDG 38:5; 20:2-18:3
MGDG 38:6; 18:3-20:3
DGDG 32:0; 16:0-16:0
DGDG 32:1; 16:0_16:1
DGDG 32:3; 16:0-16:3
DGDG 34:0; 16:0-18:0
DGDG 34:1; 16:0-18:1
DGDG 34:2; 16:0-18:2
DGDG 34:3; 16:0-18:3
DGDG 34:4; 16:1-18:3
DGDG 34:4; 16:2_18:2
DGDG 34:5; 16:2_18:3
DGDG 34:6; 16:3_18:3
DGDG 36:0; 18:0_18:0
DGDG 36:1; 18:0-18:1
DGDG 36:2; 18:0-18:2
DGDG 36:3; 18:0-18:3
DGDG 36:3; 18:1_18:2
DGDG 36:4; 18:1-18:3
DGDG 36:4; 18:2_18:2
DGDG 36:5; 18:1_18:4
DGDG 36:5; 18:2-18:3
DGDG 36:6; 18:3-18:3
DGDG 36:8; 18:4_18:4
DGDG 38:5; 20:2_18:3
DGDG 38:6; 18:3_20:3
DGDG 42:3; 24:0_18:3
DGDG 42:3; 24:1_18:2
SQDG 34:1; 16:0-18:1
SQDG 34:2; 16:0-18:2
SQDG 34:3; 16:0-18:3
SQDG 34:4; 16:1-18:3
SQDG 36:3; 18:0-18:3
SQDG 36:5; 18:2-18:3
DG 32:0; 16:0_16:0
DG 32:1; 16:0_16:1
-1
-0.5
0
0.5
1

(e)

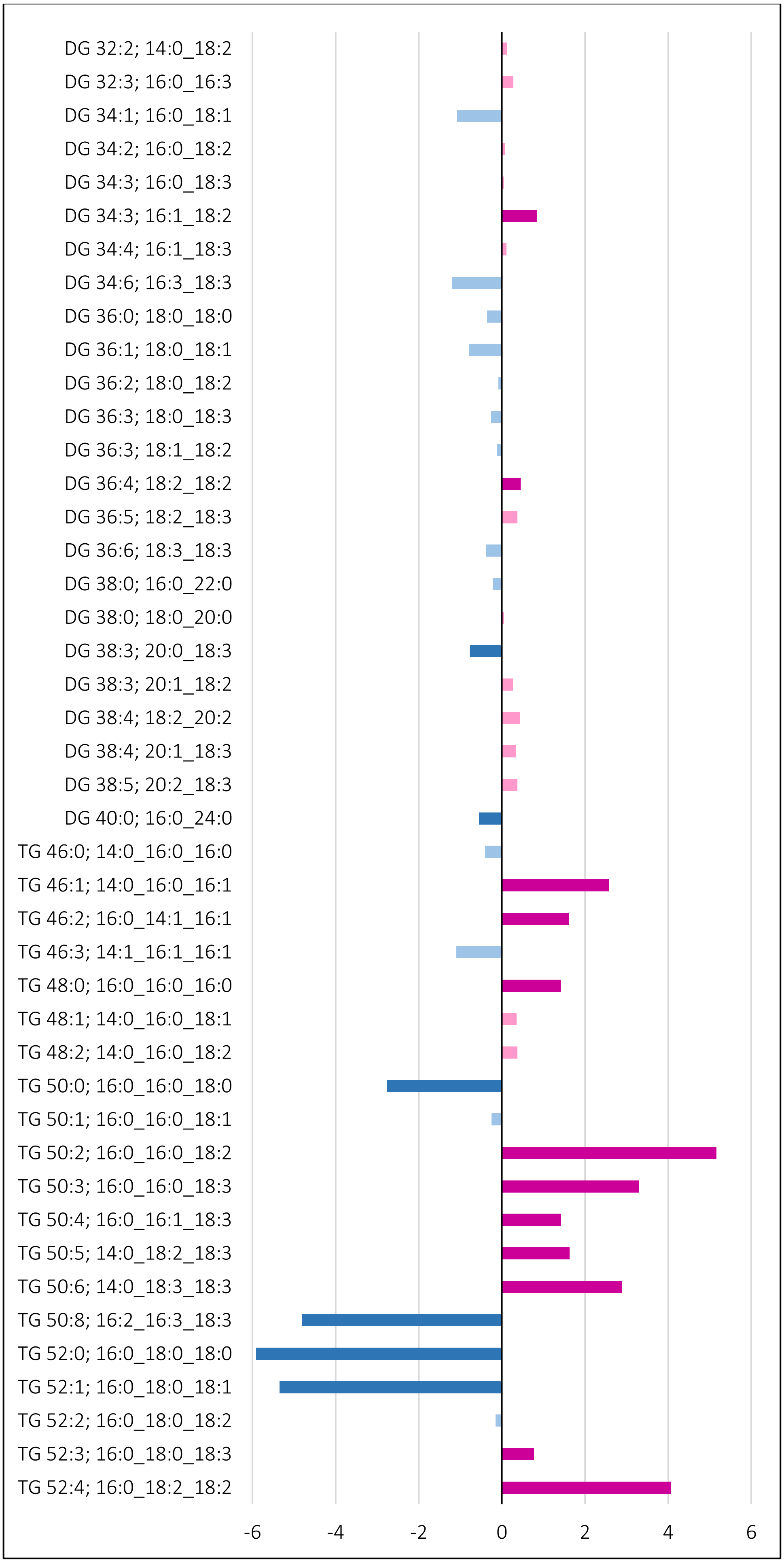
DG 32:2; 14:0_18:2
DG 32:3; 16:0_16:3
DG 34:1; 16:0_18:1
DG 34:2; 16:0_18:2
DG 34:3; 16:0_18:3
DG 34:3; 16:1_18:2
DG 34:4; 16:1_18:3
DG 34:6; 16:3_18:3
DG 36:0; 18:0_18:0
DG 36:1; 18:0_18:1
DG 36:2; 18:0_18:2
DG 36:3; 18:0_18:3
DG 36:3; 18:1_18:2
DG 36:4; 18:2_18:2
DG 36:5; 18:2_18:3
DG 36:6; 18:3_18:3
DG 38:0; 16:0_22:0
DG 38:0; 18:0_20:0
DG 38:3; 20:0_18:3
DG 38:3; 20:1_18:2
DG 38:4; 18:2_20:2
DG 38:4; 20:1_18:3
DG 38:5; 20:2_18:3
DG 40:0; 16:0_24:0
TG 46:0; 14:0_16:0_16:0
TG 46:1; 14:0_16:0_16:1
TG 46:2; 16:0_14:1_16:1
TG 46:3; 14:1_16:1_16:1
TG 48:0; 16:0_16:0_16:0
TG 48:1; 14:0_16:0_18:1
TG 48:2; 14:0_16:0_18:2
TG 50:0; 16:0_16:0_18:0
TG 50:1; 16:0_16:0_18:1
TG 50:2; 16:0_16:0_18:2
TG 50:3; 16:0_16:0_18:3
TG 50:4; 16:0_16:1_18:3
TG 50:5; 14:0_18:2_18:3
TG 50:6; 14:0_18:3_18:3
TG 50:8; 16:2_16:3_18:3
TG 52:0; 16:0_18:0_18:0
TG 52:1; 16:0_18:0_18:1
TG 52:2; 16:0_18:0_18:2
TG 52:3; 16:0_18:0_18:3
TG 52:4; 16:0_18:2_18:2
-6
-4
-2
0
2
4
6

(f)

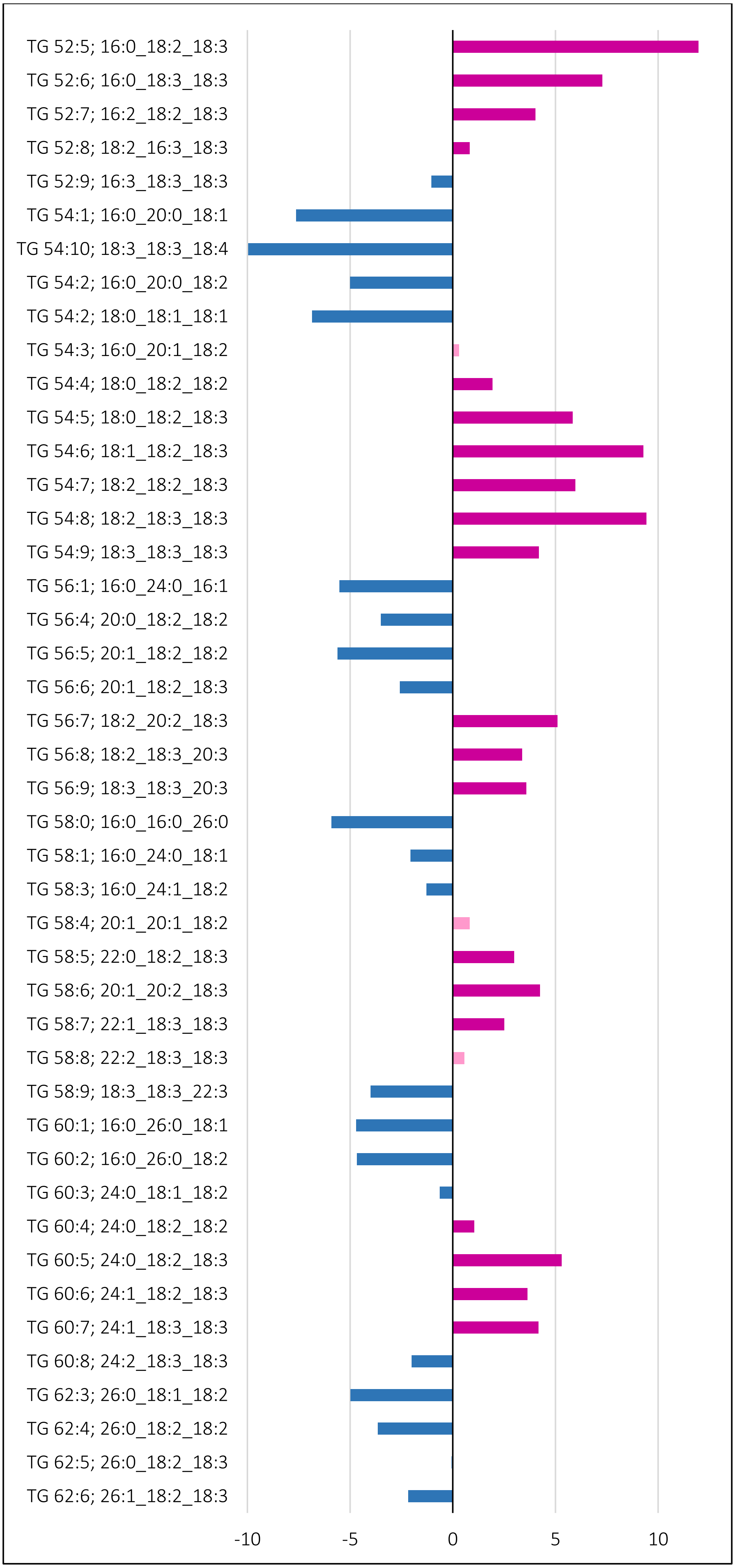
TG 52:5; 16:0_18:2_18:3
TG 52:6; 16:0_18:3_18:3
TG 52:7; 16:2_18:2_18:3
TG 52:8; 18:2_16:3_18:3
TG 52:9; 16:3_18:3_18:3
TG 54:1; 16:0_20:0_18:1
TG 54:10; 18:3_18:3_18:4
TG 54:2; 16:0_20:0_18:2
TG 54:2; 18:0_18:1_18:1
TG 54:3; 16:0_20:1_18:2
TG 54:4; 18:0_18:2_18:2
TG 54:5; 18:0_18:2_18:3
TG 54:6; 18:1_18:2_18:3
TG 54:7; 18:2_18:2_18:3
TG 54:8; 18:2_18:3_18:3
TG 54:9; 18:3_18:3_18:3
TG 56:1; 16:0_24:0_16:1
TG 56:4; 20:0_18:2_18:2
TG 56:5; 20:1_18:2_18:2
TG 56:6; 20:1_18:2_18:3
TG 56:7; 18:2_20:2_18:3
TG 56:8; 18:2_18:3_20:3
TG 56:9; 18:3_18:3_20:3
TG 58:0; 16:0_16:0_26:0
TG 58:1; 16:0_24:0_18:1
TG 58:3; 16:0_24:1_18:2
TG 58:4; 20:1_20:1_18:2
TG 58:5; 22:0_18:2_18:3
TG 58:6; 20:1_20:2_18:3
TG 58:7; 22:1_18:3_18:3
TG 58:8; 22:2_18:3_18:3
TG 58:9; 18:3_18:3_22:3
TG 60:1; 16:0_26:0_18:1
TG 60:2; 16:0_26:0_18:2
TG 60:3; 24:0_18:1_18:2
TG 60:4; 24:0_18:2_18:2
TG 60:5; 24:0_18:2_18:3
TG 60:6; 24:1_18:2_18:3
TG 60:7; 24:1_18:3_18:3
TG 60:8; 24:2_18:3_18:3
TG 62:3; 26:0_18:1_18:2
TG 62:4; 26:0_18:2_18:2
TG 62:5; 26:0_18:2_18:3
TG 62:6; 26:1_18:2_18:3
-10
-5
0
5
10

Figure S5 (a-h) Log2 fold change comparison of the levels of lipid species in 1.2 cm long siliques compared to 1 cm long siliques (Log_2 1.2 cm/1 cm) (blue bars) and in 1.5 cm long siliques compared to 1.2 cm long siliques (Log_2 1.5 cm/ 1.2 cm) (green bars)

(a)

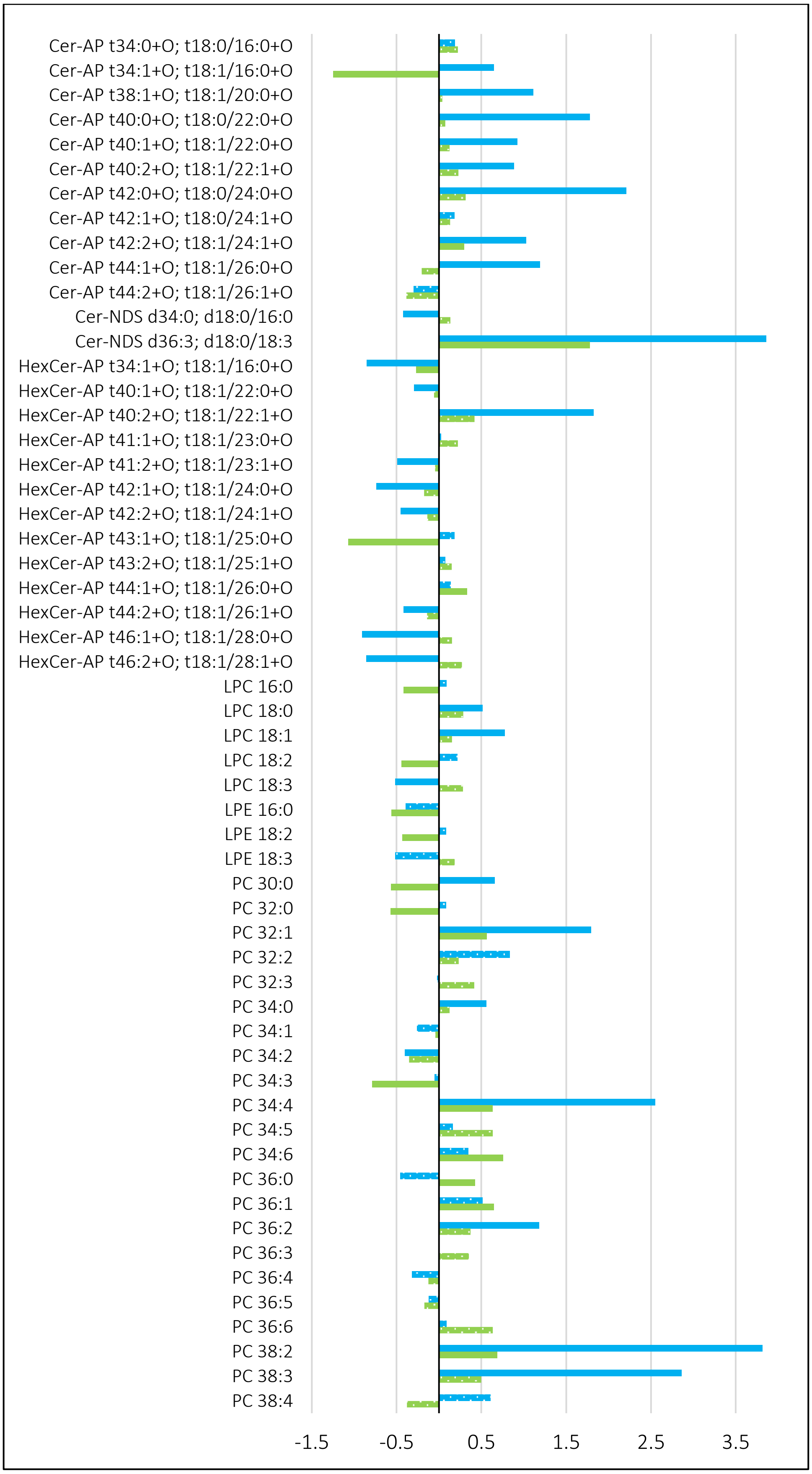

(b)

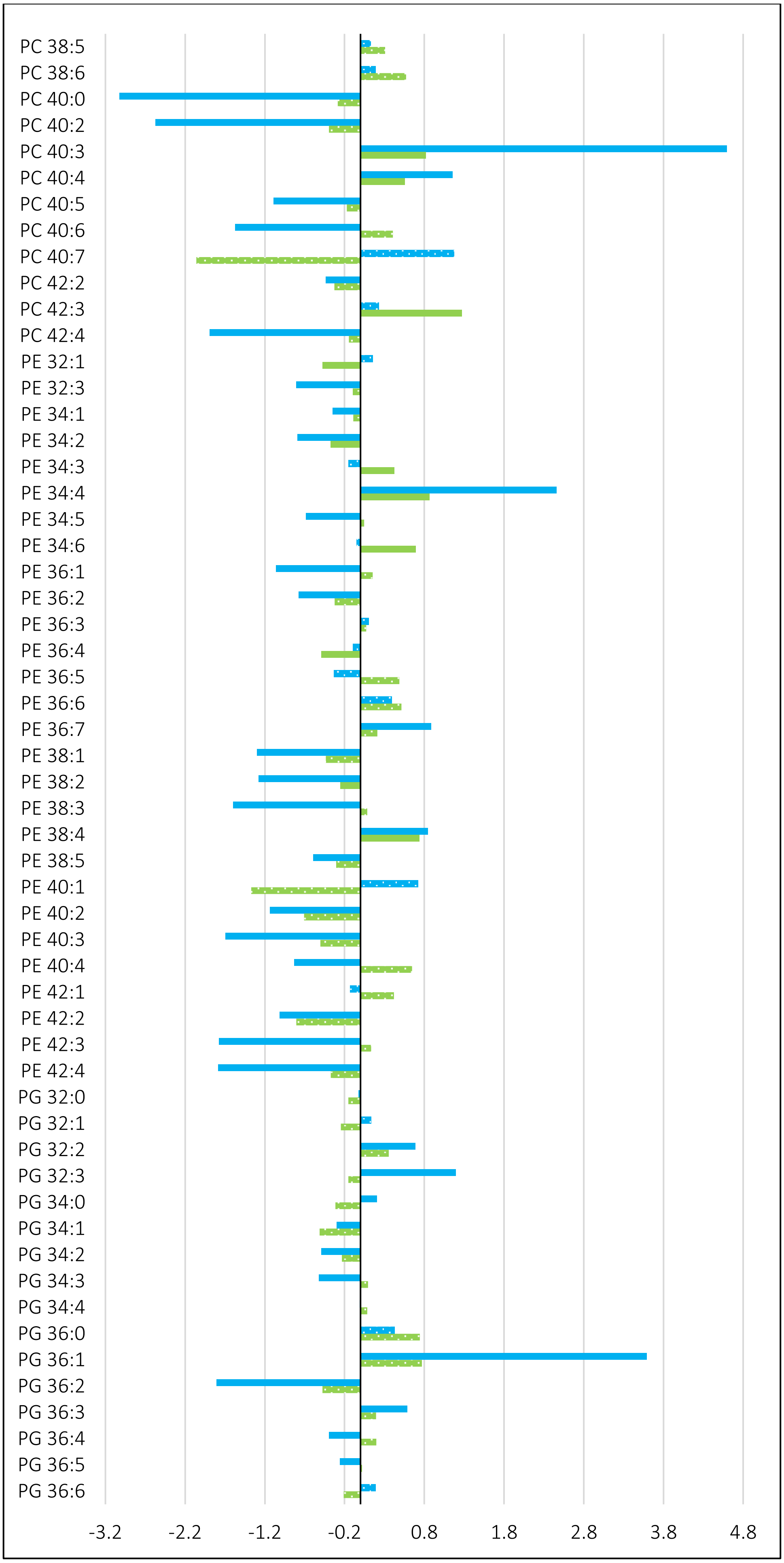
PC 38:5
PC 38:6
PC 40:0
PC 40:2
PC 40:3
PC 40:4
PC 40:5
PC 40:6
PC 40:7
PC 42:2
PC 42:3
PC 42:4
PE 32:1
PE 32:3
PE 34:1
PE 34:2
PE 34:3
PE 34:4
PE 34:5
PE 34:6
PE 36:1
PE 36:2
PE 36:3
PE 36:4
PE 36:5
PE 36:6
PE 36:7
PE 38:1
PE 38:2
PE 38:3
PE 38:4
PE 38:5
PE 40:1
PE 40:2
PE 40:3
PE 40:4
PE 42:1
PE 42:2
PE 42:3
PE 42:4
PG 32:0
PG 32:1
PG 32:2
PG 32:3
PG 34:0
PG 34:1
PG 34:2
PG 34:3
PG 34:4
PG 36:0
PG 36:1
PG 36:2
PG 36:3
PG 36:4
PG 36:5
PG 36:6
-3.2
-2.2
-1.2
-0.2
0.8
1.8
2.8
3.8
4.8

(c)

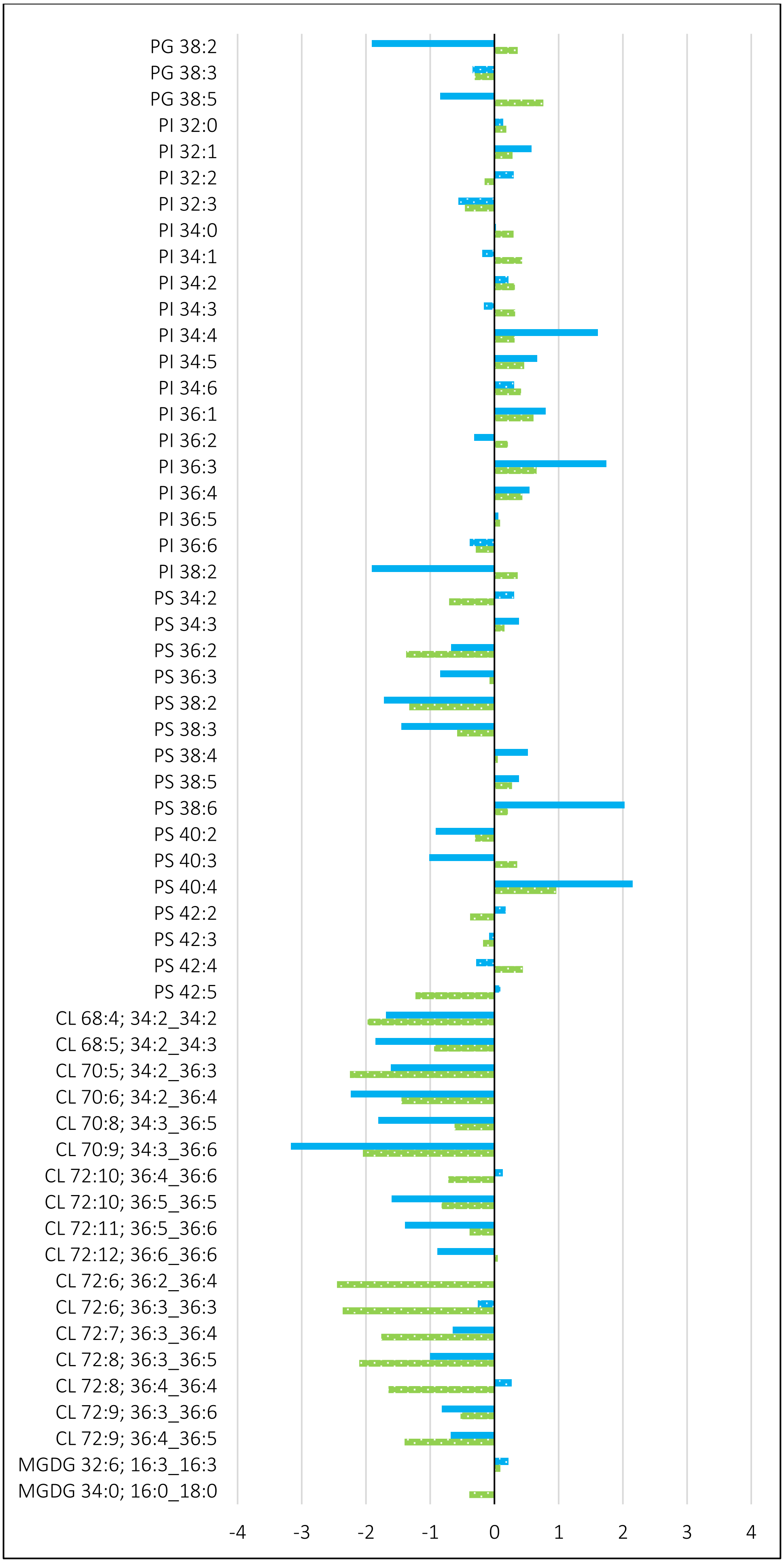
PG 38:2
PG 38:3
PG 38:5
PI 32:0
PI 32:1
PI 32:2
PI 32:3
PI 34:0
PI 34:1
PI 34:2
PI 34:3
PI 34:4
PI 34:5
PI 34:6
PI 36:1
PI 36:2
PI 36:3
PI 36:4
PI 36:5
PI 36:6
PI 38:2
PS 34:2
PS 34:3
PS 36:2
PS 36:3
PS 38:2
PS 38:3
PS 38:4
PS 38:5
PS 38:6
PS 40:2
PS 40:3
PS 40:4
PS 42:2
PS 42:3
PS 42:4
PS 42:5
CL 68:4; 34:2_34:2
CL 68:5; 34:2_34:3
CL 70:5; 34:2_36:3
CL 70:6; 34:2_36:4
CL 70:8; 34:3_36:5
CL 70:9; 34:3_36:6
CL 72:10; 36:4_36:6
CL 72:10; 36:5_36:5
CL 72:11; 36:5_36:6
CL 72:12; 36:6_36:6
CL 72:6; 36:2_36:4
CL 72:6; 36:3_36:3
CL 72:7; 36:3_36:4
CL 72:8; 36:3_36:5
CL 72:8; 36:4_36:4
CL 72:9; 36:3_36:6
CL 72:9; 36:4_36:5
MGDG 32:6; 16:3_16:3
MGDG 34:0; 16:0_18:0
-4
-3
-2
-1
0
1
2
3
4

(d)

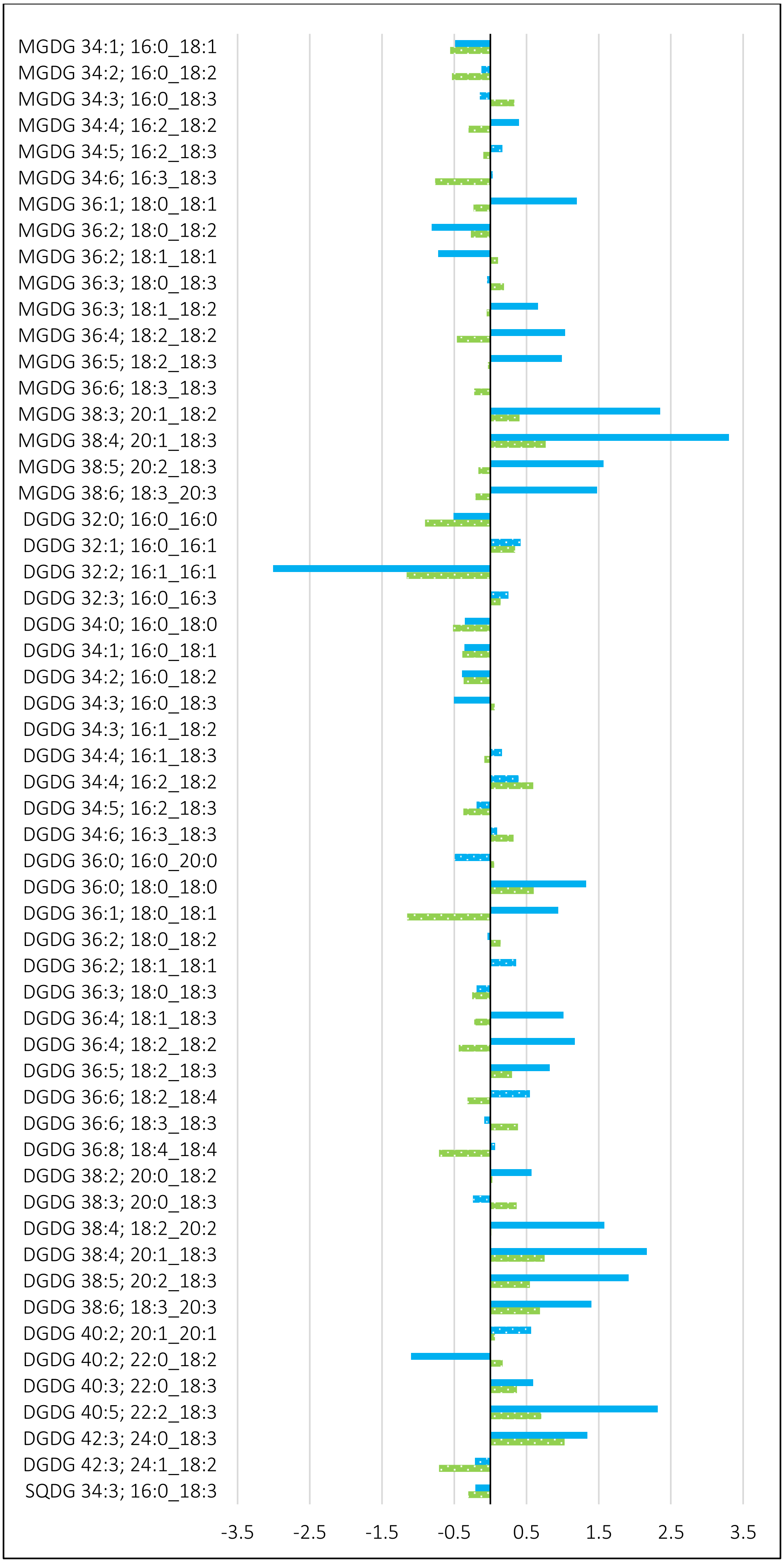
MGDG 34:1; 16:0_18:1
MGDG 34:2; 16:0_18:2
MGDG 34:3; 16:0_18:3
MGDG 34:4; 16:2_18:2
MGDG 34:5; 16:2_18:3
MGDG 34:6; 16:3_18:3
MGDG 36:1; 18:0_18:1
MGDG 36:2; 18:0_18:2
MGDG 36:2; 18:1_18:1
MGDG 36:3; 18:0_18:3
MGDG 36:3; 18:1_18:2
MGDG 36:4; 18:2_18:2
MGDG 36:5; 18:2_18:3
MGDG 36:6; 18:3_18:3
MGDG 38:3; 20:1_18:2
MGDG 38:4; 20:1_18:3
MGDG 38:5; 20:2_18:3
MGDG 38:6; 18:3_20:3
DGDG 32:0; 16:0_16:0
DGDG 32:1; 16:0_16:1
DGDG 32:2; 16:1_16:1
DGDG 32:3; 16:0_16:3
DGDG 34:0; 16:0_18:0
DGDG 34:1; 16:0_18:1
DGDG 34:2; 16:0_18:2
DGDG 34:3; 16:0_18:3
DGDG 34:3; 16:1_18:2
DGDG 34:4; 16:1_18:3
DGDG 34:4; 16:2_18:2
DGDG 34:5; 16:2_18:3
DGDG 34:6; 16:3_18:3
DGDG 36:0; 16:0_20:0
DGDG 36:0; 18:0_18:0
DGDG 36:1; 18:0_18:1
DGDG 36:2; 18:0_18:2
DGDG 36:2; 18:1_18:1
DGDG 36:3; 18:0_18:3
DGDG 36:4; 18:1_18:3
DGDG 36:4; 18:2_18:2
DGDG 36:5; 18:2_18:3
DGDG 36:6; 18:2_18:4
DGDG 36:6; 18:3_18:3
DGDG 36:8; 18:4_18:4
DGDG 38:2; 20:0_18:2
DGDG 38:3; 20:0_18:3
DGDG 38:4; 18:2_20:2
DGDG 38:4; 20:1_18:3
DGDG 38:5; 20:2_18:3
DGDG 38:6; 18:3_20:3
DGDG 40:2; 20:1_20:1
DGDG 40:2; 22:0_18:2
DGDG 40:3; 22:0_18:3
DGDG 40:5; 22:2_18:3
DGDG 42:3; 24:0_18:3
DGDG 42:3; 24:1_18:2
SQDG 34:3; 16:0_18:3
-3.5
-2.5
-1.5
-0.5
0.5
1.5
2.5
3.5

(e)

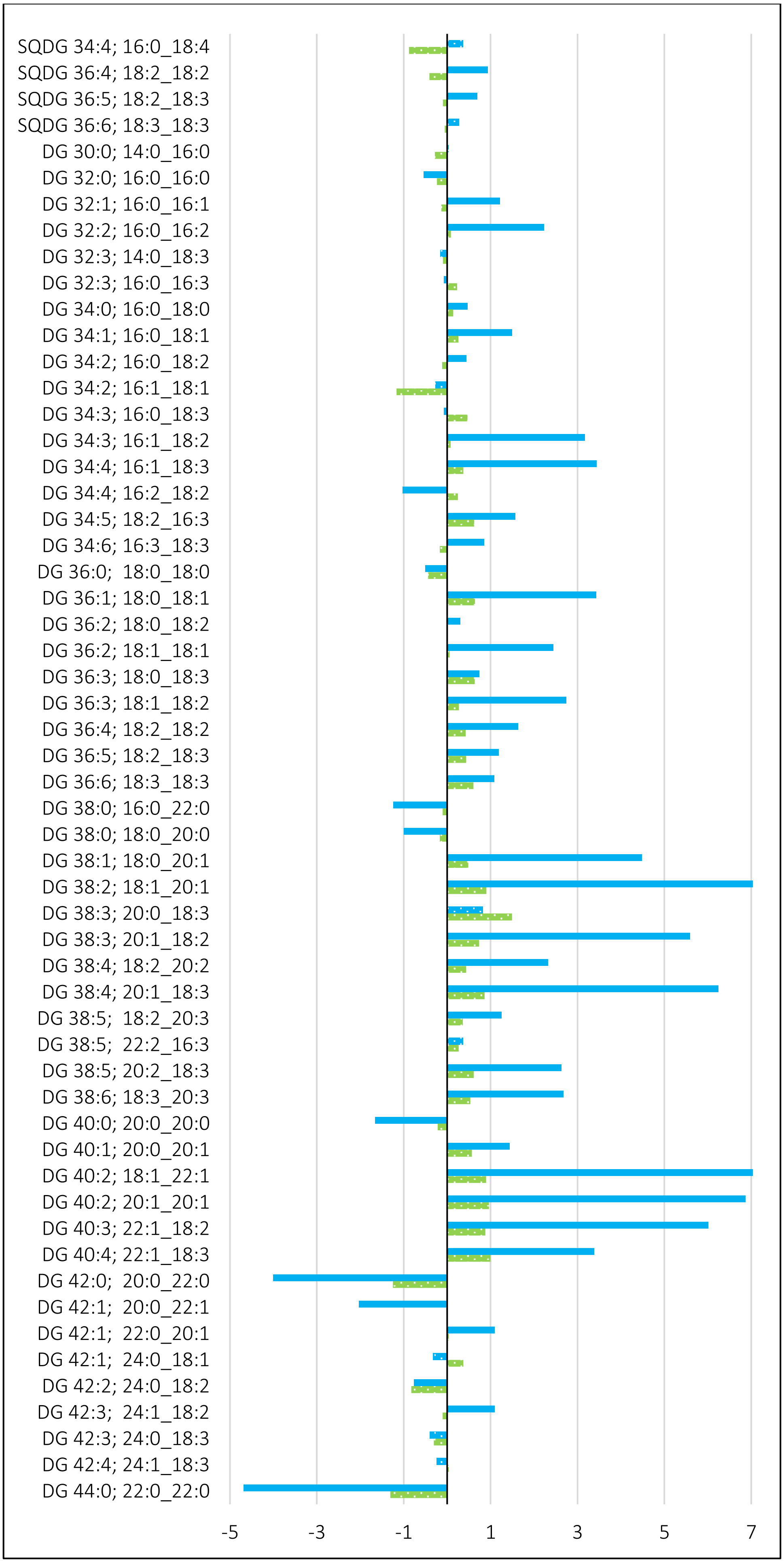

SQDG 34:4; 16:0_18:4
SQDG 36:4; 18:2_18:2
SQDG 36:5; 18:2_18:3
SQDG 36:6; 18:3_18:3
DG 30:0; 14:0_16:0
DG 32:0; 16:0_16:0
DG 32:1; 16:0_16:1
DG 32:2; 16:0_16:2
DG 32:3; 14:0_18:3
DG 32:3; 16:0_16:3
DG 34:0; 16:0_18:0
DG 34:1; 16:0_18:1
DG 34:2; 16:0_18:2
DG 34:2; 16:1_18:1
DG 34:3; 16:0_18:3
DG 34:3; 16:1_18:2
DG 34:4; 16:1_18:3
DG 34:4; 16:2_18:2
DG 34:5; 18:2_16:3
DG 34:6; 16:3_18:3
DG 36:0; 18:0_18:0
DG 36:1; 18:0_18:1
DG 36:2; 18:0_18:2
DG 36:2; 18:1_18:1
DG 36:3; 18:0_18:3
DG 36:3; 18:1_18:2
DG 36:4; 18:2_18:2
DG 36:5; 18:2_18:3
DG 36:6; 18:3_18:3
DG 38:0; 16:0_22:0
DG 38:0; 18:0_20:0
DG 38:1; 18:0_20:1
DG 38:2; 18:1_20:1
DG 38:3; 20:0_18:3
DG 38:3; 20:1_18:2
DG 38:4; 18:2_20:2
DG 38:4; 20:1_18:3
DG 38:5; 18:2_20:3
DG 38:5; 22:2_16:3
DG 38:5; 20:2_18:3
DG 38:6; 18:3_20:3
DG 40:0; 20:0_20:0
DG 40:1; 20:0_20:1
DG 40:2; 18:1_22:1
DG 40:2; 20:1_20:1
DG 40:3; 22:1_18:2
DG 40:4; 22:1_18:3
DG 42:0; 20:0_22:0
DG 42:1; 20:0_22:1
DG 42:1; 22:0_20:1
DG 42:1; 24:0_18:1
DG 42:2; 24:0_18:2
DG 42:3; 24:1_18:2
DG 42:3; 24:0_18:3
DG 42:4; 24:1_18:3
DG 44:0; 22:0_22:0
-5
-3
-1
1
3
5
7

(f)

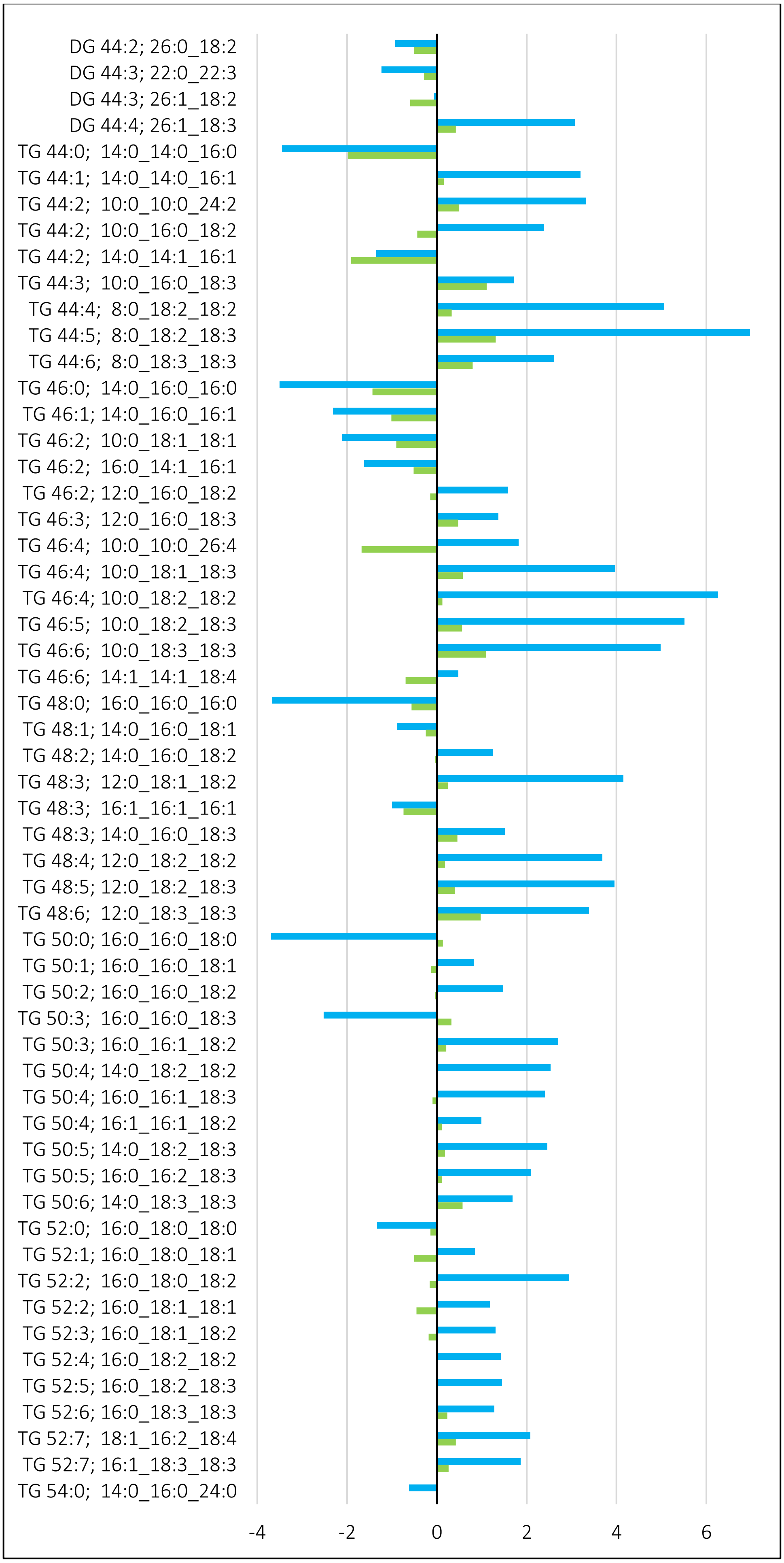
DG 44:2; 26:0_18:2
DG 44:3; 22:0_22:3
DG 44:3; 26:1_18:2
DG 44:4; 26:1_18:3
TG 44:0; 14:0_14:0_16:0
TG 44:1; 14:0_14:0_16:1
TG 44:2; 10:0_10:0_24:2
TG 44:2; 10:0_16:0_18:2
TG 44:2; 14:0_14:1_16:1
TG 44:3; 10:0_16:0_18:3
TG 44:4; 8:0_18:2_18:2
TG 44:5; 8:0_18:2_18:3
TG 44:6; 8:0_18:3_18:3
TG 46:0; 14:0_16:0_16:0
TG 46:1; 14:0_16:0_16:1
TG 46:2; 10:0_18:1_18:1
TG 46:2; 16:0_14:1_16:1
TG 46:2; 12:0_16:0_18:2
TG 46:3; 12:0_16:0_18:3
TG 46:4; 10:0_10:0_26:4
TG 46:4; 10:0_18:1_18:3
TG 46:4; 10:0_18:2_18:2
TG 46:5; 10:0_18:2_18:3
TG 46:6; 10:0_18:3_18:3
TG 46:6; 14:1_14:1_18:4
TG 48:0; 16:0_16:0_16:0
TG 48:1; 14:0_16:0_18:1
TG 48:2; 14:0_16:0_18:2
TG 48:3; 12:0_18:1_18:2
TG 48:3; 16:1_16:1_16:1
TG 48:3; 14:0_16:0_18:3
TG 48:4; 12:0_18:2_18:2
TG 48:5; 12:0_18:2_18:3
TG 48:6; 12:0_18:3_18:3
TG 50:0; 16:0_16:0_18:0
TG 50:1; 16:0_16:0_18:1
TG 50:2; 16:0_16:0_18:2
TG 50:3; 16:0_16:0_18:3
TG 50:3; 16:0_16:1_18:2
TG 50:4; 14:0_18:2_18:2
TG 50:4; 16:0_16:1_18:3
TG 50:4; 16:1_16:1_18:2
TG 50:5; 14:0_18:2_18:3
TG 50:5; 16:0_16:2_18:3
TG 50:6; 14:0_18:3_18:3
TG 52:0; 16:0_18:0_18:0
TG 52:1; 16:0_18:0_18:1
TG 52:2; 16:0_18:0_18:2
TG 52:2; 16:0_18:1_18:1
TG 52:3; 16:0_18:1_18:2
TG 52:4; 16:0_18:2_18:2
TG 52:5; 16:0_18:2_18:3
TG 52:6; 16:0_18:3_18:3
TG 52:7; 18:1_16:2_18:4
TG 52:7; 16:1_18:3_18:3
TG 54:0; 14:0_16:0_24:0
-4
-2
0
2
4
6

(g)

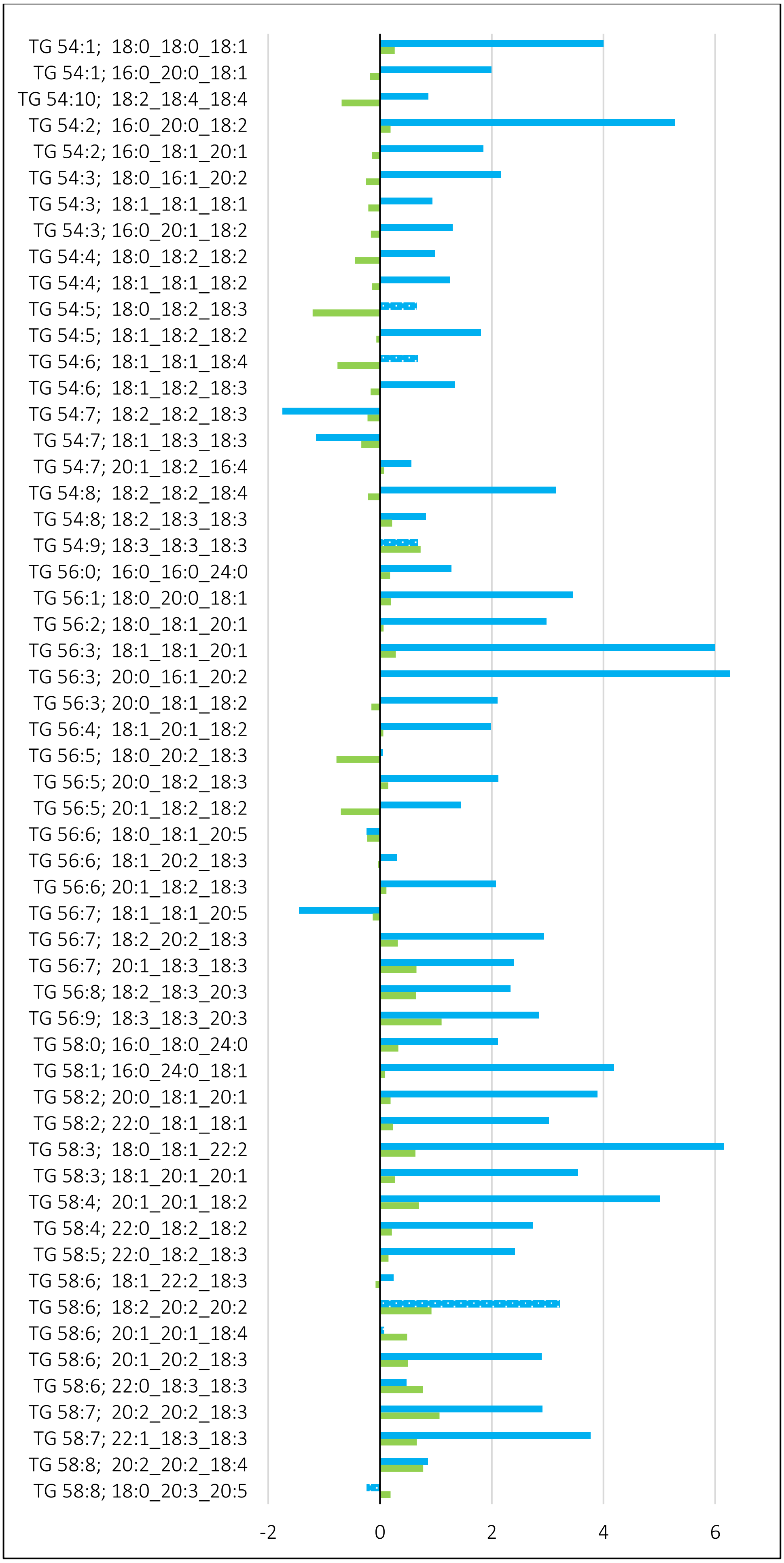

TG 54:1; 18:0_18:0_18:1
TG 54:1; 16:0_20:0_18:1
TG 54:10; 18:2_18:4_18:4
TG 54:2; 16:0_20:0_18:2
TG 54:2; 16:0_18:1_20:1
TG 54:3; 18:0_16:1_20:2
TG 54:3; 18:1_18:1_18:1
TG 54:3; 16:0_20:1_18:2
TG 54:4; 18:0_18:2_18:2
TG 54:4; 18:1_18:1_18:2
TG 54:5; 18:0_18:2_18:3
TG 54:5; 18:1_18:2_18:2
TG 54:6; 18:1_18:1_18:4
TG 54:6; 18:1_18:2_18:3
TG 54:7; 18:2_18:2_18:3
TG 54:7; 18:1_18:3_18:3
TG 54:7; 20:1_18:2_16:4
TG 54:8; 18:2_18:2_18:4
TG 54:8; 18:2_18:3_18:3
TG 54:9; 18:3_18:3_18:3
TG 56:0; 16:0_16:0_24:0
TG 56:1; 18:0_20:0_18:1
TG 56:2; 18:0_18:1_20:1
TG 56:3; 18:1_18:1_20:1
TG 56:3; 20:0_16:1_20:2
TG 56:3; 20:0_18:1_18:2
TG 56:4; 18:1_20:1_18:2
TG 56:5; 18:0_20:2_18:3
TG 56:5; 20:0_18:2_18:3
TG 56:5; 20:1_18:2_18:2
TG 56:6; 18:0_18:1_20:5
TG 56:6; 18:1_20:2_18:3
TG 56:6; 20:1_18:2_18:3
TG 56:7; 18:1_18:1_20:5
TG 56:7; 18:2_20:2_18:3
TG 56:7; 20:1_18:3_18:3
TG 56:8; 18:2_18:3_20:3
TG 56:9; 18:3_18:3_20:3
TG 58:0; 16:0_18:0_24:0
TG 58:1; 16:0_24:0_18:1
TG 58:2; 20:0_18:1_20:1
TG 58:2; 22:0_18:1_18:1
TG 58:3; 18:0_18:1_22:2
TG 58:3; 18:1_20:1_20:1
TG 58:4; 20:1_20:1_18:2
TG 58:4; 22:0_18:2_18:2
TG 58:5; 22:0_18:2_18:3
TG 58:6; 18:1_22:2_18:3
TG 58:6; 18:2_20:2_20:2
TG 58:6; 20:1_20:1_18:4
TG 58:6; 20:1_20:2_18:3
TG 58:6; 22:0_18:3_18:3
TG 58:7; 20:2_20:2_18:3
TG 58:7; 22:1_18:3_18:3
TG 58:8; 20:2_20:2_18:4
TG 58:8; 18:0_20:3_20:5
-2
0
2
4
6

(h)

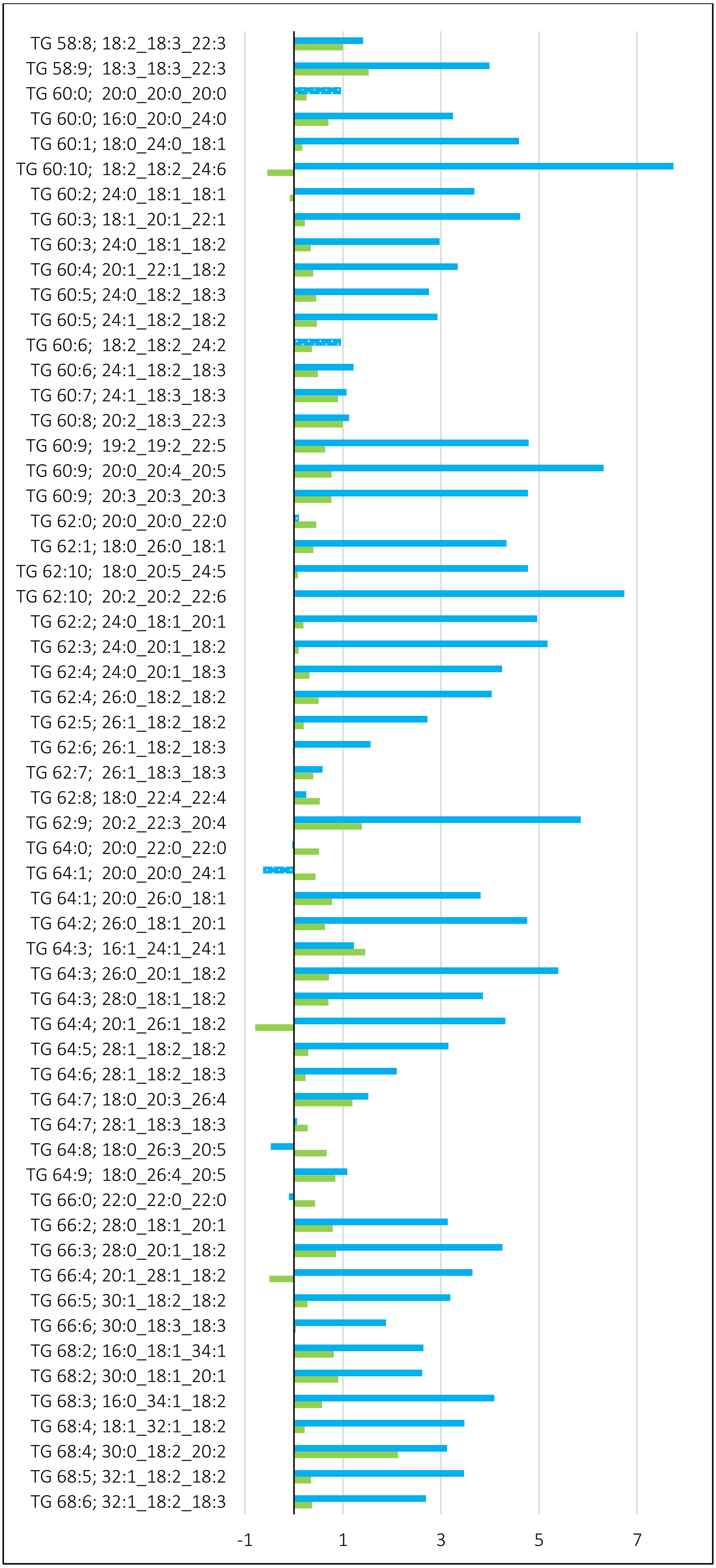
TG 58:8; 18:2_18:3_22:3
TG 58:9; 18:3_18:3_22:3
TG 60:0; 20:0_20:0_20:0
TG 60:0; 16:0_20:0_24:0
TG 60:1; 18:0_24:0_18:1
TG 60:10; 18:2_18:2_24:6
TG 60:2; 24:0_18:1_18:1
TG 60:3; 18:1_20:1_22:1
TG 60:3; 24:0_18:1_18:2
TG 60:4; 20:1_22:1_18:2
TG 60:5; 24:0_18:2_18:3
TG 60:5; 24:1_18:2_18:2
TG 60:6; 18:2_18:2_24:2
TG 60:6; 24:1_18:2_18:3
TG 60:7; 24:1_18:3_18:3
TG 60:8; 20:2_18:3_22:3
TG 60:9; 19:2_19:2_22:5
TG 60:9; 20:0_20:4_20:5
TG 60:9; 20:3_20:3_20:3
TG 62:0; 20:0_20:0_22:0
TG 62:1; 18:0_26:0_18:1
TG 62:10; 18:0_20:5_24:5
TG 62:10; 20:2_20:2_22:6
TG 62:2; 24:0_18:1_20:1
TG 62:3; 24:0_20:1_18:2
TG 62:4; 24:0_20:1_18:3
TG 62:4; 26:0_18:2_18:2
TG 62:5; 26:1_18:2_18:2
TG 62:6; 26:1_18:2_18:3
TG 62:7; 26:1_18:3_18:3
TG 62:8; 18:0_22:4_22:4
TG 62:9; 20:2_22:3_20:4
TG 64:0; 20:0_22:0_22:0
TG 64:1; 20:0_20:0_24:1
TG 64:1; 20:0_26:0_18:1
TG 64:2; 26:0_18:1_20:1
TG 64:3; 16:1_24:1_24:1
TG 64:3; 26:0_20:1_18:2
TG 64:3; 28:0_18:1_18:2
TG 64:4; 20:1_26:1_18:2
TG 64:5; 28:1_18:2_18:2
TG 64:6; 28:1_18:2_18:3
TG 64:7; 18:0_20:3_26:4
TG 64:7; 28:1_18:3_18:3
TG 64:8; 18:0_26:3_20:5
TG 64:9; 18:0_26:4_20:5
TG 66:0; 22:0_22:0_22:0
TG 66:2; 28:0_18:1_20:1
TG 66:3; 28:0_20:1_18:2
TG 66:4; 20:1_28:1_18:2
TG 66:5; 30:1_18:2_18:2
TG 66:6; 30:0_18:3_18:3
TG 68:2; 16:0_18:1_34:1
TG 68:2; 30:0_18:1_20:1
TG 68:3; 16:0_34:1_18:2
TG 68:4; 18:1_32:1_18:2
TG 68:4; 30:0_18:2_20:2
TG 68:5; 32:1_18:2_18:2
TG 68:6; 32:1_18:2_18:3
-1
1
3
5
7
