# Supplementary material for: An Arabidopsis lipid map reveals differences between tissues and dynamic changes throughout development
Source: Plant J. 2021 May 24;107(1):287–302. doi: 10.1111/tpj.15278 (PMC8361726; doi:10.1111/tpj.15278)
Supplement: Supplementary file 2 — Method S1. Growth protocol for Arabidopsis seedlings. Method S2. Growth protocol for Arabidopsis roots. Table S1. Recipe for germination medium by Conn et al. (2013). Table S2. Recipe for the standard growth solution by Conn et al. (2013). [file TPJ-107-287-s003.docx]

**Method S1: Growth protocol for seedlings**

Seeds were surface sterilized by washing them with 1 ml of 70% ethanol for 5 mins followed by 1 ml of sodium hypochlorite for 10 mins under constant shaking at room temperature. The seeds were thoroughly rinsed five times with 1 ml of sterile MilliQ water and plated on Petri dishes (90 x 15 mm) containing sterile solid ½ MS medium. The ½ MS medium contained of 0.5% Murashige and Skoog (1962) mineral salts (PhytoTechnology Laboratories, US), 0.05% of MES hydrate (Sigma), 1% (w/v) sucrose (Sigma) and 0.7% agar (Sigma). The pH was adjusted to 5.6-5.8 with 1N potassium hydroxide. The seeds were cold stratified for 3 days at 4 ^°^C before they were placed in a growth chamber under a 16 h light/8 h dark regime at 22 ^°^C and 50% relative humidity with a daytime light intensity of 100-120 µE. Seedlings were harvested after 7 d, 14 d and 21 d respectively, immediately frozen in liquid nitrogen and stored at -80 ^°^C until further use.

**Method S2: Growth protocol for roots**

To prepare root material, Arabidopsis plants were grown in a liquid medium using a system described by Conn *et al.* (2013). The germination medium (Table S1) and standard growth medium (Table S2) were prepared according to the instructions given by Conn *et al.* (2013). The lids of microcentrifuge tubes were punctured with a needle to form a 1.2-1.8 mm diameter hole in the middle of each lid. The lids were cut off from the tubes and placed on adhesive tape with the tape covering the holes. Each lid was then filled with the germination medium such that a dome is formed while ensuring the medium does not overflow and allowed to solidify. Then, the lids were removed from the tape and placed on the racks of 1 ml micropipette tip boxes filled with the liquid germination medium such that the plug of agar in each lid is in contact with the liquid medium. Twenty-eight lids were placed in one box, and empty holes in the racks were covered with aluminium foil to prevent light penetration. Then, two surface-sterilized Arabidopsis seeds were placed on the agar surface of each lid. The boxes were covered with plastic wrap and the seeds cold stratified at 4 ^°^C for 3 days. Next, they were placed in a growth chamber under 16 h light/8 h dark regime at 22 ^°^C and 50% relative humidity with a daytime light intensity of 100-120 µE. After 7 days, excess seedlings were removed to keep one seedling per hole, and the liquid medium was changed gradually to the standard growth solution as follows. On day 8, 30% of the germination medium was replaced with the standard growth solution, on day 9, 50% of the germination medium was replaced, and on day 10 the germination medium was entirely replaced by the standard growth medium. The plastic wrap was punctured on day 14 for the seedlings to adapt to the humidity in the chamber and completely removed after 17 days. The plants were grown for 28 d, with weekly solution changes before harvesting.

Table S1 The recipe for germination medium by Conn *et al.* (2013)

| **Macronutrients** | **Formula weight** | **g to make 1 L stock** | **Stock Concentration (M)** | **Volume of stock (mL) for 1L** | **Final concentration (mM)** |
| --- | --- | --- | --- | --- | --- |
| NH_4_NO_3_ | 80 | 80 | 1 | **0** | 0 |
| KNO_3_ | 101.1 | 101.1 | 1 | **0** | 0 |
| CaCl_2_ | 1M Solution |  | 1 | **0.75** | 0.75 |
| KCl | 74.55 | 74.55 | 1 | **1** | 1 |
| Ca(NO_3_)_2_•4H_2_0 | 236.1 | 94.4 | 0.4 | **0.625** | 0.25 |
| MgSO_4_•7H_2_0 | 246.5 | 98.6 | 0.4 | **2.5** | 1 |
| KH_2_PO_4_ | 136.1 | 13.61 | 0.1 | **2** | 0.2 |
|  |  |  |  |  |  |
| **Micronutrients** | **Formula weight** | **g to make 1 L stock** | **Stock Concentration (mM)** | **Volume of stock (mL) for 1L** | **Final concentration (uM)** |
| NaFe(III)EDTA | 367.1 | 18.4 | 50 | **1** | 50 |
| H_3_BO_3_ | 61.8 | 3.09 | 50 | **1** | 50 |
| MnCl_2_•4H_2_0 | 197.9 | 0.99 | 5 | **1** | 5 |
| ZnSO_4_•7H_2_0 | 287.5 | 2.875 | 10 | **1** | 10 |
| CuSO_4_•5H_2_0 | 249.7 | 0.125 | 0.5 | **1** | 0.5 |
| Na_2_MoO_3_ | 242 | 0.0245 | 0.1 | **1** | 0.1 |
| **pH with NaOH to 5.6** | |  |  |  |  |
| **0.7% Agar** | **7g per 1000ml** |  |  |  |  |
| Macronutrients | |  | Micronutrients |  |  |
| **Final concentration (mM)** | | **Activity** | **Final concentration (mM)** | | **Activity** |
| K | 1.2 | 4.79 | Fe | 0.01 | 25 pM |
| Ca | 1 | 1.05 | Mn | 0.005 | 23nM |
| Mg | 1 | 1.03 | Zn | 0.01 | 50 uM |
| NH_4_ | 0 | 1.72 | Cu | 0.0005 | 23 nM |
| Cl | 2.51 | 3.19 | Mo | 0.0001 | 31 nM |
| NO_3_ | 0.5 | 7.75 |  |  |  |
| SO_4_ | 1.0105 | 0.893 |  |  |  |
| PO_4_ | 0.2 | 1.8 pM |  |  |  |
| Na | 0.1012 | 1.38 |  |  |  |

Table S2 The recipe for the standard growth solution by Conn *et al.* (2013)

| **Macronutrients** | **FW** | **g to make 1 L stock** | **Stock concentration (M)** | **Vol of stock (mL) for 1L** | **Final concentration (mM)** |
| --- | --- | --- | --- | --- | --- |
| NH_4_NO_3_ | 80 | 80 | 1 | **2** | 2 |
| KNO_3_ | 101.1 | 101.1 | 1 | **3** | 3 |
| CaCl_2_ | 1M solution |  | 1 | **0.1** | 0.1 |
| KCl | 74.55 | 74.55 | 1 | **2** | 2 |
| Ca(NO_3_)_2_•4H_2_0 | 236.1 | 94.4 | 0.4 | **5** | 2 |
| MgSO_4_•7H_2_0 | 246.5 | 98.6 | 0.4 | **5** | 2 |
| KH_2_PO_4_ | 136.1 | 13.61 | 0.1 | **6** | 0.6 |
| NaCl | 58.44 | 58.44 | 1 | **1.5** | 1.5 |
| **Micronutrients** | **FW** | **g to make 1 L stock** | **Stock concentration (mM)** | **Vol of stock (mL) for 1L** | **Final concentration (uM)** |
| NaFe(III)EDTA | 367.1 | 18.4 | 50 | **1** | 50 |
| H_3_BO_3_ | 61.8 | 3.09 | 50 | **1** | 50 |
| MnCl_2_•4H_2_0 | 197.9 | 0.99 | 5 | **1** | 5 |
| ZnSO_4_•7H_2_0 | 287.5 | 2.875 | 10 | **1** | 10 |
| CuSO_4_•5H_2_0 | 249.7 | 0.125 | 0.5 | **1** | 0.5 |
| Na_2_MoO_3_ | 242 | 0.0245 | 0.1 | **1** | 0.1 |
| **pH with NaOH to 5.6** |  |  |  |  |  |
| Macronutrients | |  | Micronutrients |  |  |
| **Final concentration (mM)** | | **Activity** | **Final concentration (mM)** | | **Activity** |
| K | 5.6 | 4.79 | Fe | 0.01 | 25 pM |
| Ca | 2.1 | 1.05 | Mn | 0.005 | 23nM |
| Mg | 2 | 1.03 | Zn | 0.01 | 50 uM |
| NH_4_ | 2 | 1.72 | Cu | 0.0005 | 23 nM |
| Cl | 3.71 | 3.19 | Mo | 0.0001 | 31 nM |
| NO_3_ | 9 | 7.75 |  |  |  |
| SO_4_ | 2.0105 | 0.893 |  |  |  |
| PO_4_ | 0.6 | 1.8 pM |  |  |  |
| Na | 1.5502 | 1.38 |  |  |  |

**Conn, S.J., Hocking, B., Dayod, M., Xu, B., Athman, A., Henderson, S., Aukett, L., Conn, V., Shearer, M.K., Fuentes, S., Tyerman, S.D. and Gilliham, M.** (2013) Protocol: optimising hydroponic growth systems for nutritional and physiological analysis of Arabidopsis thaliana and other plants. *Plant Methods*, **9**, 4.
